# Supplementary material for: Population genomics and epigenomics of Spirodela polyrhiza provide insights into the evolution of facultative asexuality
Source: Commun Biol. 2024 May 16;7:581. doi: 10.1038/s42003-024-06266-7 (PMC11099151; doi:10.1038/s42003-024-06266-7)
Supplement: Supplementary file 2 — Supplementary_information [file 42003_2024_6266_MOESM2_ESM.pdf]

**Supporting Information for**

# Population genomics and epigenomics of *Spirodela polyrhiza* provide insights into the evolution of facultative asexuality

Yangzi Wang[1,2,#], Pablo Duchen[1,2,#], Alexandra Chávez[1,2,3], K. Sowjanya Sree[4], Klaus J. Appenroth[5], Hai Zhao[6], Martin Höfer[1,2], Meret Huber[1,3], Shuqing Xu[1,2,*]

1, Institute of Organismic and Molecular Evolution, University of Mainz, 55128, Mainz, Germany

2, Institute for Evolution and Biodiversity, University of Münster, 48161, Münster, Germany

3, Institute of Plant Biology and Biotechnology, University of Münster, 48161 Münster, Germany

4, Department of Environmental Science, Central University of Kerala, Kerala, 671320, India

5, Matthias Schleiden Institute — Plant Physiology, Friedrich Schiller University of Jena, 07743 Jena, Germany

6, Chengdu Institute of Biology, Chinese Academy of Sciences, 6100641 Chengdu, China

#: these authors contributed equally

*Corresponding author: Shuqing Xu

**Email:** shuqing.xu@uni-mainz.de

Table of Contents


1. Supplementary Methods ........................................................................ 4

1.1 Genome annotation update ........................................................................ 4

1.2 Structural variations identification in *S. polyrhiza* ........................................ 5

1.3 Identification of the MADS-box transcription factors ........................................ 6

1.4 Gene family identification in the *S. polyrhiza* genome ........................................ 7

1.5 The distribution and enrichment of INDELs and SVs among different gene families . 7

1.6 Demographic analysis ........................................................................ 8

1.7 Differentially methylation region analysis ........................................ 10

1.8 Differentially expression analysis based on RNA-seq data ........................................ 10

1.9 Gene expression analysis with RT-qPCR ........................................ 11

2. Supplementary Results ........................................................................ 13

2.1 Update of *S. polyrhiza* annotation ........................................ 13

2.2 Structural variation in the *S. polyrhiza* population ........................................ 13

2.3 MADS-box transcription factor family in *S. polyrhiza* ........................................ 14

2.4 Population demographic history ........................................ 14

2.5 Differentially methylated regions (DMRs) among four populations of *S. polyrhiza* ... 15

2.6 Population/branch-specific selection scans ........................................ 15

2.7 Differentially expressed genes in the shoot tissues among four populations of *S. polyrhiza* ........................................ 16

2.8 Authenticity validation of candidate genes that were under selection ........................................ 17

3. Supplementary Figures ........................................................................ 20

**4. Supplementary Tables** .......... 49
**5. Supplementary Reference** .......... 65

# 1. Supplementary Methods

## *1.1 Genome annotation update*

To improve the *S. polyrhiza* genome annotation, we developed a pipeline integrating short- and long-read transcriptomic data, available proteomes, and *ab initial* gene predictions (Supplementary Figure 18). RNA-seq short reads from *S. polyrhiza* frond, root, and whole plant[1] were downloaded from NCBI and filtered using Skewer (v0.2.2)[2]. After the filtration, they were mapped to the reference genome of *S. polyrhiza* using HISAT2 (v2.2.1)[3] with the "--dta" mode. SAMtools (v1.10)[4] was used for bam file sorting and indexing. Scallop (v0.10.5)[5] was used to assemble the transcripts. Iso-seq data[1] were downloaded from NCBI, and the full-length transcripts were aligned to the *S. polyrhiza* reference genome using minimap2 (v2.21)[6] under the "-x splice:hq" mode. Only mapped transcripts were kept as empirical evidence in the Maker pipeline.

High-quality proteomes from *Oryza sativa* (v7.0, Phytozome 13)[7], Maize (AGPv3.22, MaizeGDB)[8], *Arabidopsis thaliana* (TAIL10)[9], and *Zostera marina* (V2, OrcAE)[10] were included as the empirical evidence for the Maker pipeline to predict gene models.

We combined Maker (version 2.31.10)[11] and Braker (version 2.1.5)[12] pipelines for annotating the gene models (Supplementary Figure 18). One round of SNAP *ab initio* gene prediction was performed based on the repeat soft-masked reference genome of *S. polyrhiza*. Results from the Braker pipeline and the previous annotation Sp7498V2 were provided to Maker as "legacy annotations". After that, the transcript model from the iso-seq data was used to correct the gene models. BLAST+ (v2.11.0)[13] and InterProScan (version 5.50-84.0[14], running under Java/11.0.2) were used to perform functional and Gene Ontology (GO) annotation (Supplementary Figure 18).

To ascertain the orthologous gene relationships between *S. polyrhiza* and *A. thaliana*, we employed the 'getRBH.pl' script[15]. We defined gene pairs showcasing reciprocal best hits (RBH) between the two species as orthologs.

## 1.2 Structural variations identification in *S. polyrhiza*

We adopted the joint genotyping pipeline (Supplementary Figure 21) from Eggertsson *et al.*[16] to call SVs. Compared to the original method in which Manta was the only SV caller, we added another four popular SV callers in the pipeline. We found that this method could make full use of Manta's high sensitivity while keeping the false positives relatively low. Further validation based on the long-read sequencing data suggested that this method provided the most confident SV dataset when compared with other prevailing methods.

For each *S. polyrhiza* sample, Manta[17], Smoove (Lumpy calling and svtyper genotyping, https://github.com/brentp/smoove)[18,19], GRIDSS[20], Delly[21], and SvABA[22] were used independently to call individual-level SVs. After that, all SVs from each caller were merged using svimmer's "join mode". For each sample, we kept only SVs called by both Manta and at least one other caller. After that, we generated a population-level SV call set using svimmer, which was then used for genotyping with GraphTyper2[16].

We applied several filtrations to the population-level genotyped SV set. First, complex SVs and SVs from organelle genomes were removed. Second, SVs with a size less than 50 bp or larger than 500 kb were removed, as small SVs were included in the GATK pipeline, and large SVs were difficult to confirm. Third, deletions that contain assembly gaps were removed due to uncertainties in the genome quality; Fourth, SVs with minor allele frequency lower than 0.01 or higher than 60% of missing genotypes were removed; Fifth, for duplications, only genotypes with "Genotyping Quality (GQ)" higher or equal to 20 were kept. We didn't apply the default genotyping filtration from GraphTyper2 because its strict filtration criteria would remove a high proportion of positive calls[16]. We used BCFtools[23], bgzip, and tabix[24] for most of the Variant Call

Format (VCF) file manipulations. The filtrations were done using BCFtools, VCFtools, and VcfFilter (https://github.com/biopet/vcffilter).

To estimate population structure using SVs, we converted the VCF file into PLINK format using VCFtools[25]. The PCA was carried out using PLINK, while the population structure analysis was estimated using fastStructure. For the SV-based phylogenetic tree reconstruction, we converted SVs into a p-distance matrix using VCF2Dis (https://github.com/BGI-shenzhen/VCF2Dis). The PHYLPNEW program from EMBOSS[26] was used to build the neighbour-joining tree. The R package "ggtree" (v3.2.1)[27] was used for tree visualizations. SV alignments were visualized using Samplot[28], and gene models were plotted using JBrowser2[29].

### 1.3 Identification of the MADS-box transcription factors

All protein-coding genes from SpGA2022 annotation were aligned with the MADS-box genes from *O. sativa*[30] and *A. thaliana*[9] using BLAST+ (v2.11.0)[13] with an e-value threshold of 1e-5. We search against the SRF- and MEF2-Type MADS domains using "hmmsearch" from HMMER (v3.3.2, http://hmmer.org/)[31]. Results from those two processes were combined and further filtered using the NCBI Conservative Domain Database (CDD)[32] (Supplementary Figure 25). As a result, 43 MADS-box genes were identified in *S. polyrhiza* (Supplementary Data 12).

To annotate the identified 43 *S. polyrhiza* MADS-box genes, we collected annotation information based on either TAIR (www.arabidopsis.org) or previous studies of the MADS-box gene family in *A. thaliana*[33], *O. sativa*[34], *Ananas comosus*[35], *Saccharum spontaneum*[36], and *Nelumbo nucifera*[37]. In total, 223 MADS-box gene coding proteins from *A. thaliana*, *O. sativa*, and *S. polyrhiza* were aligned using MAFFT (v7.490)[38]. Phylogenetic trees of type I and type II MADS-box families were reconstructed using FastTree (v2.1.11)[39] with default parameters. According to the phylogenetic clustering, 43 MADS-box genes were then classified into 14 clades (Supplementary Table 10). Tree visualization and annotation were done using the "ggtree" package (v3.2.1)[27] with R (v4.1.0).

## 1.4 Gene family identification in the *S. polyrhiza* genome

We used a step-wise approach to characterize gene families in *S. polyrhiza* genome. First, we used OrthoFinder (v 2.5.5)[40] to identify putative ortholog groups. Protein sequences were obtained by *in silico* translating the longest isoforms from all predicted CDS of *Solanum lycopersicum*, *Sorghum bicolor*, *Nymphaea colorata*, *Musa balbisiana*, *Arabidopsis thaliana*, and *Oryza sativa*. Second, we used the gene family database to group ortholog groups into gene families. To this end, we used gene families database from *Arabidopsis* (https://www.arabidopsis.org/browse/genefamily/index.jsp)[41]. When *Arabidopsis* genes from different ortholog groups were annotated into one gene family, we merged the ortholog groups into one and annotated them according to the Arabidopsis gene family. For the remaining unannotated orthogroups, we considered that all *S. polyrhiza* genes in each individual orthogroup represent a distinct gene family. For these unannotated gene families, we checked the functional annotations of all the members. We retained orthogroups/gene families that have more than four genes for downstream analyses. In total, 5,043 genes were assigned into 256 orthogroups/gene families (Supplementary Data 4).

## 1.5 The distribution and enrichment of INDELs and SVs among different gene families

The enrichment tests of INDELs and SVs with different gene families were done using permutation tests wrapped in the regioneR package (v1.32)[42]. Gene families with no less than 10 members were involved in the enrichment analysis. The fraction of genes (as well as their upstream 2Kb regions) overlapped with INDELs or SVs were considered as the evaluation function. In the regioneR package, we used randomization method "randomizeRegions". Repetitive regions and assembly gaps (Ns) were excluded (masked) during the randomization, as these regions do not have genes. For each gene family, 5,000 permutations were used.

### 1.6 Demographic analysis

To infer the demographic history of our sampled populations of *S. polyrhiza,* we used Approximate Bayesian Computation (ABC), which makes use of observed data, candidate demographic models, and simulations of the observed data under the candidate models to infer population demographic parameters such as population sizes, migration rates, and colonization times.

Data consists of SNPs coming from intergenic regions of the four populations (SE-Asia, North America, Europe, and India) of *S. polyrhiza*. From each population, we selected 17 non-clonal individuals (the minimum number observed, which corresponds to the American population) to ensure all populations have the same number of individuals for downstream analyses. To select intergenic regions for demographic inference, we performed the following steps. First, we obtained the coordinates of all intergenic loci based on our latest annotation. Then, we removed all loci that overlapped with structural variants. Then, we sorted the loci by size and by chromosome and selected the three largest loci from each chromosome. We manually inspected the resulting alignment for each locus and discarded the loci with a large percentage of missing data or no SNPs available. A total of 39 intergenic loci passed all the filters described above, averaging two loci per chromosome (Supplementary Table 4). This filtering procedure is ideal for demographic inference because it keeps a few loci per chromosome (thus increasing the independence between loci, which is required in coalescent simulations) while keeping a large number of SNPs. All 39 intergenic loci yielded a total of 13,065 SNPs for demographic inference. From each of these loci, we calculated the following summary statistics: number of segregating sites S, Waterson's theta, pi, Tajima's D, Fu and Li's D, Fay and Wu's H, average LD (ZnS), the site-frequency spectrum (SFS) per population, the joint SFS (JSFS) for each pair of populations, and the summaries of the JSFS as described by Wakeley and Hey[43].

We tested three demographic models (Supplementary Figure 4). Model 1 consists of an American origin, with subsequent colonization of Asia, and then from Asia independent colonization of Europe and India. Model 2 starts with Asia as the ancestral population, then colonization to America and further independent colonization of Europe and India from Asia. Model 3 consists of a putative ancestral population, then colonization to America and Asia, and subsequent colonization of India and Europe from Asia. Migration was allowed between all current populations.

For each of the models described above, we performed 50,000 coalescent simulations with the software *msms*[44]. This program simulates coalescent trees for each population under any given demographic model while accounting for selection. The output of *msms* are SNPs from which we calculated the following summary statistics: number of segregating sites S, Waterson's theta, pi, Tajima's D, Fu and Li's D, Fay and Wu's H, average LD (ZnS), the site-frequency spectrum (SFS) per population, the joint SFS (JSFS) for each pair of populations, and the summaries of the JSFS as described by Wakeley and Hey[43]. These same statistics were calculated for the observed loci described below.

The model choice was performed within an ABC framework. Posterior probabilities for each model were calculated according to Fagundes *et al.*[45]. Model choice was done based on the mean and variance (across loci) of the number of segregating sites $S$, Tajima's $D$, linkage disequilibrium ($Z_{\mathrm{nS}}$), and population differentiation statistics in all four populations. In our analysis, Watterson's $\Theta_{\mathrm{W}}$, $\Pi_n$, and $K$ were correlated with $S_n$, and therefore its inclusion does not change the results of the model choice procedure. When comparing all models, the model with the highest posterior probability was chosen as the best fit for the observed data.

On the best model, the inference was based on ABC rejection[46,47] and regression[48] methods. Both methods were performed using *ABCtoolbox*[49] and checked with Csilléry's abcR[50]. First, we pooled all statistics and checked for correlations with the parameters. We did not keep statistics that did not correlate with any parameter because keeping them does not provide

information for the estimation and would only add noise to the final estimates. All these statistics were transformed using partial least squares (p.l.s.) as implemented in Wegmann *et al.*[49]. This transformation is advantageous because it extracts a small number of orthogonal components from a higher dimensional array of summary statistics. The new set of transformed statistics (with reduced dimensionality) reduces the noise produced by uninformative summary statistics. Moreover, the p.l.s.-transformed statistics are completely uncorrelated with one another, ensuring the assumption of singularity, which is required for estimating parameters according to the regression method[48].

**1.7 Differentially methylation region analysis**

For the differential methylation region (DMR) analysis, we employed methylKit[51]. CpG wML over *S. polyrhiza* genome's tiling windows (with 10 Kb window size and 1Kb step size) were compared pairs of populations including SE-Asia vs. Europe, SE-Asia vs. India, Europe vs. India, and SE-Asia vs. America populations, respectively. The cutoff for identifying the wML difference of a given window is 25 (the default from methylKit).

**1.8 Differentially expression analysis based on RNA-seq data**

To explore the transcriptional landscape and validate the candidate genes involved in asexual reproduction, we performed RNA-seq on shoot tissues from 19 distinct genotypes. Those genotypes were the same genotypes involved in the whole-genome bisulfite sequencing that we described previously, except for genotype SP014 due to sequencing failure. We processed the paired-end RNA-seq data using Trimmomatic (version 0.39)[52], applying parameters to trim the initial 10 bases and to remove any potential Illumina adapter sequences: "ILLUMINACLIP:TruSeq3-PE-2.fa:2:30:10 SLIDINGWINDOW:4:15 MINLEN:36

HEADCROP:10”. The quality of the pre- and post-trimming RNA-seq reads was assessed using FastQC (version 0.11.9, available at https://www.bioinformatics.babraham.ac.uk/projects/fastqc/). Subsequent to quality control, we aligned the high-quality reads to the *S. polyrhiza* reference genome using HISAT2 (version 2.2.1)[3]. We quantified expression levels with featureCounts[53], and genes were considered expressed if they had an expression count of four or more in at least three distinct genotypes. Differential expression analysis across population pairs—SE-Asia vs. Europe, SE-Asia vs. India, Europe vs. India, and SE-Asia vs. America—was conducted using the DESeq2 package[54]. Significance thresholds were established at a log2 fold change > 1 or < -1 and an adjusted p-value below 0.05.

**1.9 Gene expression analysis with RT-qPCR**

We examined the expression of eight candidate genes that were under selection in genotypes SP004 and SP012. Five replicates were made for each genotype. For each replicate, ten fronds were grown in plastic beakers filled with 150 ml of N-medium for five days at 26 °C, 70 µmol photons/$m^2$*s and 16h/8h light-dark rhythm. Plants were harvested after five days, and the roots were surgically removed from the frond tissue. We used < 20 mg of ground frond tissue for RNA extraction following the instructions of the InnuPREP RNA mini kit (Analytik Jena). We performed cDNA synthesis with Oligo-dT primers following the instructions of the RevertAid First Strand cDNA synthesis kit (Takara). For each reaction, we used ~ 1 µg of RNA. The primer efficiencies were determined by a serial dilution series of cDNA templates (Supplementary Table 11). We examined the primer specificity by checking the amplicons of the qPCR reactions on a two percent agarose gel. For all qPCR reactions, we used the Mastermix from the KAPPA SYBR FAST kit (Roche). All reactions were conducted on a RotorGene Q system (Qiagen) with the following program: 98 °C for three minutes followed by 40 cycles of a denaturation step at 98 °C for 3 s and an annealing/extension step at 60 °C for 20 s. The expression of the candidate genes

was visualized as delta Ct values relative to the arithmetic mean of the Ct values of the reference genes ALPHA ELONGATION FACTOR ONE (*aEF*) and GLYCERIN-ALDEHYDE-3-PHOSPHATE DEHYDROGENASE (*GAPDH*) according to a published method[55].

## 2. Supplementary Results

### 2.1 Update of S. polyrhiza annotation

Using recently published transcriptomic data and proteomes, we updated the genome annotation of *S. polyrhiza* using Maker (version 2.31.10)[11] and Braker (version 2.1.5)[12] (Supplementary Figure 18). We predicted gene models using isoform sequencing (Iso-seq) reads, short-read transcriptomes from different tissues of *S. polyrhiza* and high-quality proteomes from closely related lineages or well-established model species as empirical evidence. Although the total number of annotated genes only slightly increased (926 genes more than Sp7498V2), the new annotation (SpGA2022) reaches higher completeness and is less fragmented than the previous annotation (Supplementary Table 9 and Supplementary Figure 19). The DOGMA[56] score increased from 71.9 to 79.3 (Supplementary Figure 20).

### 2.2 Structural variation in the *S. polyrhiza* population

We characterized genome-wide structural variations (SVs) using an adopted joint genotyping pipeline[16] (Supplementary Figure 21). Using long reads from genotype SP014 (strain 9509)[57], we evaluated the performed precision-recall (PR) of our pipeline. The precisions of insertions and deletions identified based on short reads reached 28.0% and 64.5%, respectively, which are comparable with that found in a recent study focused on soybean population genomics[58]. The high consistency of population structures estimated based on SVs and SNPs often indicates high accuracy of SV characterizations[59,60]. In the PCA analysis using the identified SVs, we found that four populations were separated according to the first three PCs (Supplementary Figure 22), consistent with the pattern observed using SNPs. Using fastStructure with SVs, we also identified four populations (Supplementary Figure 23) with the same genotype assignment as with the analysis using SNP data. The clustering pattern using SVs and SNPs also showed similar patterns (Supplementary Figure 24).

In total, we identified 2,089 deletions, 825 insertions, and 291 duplications in the *S. polyrhiza* genomes.

## 2.3 MADS-box transcription factor family in *S. polyrhiza*

The MADS-box family controls different developmental processes, including root, leaf, flower, and fruit development[61,62]. *Spirodela polyrhiza* has lost many MADS-box TFs clades[63], which might be associated with the reduced size and simplified architecture[64]. Here, we reannotated 43 MADS-box genes in the *S. polyrhiza* genome. Based on the phylogenetic relationship, we classified these MADS-box TFs into 14 clades (Supplementary Table 10), three from the Type I subfamily (Supplementary Figure 26), and 11 from the Type II subfamily (Supplementary Figure 27). Consist with the previous study[64], we found that *AGL15*-, *FLC*-, *AGL9*- *AGL12*-, and *OsMADS32*-like clades are missing in the *S. polyrhiza* genome.

## 2.4 Population demographic history

We developed three competing demographic models using coalescent simulations to provide further insights into the population's demographic history.

*Model choice*

Our ABC-based model selection procedure favors Model 3 (Posterior Probability; P=0.88) as the most probable model, compared to Model 1 (America-origin, P=0.027) and Model 2 (Asia-origin, P=0.097) (Supplementary Figure 4). this result implies that the sampled American and SE-Asian populations are equally distant from a putative ancestral population, and this is consistent with the phylogenetic observation, where the branch lengths of the American and the SE-Asian clades are very similar.

*Parameter inference*

After performing ABC-based parameter inference on Model 3, we found that the SE-Asian population has the largest *Ne*, followed by the American, Indian, and European populations (Supplementary Table 5). Concerning the colonization times, both our sampled American and SE-Asian populations split from an ancestral population ca. one million generations ago. Then, the Indian and European populations split from the SE-Asian branch ca. 51,000 and 12,000 generations ago, respectively (Supplementary Table 5). Finally, migration in and out of Europe is the highest, followed by migration between Asia and America and in and out of India (Supplementary Table 5).

### 2.5 Differentially methylated regions (DMRs) among four populations of *S. polyrhiza*

To test for associations between genomic selection and the epigenomic variations, we have analyzed the CpG differentially methylation regions (DMR) between populations of SE-Asia vs. America, SE-Asia vs. India, SE-Asia vs. Europe, and Europe vs. India. From this DMR analysis we found a total of 302 genes that are differentially methylated between pairs of populations (116 DMR genes from SE-Asia vs. America, 43 DMR genes from SE-Asia vs. Europe, 60 DMR genes from SE-Asia vs. India, and 83 DMR genes from Europe vs. India). Out of these 302 genes, 17 are found to have been under selection in specific populations according to our 3P-CLR results (Supplementary Data 10).

### 2.6 Population/branch-specific selection scans

Selection scans with 3P-CLR (using the American population as an outgroup) identified a total of 1883 genes on the SE-Asian branch, 593 genes on the Indian branch, and 401 genes on the European branch (Fig. 4a), corresponding to the top 1% of CLR values on each population branch (Supplementary Data 9). Of these genes, 77 were common between Europe and India, 18 between

SE-Asia and Europe, 32 between SE-Asia and India, and 12 in all three populations (Fig. 2b and Supplementary Data 9). We tested the significance of the largest gene overlap (India and Europe) in comparison with the genomic background (all annotated genes from 20 chromosomes) and found a *P-value* < 2.2e-16 (Fisher's Exact Test). In addition to the three genes mentioned in the main text, [SpGA2022_013448 (*FLK*-like), SpGA2022_006111 (*BB*-like) and SpGA2022_055195 (*CYP78A9*-like)], several other genes are also likely involved in flower and organ development. SpGA2022_006114 (chromosome 3), annotated as *PFP*, is a phosphofructokinase with higher expression in flowers and fruits than in roots and leaves[65]. SpGA2022_052120 (chromosome 4), annotated as *ARF1* Binding protein, encoding an auxin response factor, and is involved in floral organ abscission and positive regulation of flower development[66]. SpGA2022_016777 (chromosome 13), annotated as *SOMBRERO*, is involved in auxin signaling, negatively affecting the cellular re-specification at the root tip, where *SOMBRERO* orchestrates both the formation of extra root cap layers and primary root growth under phosphate scarcity[67].

In addition to the five MADS-box genes that were under positive selection in the Indian population, two MADS-box genes (SpGA2022_013026 and SpGA2022_013226) were also under selection in the SE-Asian population.

**2.7 Differentially expressed genes in the shoot tissues among four populations of *S. polyrhiza***

We found 326 differentially expressed genes (DEGs) between SE-Asia and America populations, 384 DEGs between SE-Asia and Europe, 301 DEGs between SE-Asia and India, and 85 DEGs between Europe and India. In our research, we aimed to reveal if the observed DEGs could be attributed to population-specific selection. To this end, we performed an intersection analysis between the DEGs and genes showing signatures of selection within the corresponding

populations (Supplementary Data 11). Notably, within the 85 DEGs identified between the Europe and India comparisons, 9 genes also exhibited selection signatures within the European or Indian populations. This enrichment is significantly greater than what would be expected by random chance (P=0.038, Hypergeometric test), suggesting that the DEGs may be the consequence of natural selection acting within each population.

Further investigation of these 9 genes revealed the presence of SpGA2022_055195, an ortholog of Arabidopsis *CYP78A9*, known to play a critical role in promoting cell proliferation during floral development and influencing seed size. Another gene, SpGA2022_006116, homolog to Arabidopsis *OVA5*, is implicated in encoding a lysyl-tRNA ligase with dual localization in the mitochondrion and chloroplast, with mutants exhibiting ovule abortion. Given their functions, we hypothesize that these two genes are likely involved in flowering pathways. The fact that they are both under selection and differentially expressed across populations further supports our hypothesis that selection may influence sexual reproduction-related genes, consequently affecting sexual reproduction rates across populations.

**2.8 Authenticity validation of candidate genes that were under selection**

We validated the expression of eight candidate genes that were under positive selection in frond tissue of two genotypes (SP004 and SP012) using RT-qPCR (Supplementary Figure 28). In the RT-qPCR experiment, six genes showed expression in fronds tissue in both genotypes, confirming their authenticity. Two genes (*AGL62* and *AGL6*) showed no expression (Supplementary Table 12) in the frond tissue, as assessed by qRT-PCR and RNA-seq (Supplementary Data 7). We hypothesize that this lack of expression in the sampled tissues may be due to these genes being involved in embryogenesis and thus potentially having specific expression in flower tissues, which was not included in our tissue sampling. Among all tested

candidate genes, we find *RbgA* was differentially expressed between the flowering genotype SP004 and the non-flowering genotype SP012 (P=0.0079, Wilcoxon Rank sum test, Supplementary Figure 28).

## 3. Supplementary Figures

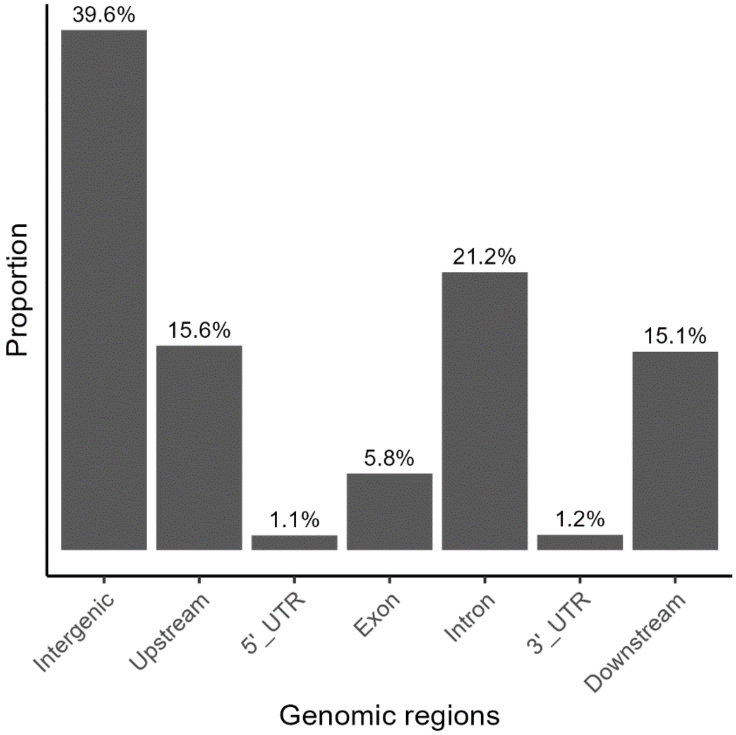


**Supplementary Fig. 1. SNP distributions among different genomic regions.**

The annotation of SNPs was calculated using SnpEff. "Upstream" and "downstream" indicate the flanking 2 kb regions of annotated genes. The intergenic region did not take upstream and downstream information into account.

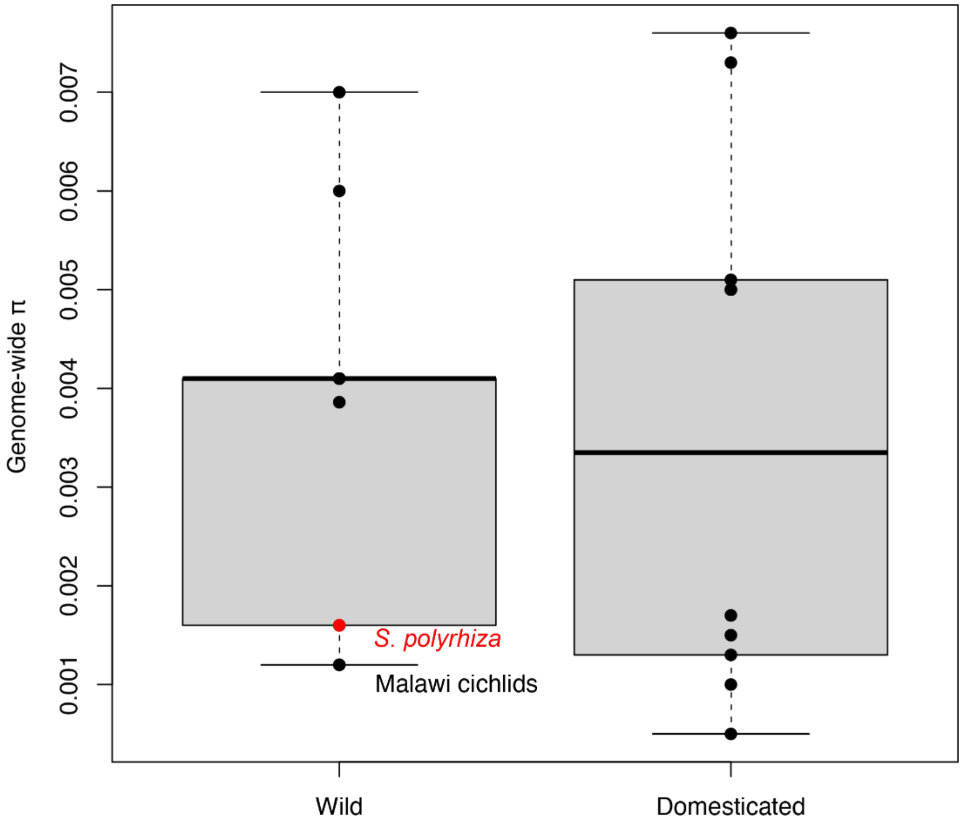


**Supplementary Fig. 2. Genome-wide nucleotide diversity ($\pi$) in wild and domesticated organisms listed in Supplementary Table 13.** The red dot represents the $\pi$ of *S. polyrhiza* in the present study ($\pi$ = 0.0016). The lowest $\pi$ reported is in Malawi cichlids.

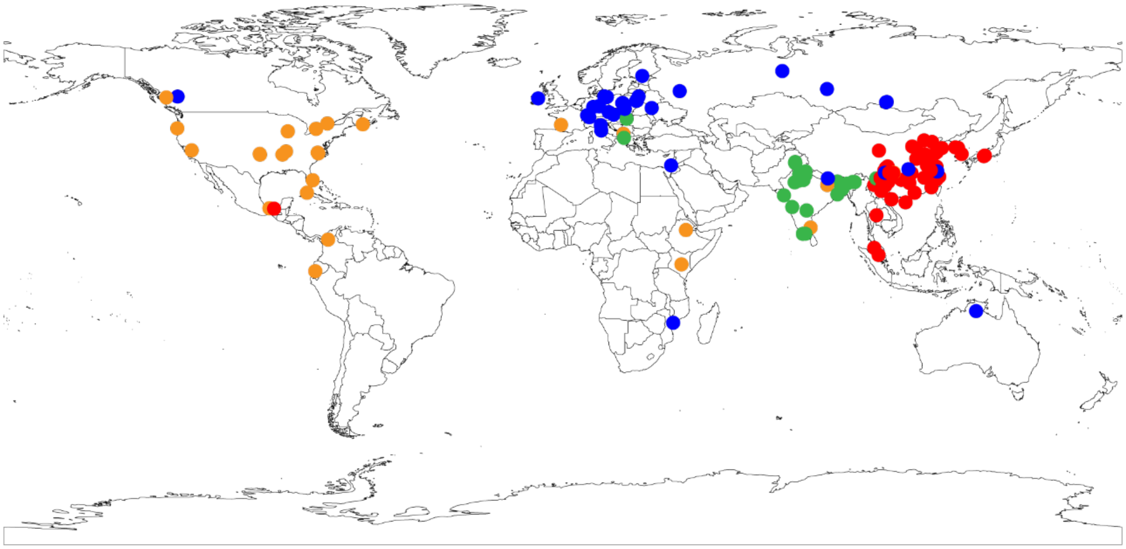

**Supplementary Fig. 3. Worldwide geographic distribution of 228 *S. polyrhiza*.**

Circular dots indicate the geographic locations from where the samples were collected. Those dots are colored according to the genetic population stratification: SE-Asian population (red), Indian population (green), European population (blue), and American population (yellow).

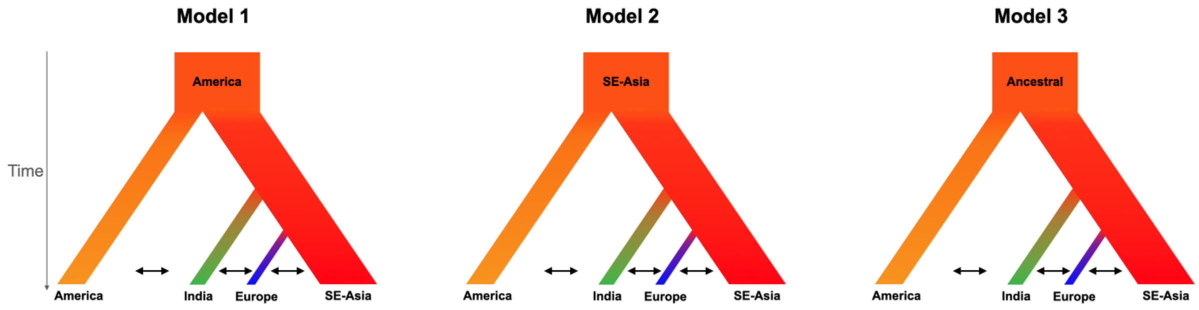


**Supplementary Fig. 4. Demographic models tested with ABC.**

Model 1 (American origin) had a posterior probability of P=0.027, Model 2 (SE-Asia origin) P=0.097, and Model 3 P=0.88.

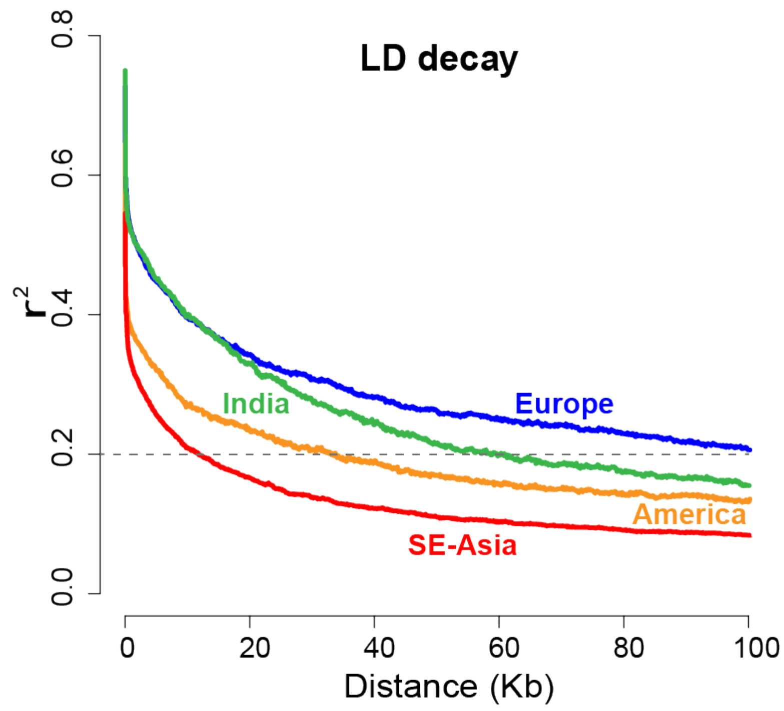


**Supplementary Fig. 5. The decay of linkage disequilibrium in *S. polyrhiza*.**

Curved lines in different colors indicate the LD decay in all four populations. The grey dashed line represents an $r^2$=0.2 used for comparison.

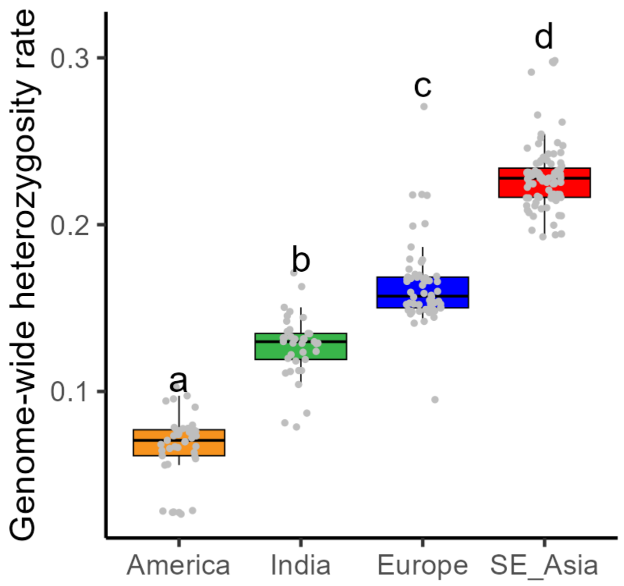


**Supplementary Fig. 6. Comparison of genome-wide heterozygosity rate among four populations.**

Grey dots indicate the intra-individual heterozygosity rate of each genotype. Lowercase characters show the significance tested using the Wilcoxon test.

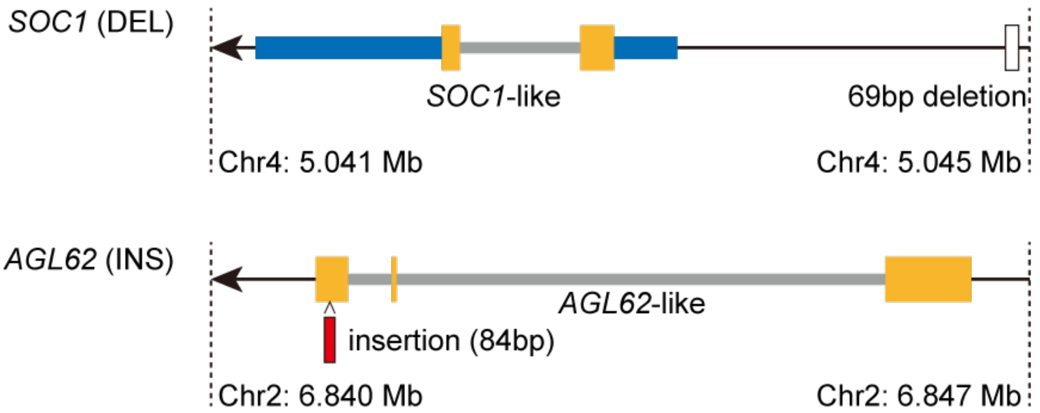


**Supplementary Fig. 7. Schematic of the SVs impacting on two MADS-box genes.**

SVs affect two MADS-box genes, SpGA2022_007306 (*SOC1*-like) and SpGA2022_005278 (*AGL62*-like), that are related to sexual reproduction. The yellow blocks indicate the exons, the blue blocks indicate the UTRs, and the grey ones indicate the introns. The white rectangle indicates the location of the deletion variation, while the red one indicates the insertion.

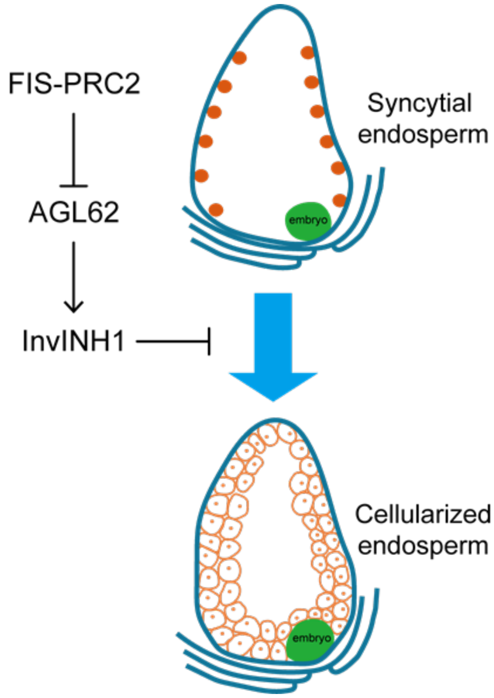


**Supplementary Fig. 8. Schematic illustration of the putative *AGL62* pathway.**

A black arrow indicates activation, while a bar at the end of a line indicates repression. The thick blue arrow indicates the transition from the syncytial phase to the cellularization phase of the endosperm.

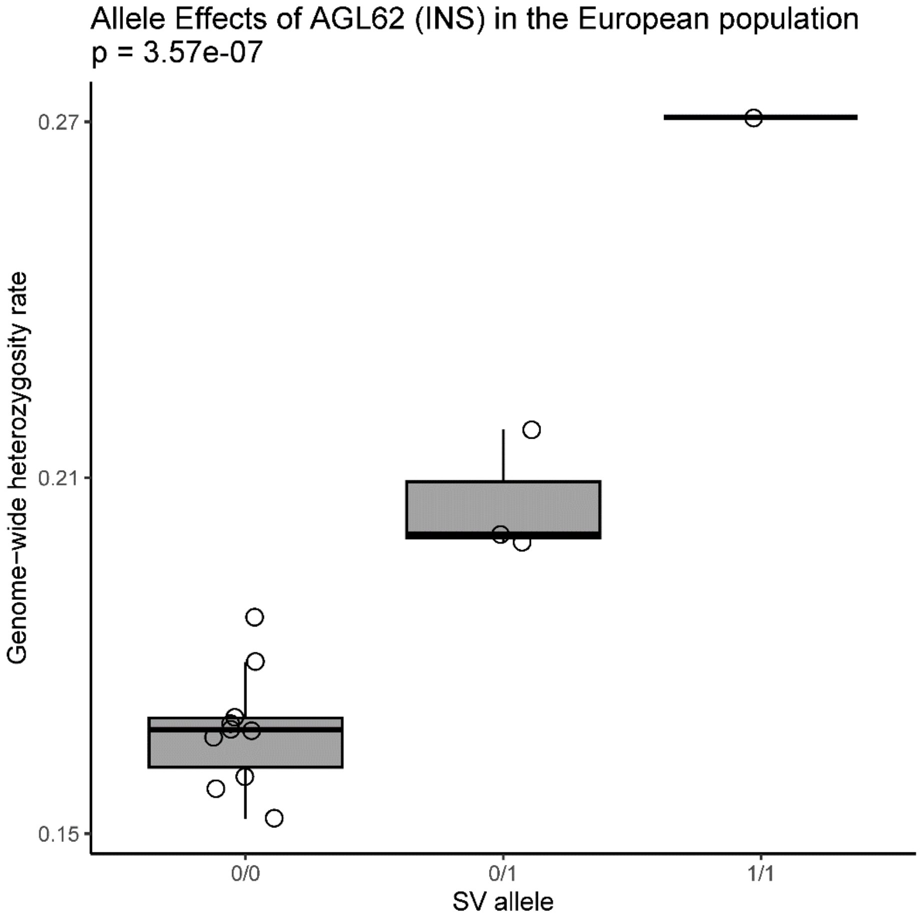


**Supplementary Fig. 9. Allele effects of the insertion variation that affect gene *AGL62* in the European population.**

Grey circles indicate the intra-individual heterozygosity rate. “0/0” refers to the homozygous reference allele, “0/1” refers to the heterozygous allele, and “1/1” refers to the homozygous alternative allele (allele that is impacted by the SV). P value was estimated from the genetic association using RVTESTS.

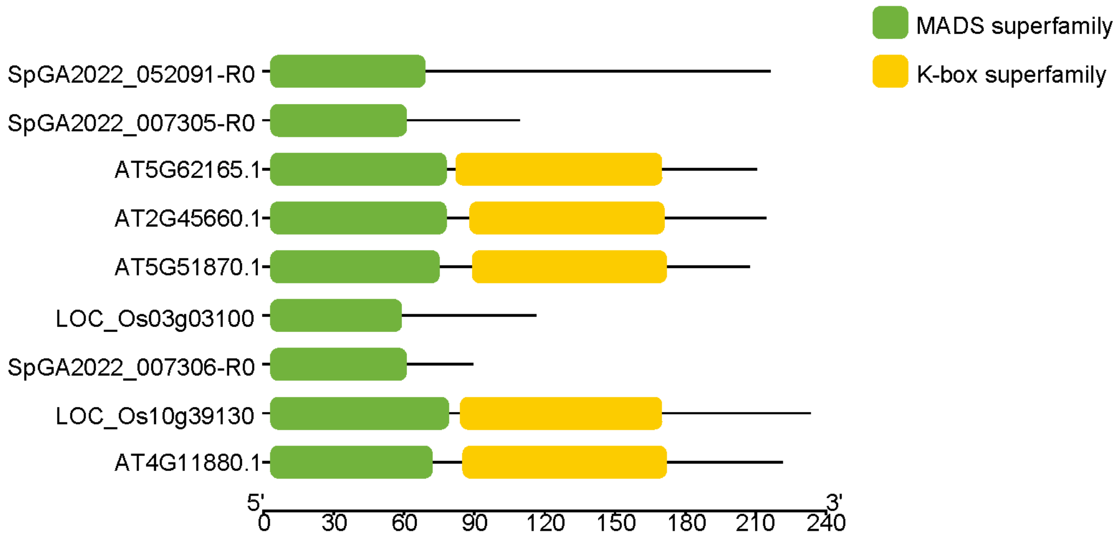


**Supplementary Fig. 10. Comparison of conserved domains among putative *S. polyrhiza SOC1* homologs and *SOC1* from *Arabidopsis thaliana* and rice.**

Black lines indicate protein sequences, while colored boxes indicate conserved domains which were identified using NCBI CDD search tool. Proteins which start with "AT" are *Arabidopsis SOC1* proteins, while "LOC_Os" are rice *SOC1* proteins, and "SpGA2022" are putative *S. polyrhiza SOC1* proteins.

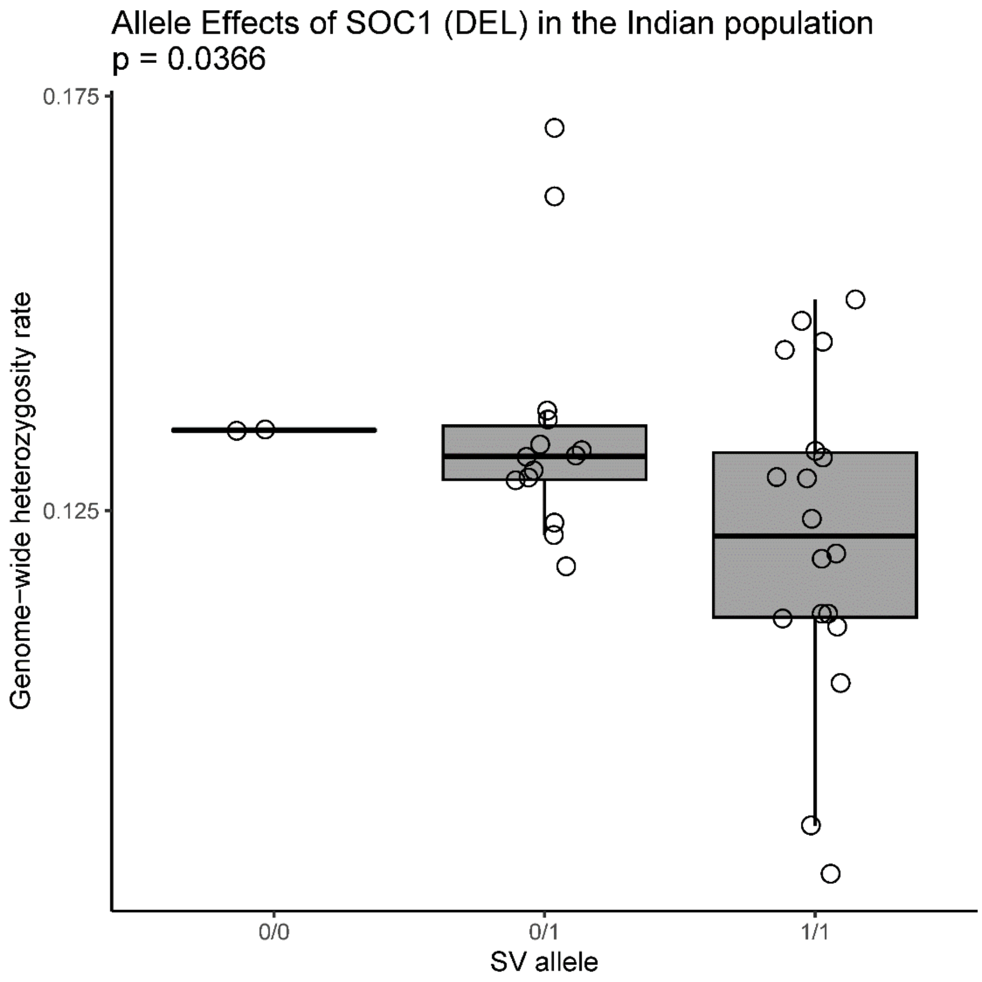


**Supplementary Fig. 11. Allele effects of the deletion variation that affect gene *SOC1* in the Indian population.**

Grey circles indicate the intra-individual heterozygosity rate. “0/0” refers to the homozygous reference allele, “0/1” refers to the heterozygous allele, and “1/1” refers to the homozygous alternative allele (allele that is impacted by the SV). P value was estimated from the genetic association using RVTESTS.

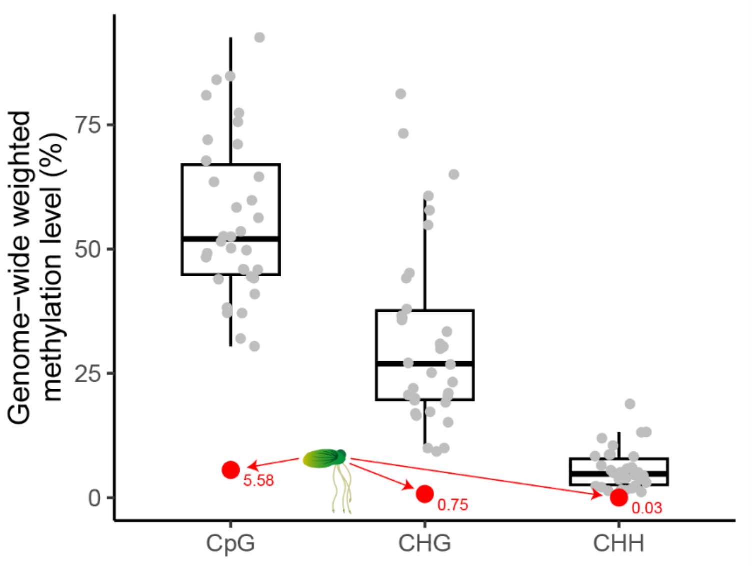


**Supplementary Fig. 12. Comparison of genome-wide methylation levels.**

The box plot compares the weighted methylation levels of 34 angiosperms and *S. polyrhiza* in CpG, CHG, and CHH. The red dots show the genome-wide weighted methylation levels of *S. polyrhiza*.

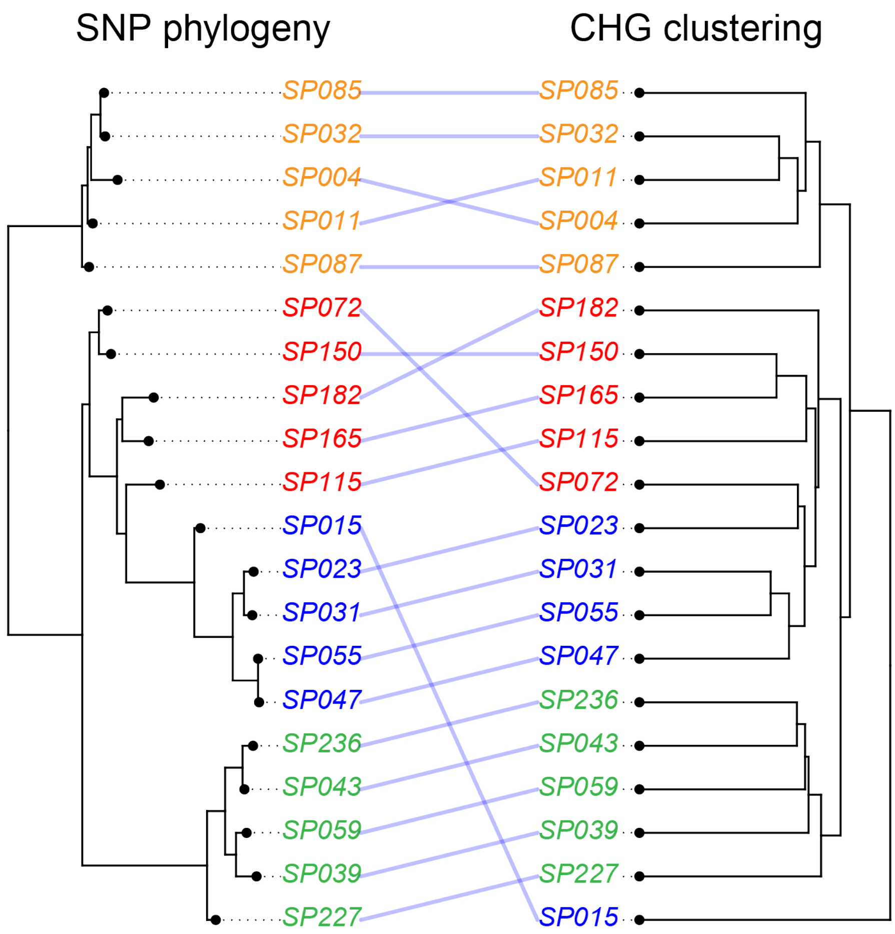


**Supplementary Fig. 13. Comparison between the genetic and CHG epigenetic distances among 20 individuals.**

The phylogeny was inferred from the SNP data. The hierarchical clustering was calculated based on the genome-wide gene body CHG methylation using “Ward methods”. Tips for both trees were differently colored based on their population classification.

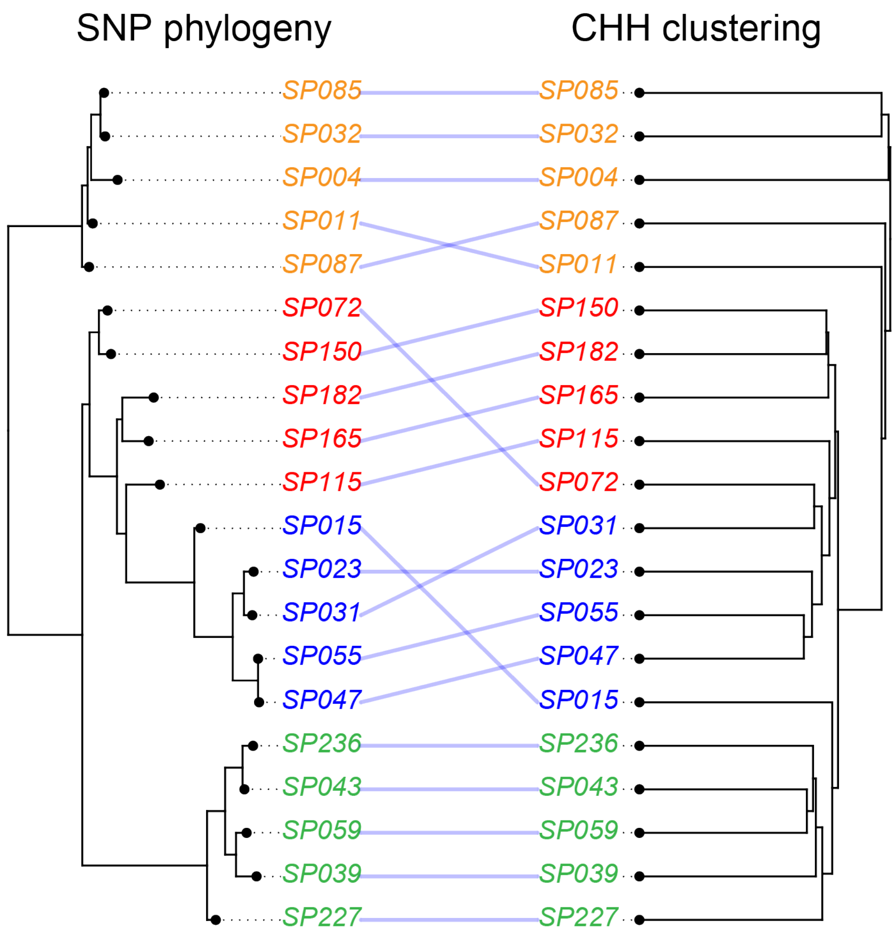


**Supplementary Fig. 14. Comparison between the genetic and CHH epigenetic distances among 20 individuals.**

The phylogeny was inferred from the SNP data. The hierarchical clustering was calculated based on the genome-wide gene body CHH methylation using "Ward methods". Tips for both trees were differently colored based on their population classification.

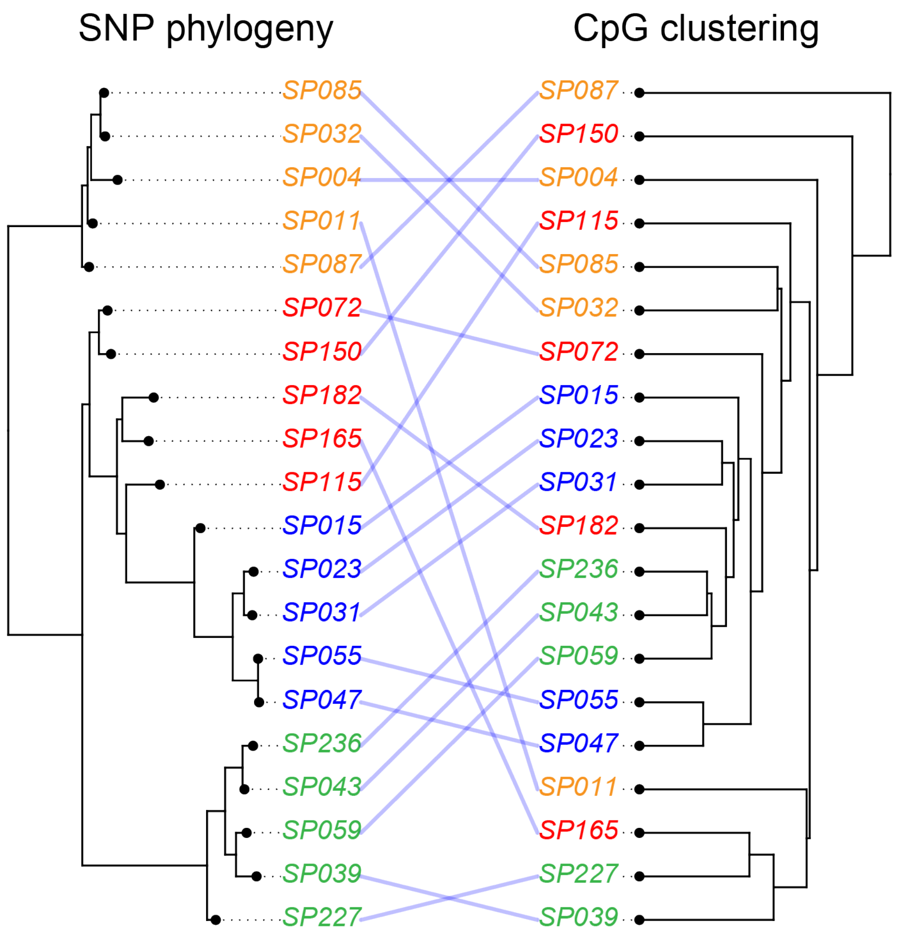


**Supplementary Fig. 15. Comparison between the genetic and CpG epigenetic distances among 20 individuals.**

The phylogeny was inferred from the SNP data. The hierarchical clustering was calculated based on the genome-wide gene body CpG methylation using "Ward methods". Tips for both trees were differently colored based on their population classification.

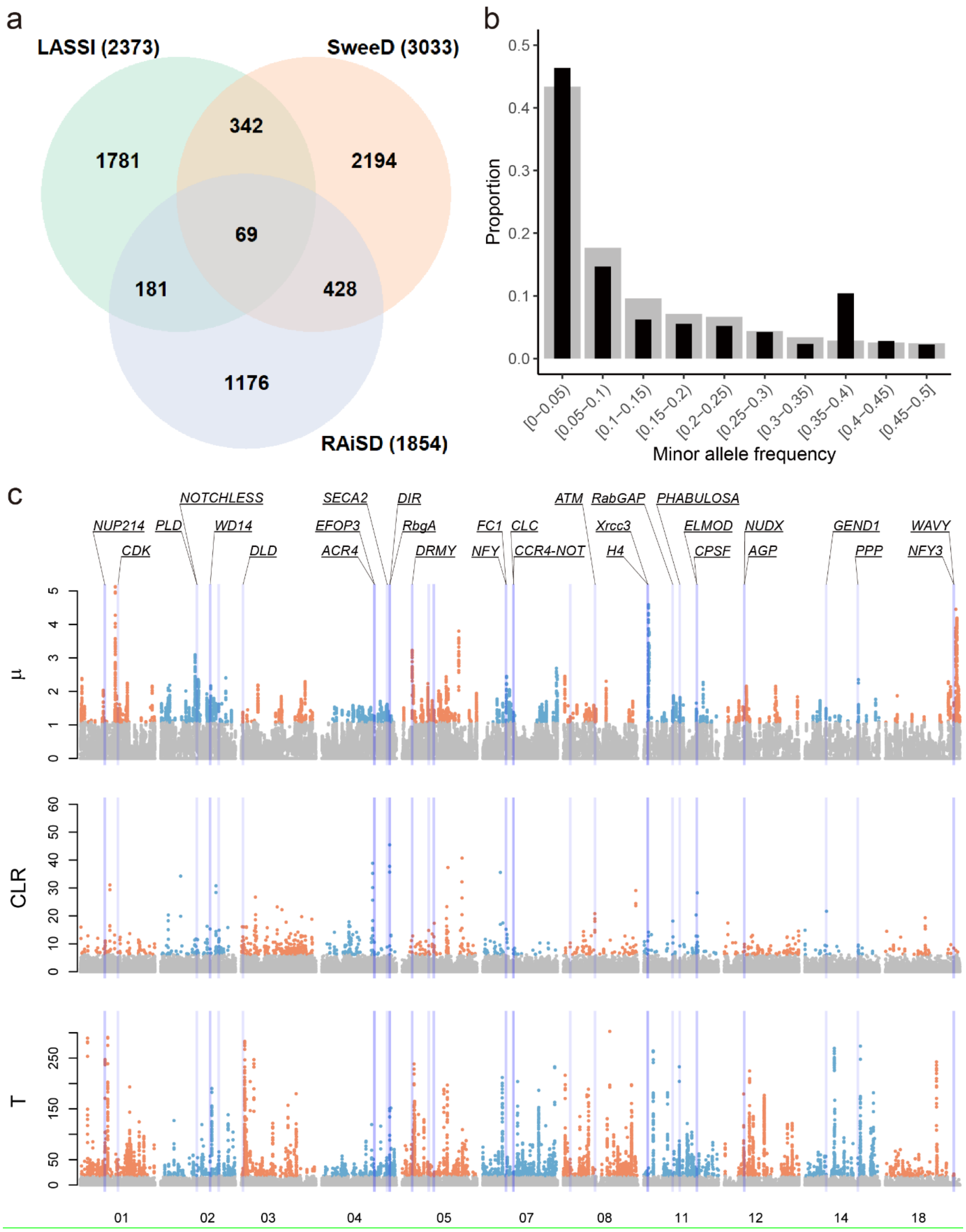


**Supplementary Fig. 16. Species-wide selection signature scans.**

**a** Number of genes under selection identified using three different methods. In total, 69 genes were found under selection by all three methods. **b** Folded site-frequency spectrum

(SFS) calculated from the 69 common genes (black bars) compared to the genome-wide folded SFS (grey bars). **c** Per-chromosome genome-scan results of all three methods, with indications of the genes under selection. Each dot represents a chromosomal locus reported by RAiSD (panel 1), SweeD (panel 2), or LASSI (panel 3) for which the statistic from each software was calculated ($\mu$ for RAiSD, CLR for SweeD, and T for LASSI). Significant outliers are shown in colored dots. Purple bars indicate the location of genes that are found to be under selection by all three methods.

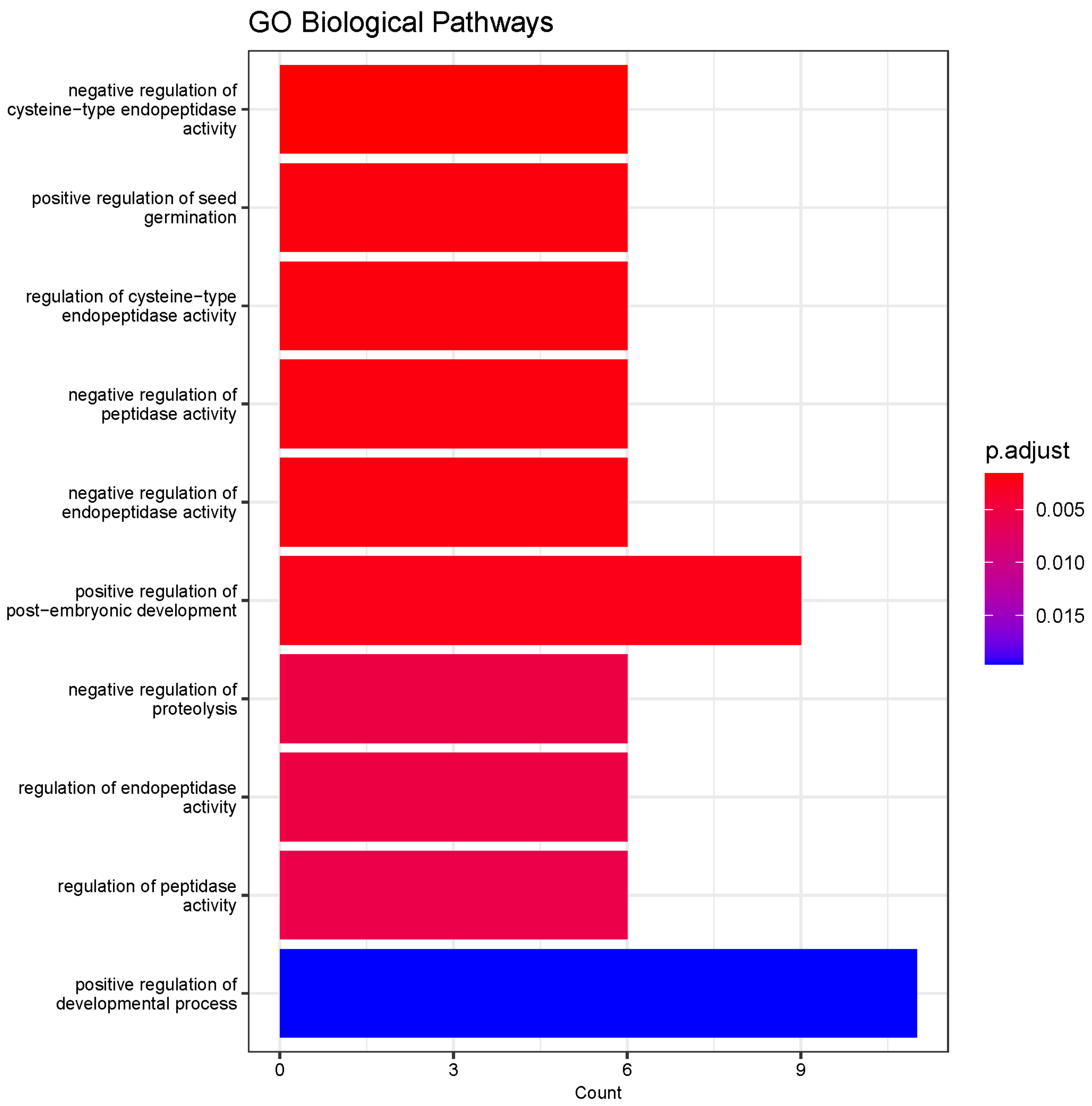


**Supplementary Fig. 17. GO enrichment of the genes found by the population-specific selection scan.**

X-axis refers to the number of genes, whereas Y-axis refers to individual GO terms. Colors indicate the adjusted *P*-values.

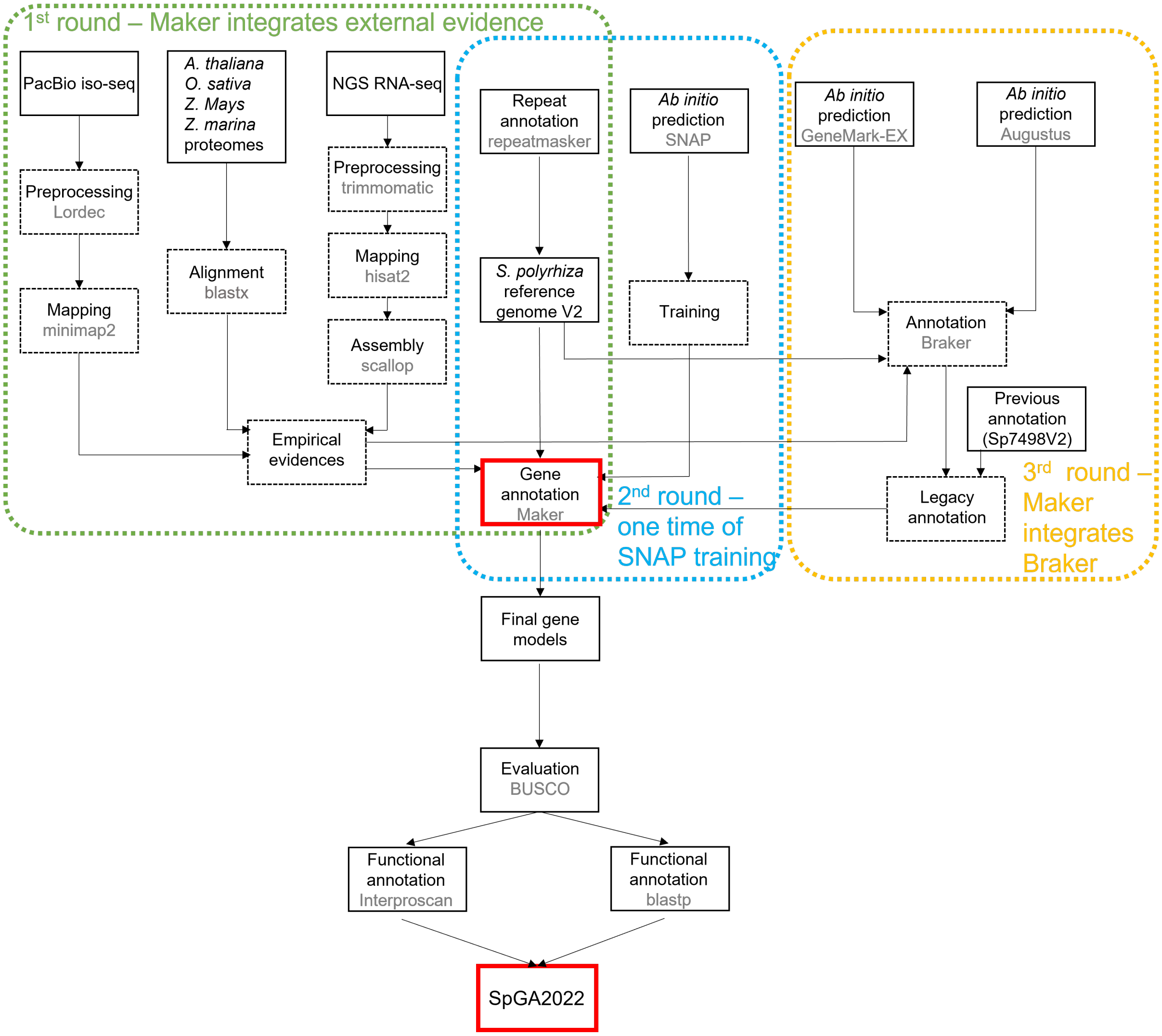


**Supplementary Fig. 18. The schematic illustration of the genome annotation pipeline.**

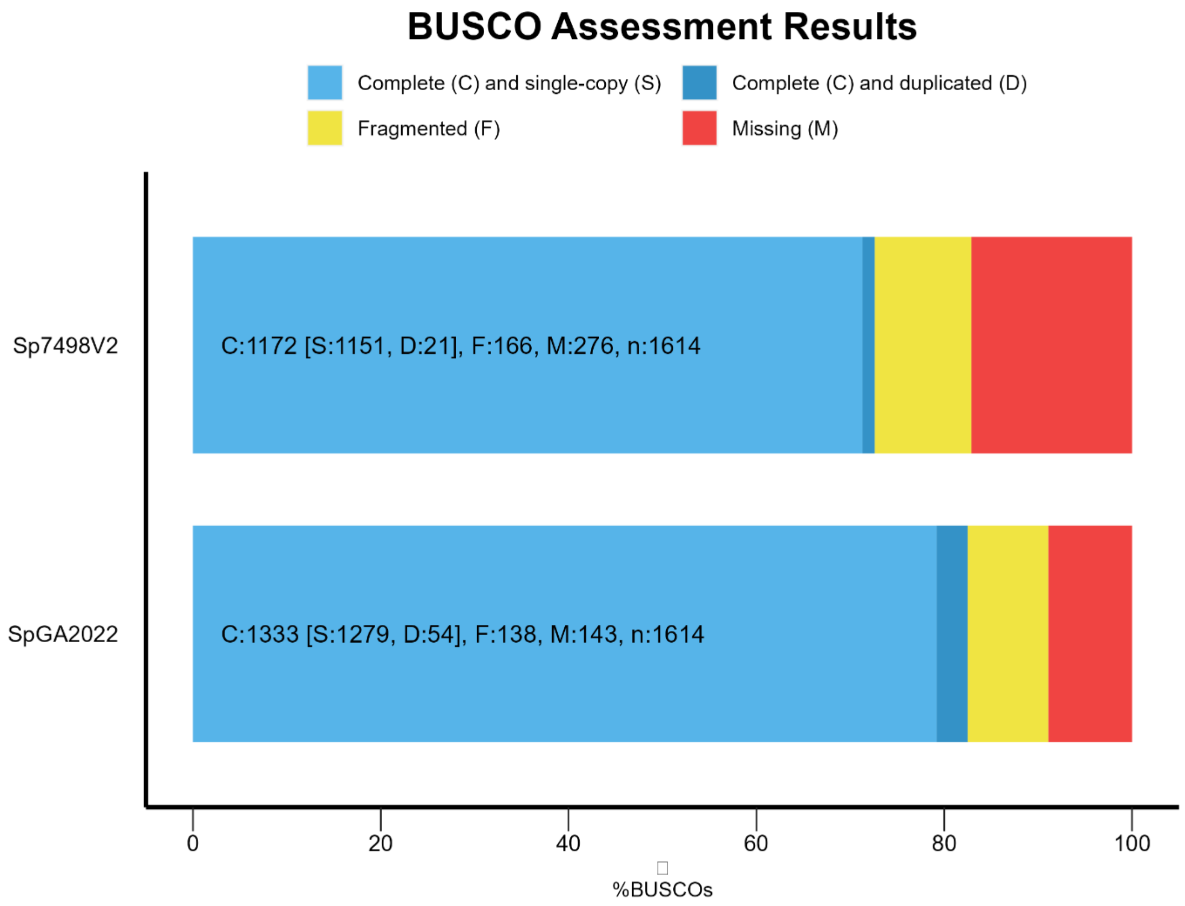


**Supplementary Fig. 19. The BUSCO comparison between SpGA2022 and Sp7498V2.**

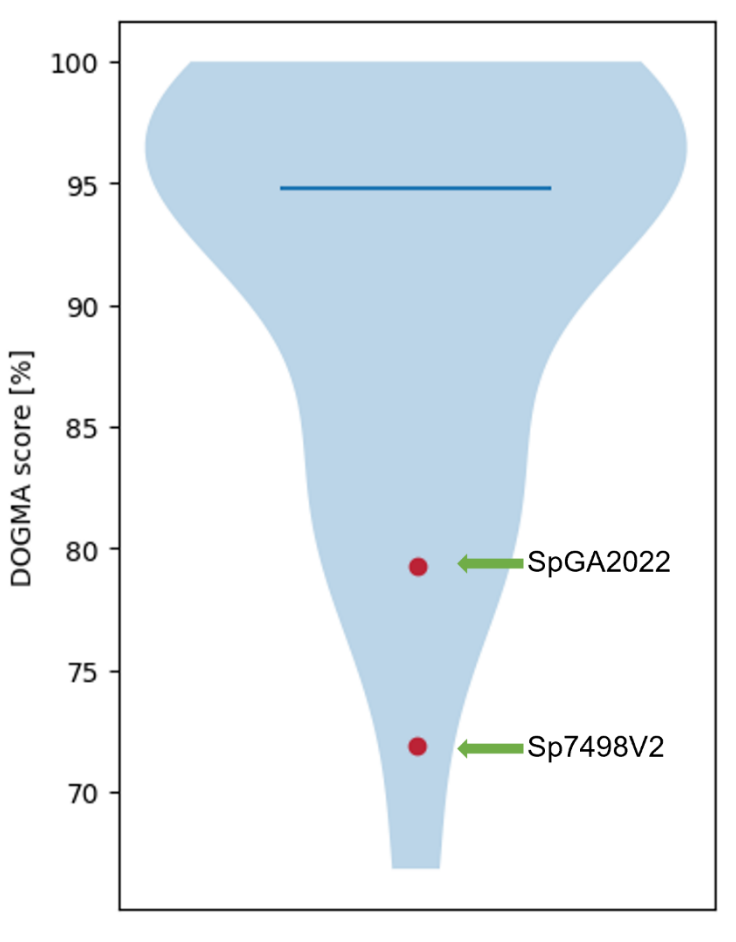


**Supplementary Fig. 20. The DOGMA comparison between SpGA2022 and Sp7498V2.**

The DOGMA scores of two annotations were shown in red points. The distribution of the other 23 monocot proteomes is shown in the blue area. The blue horizontal line indicates the median.

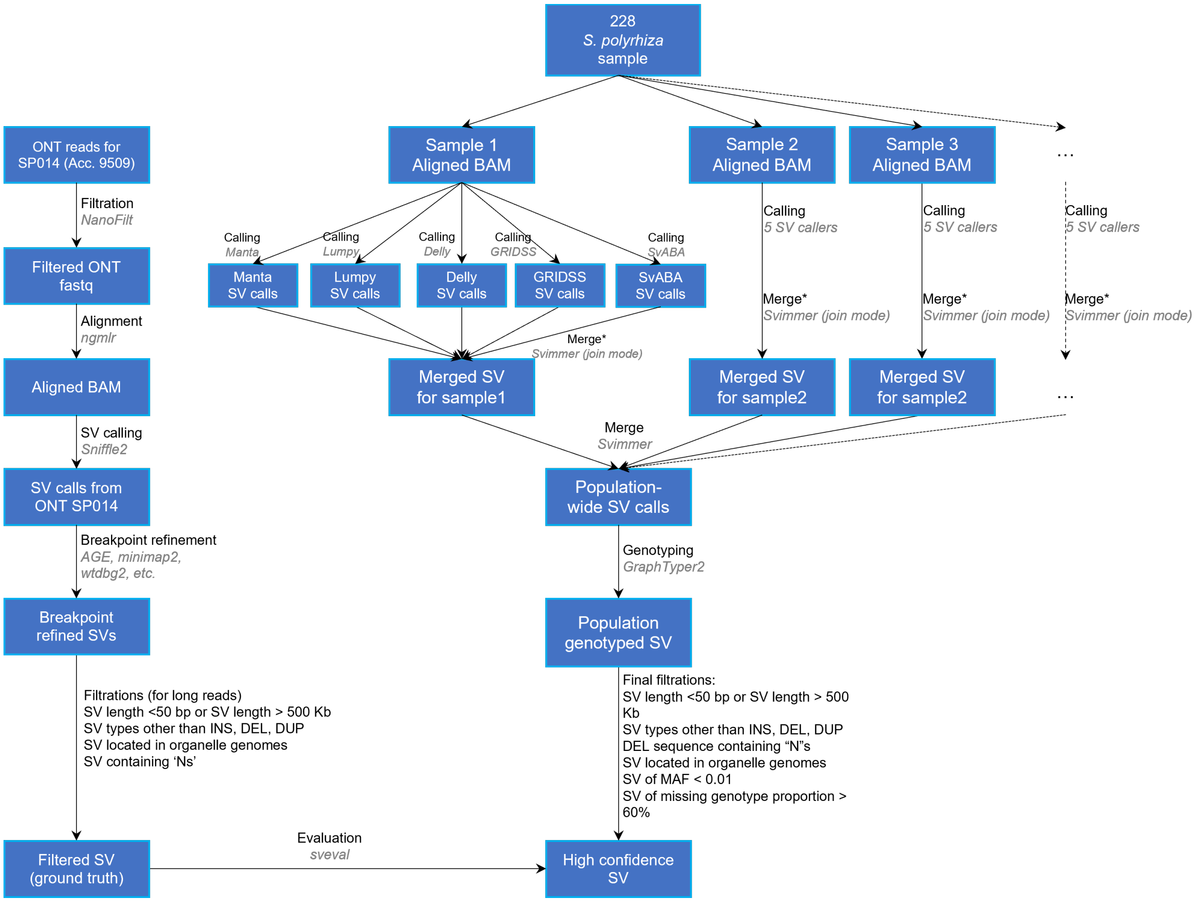


**Supplementary Fig. 21. Structural variation calling pipeline.**

Blue boxes indicate datasets or objects. Texts next to the arrows describe the processing steps. Tools used are shown in grey italic. *: merging SV calls from five different callers using Svimmer "join mode" means joining SV calls from Lumpy, Delly, GRIDSS, and SvABA with Manta.

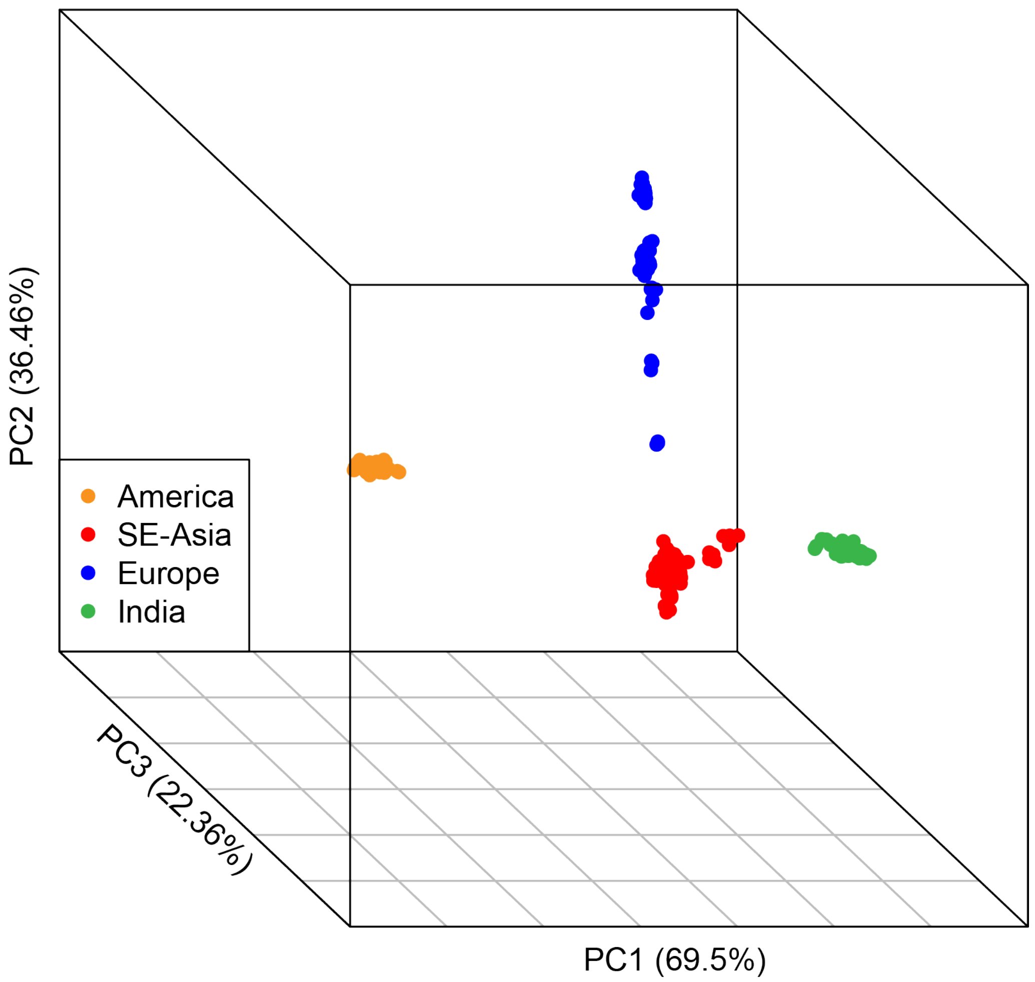


**Supplementary Fig. 22. Principal component analysis based on SVs.**

A three-dimensional plot shows the first three principal components. Small circular dots represent samples from the *S. polyrhiza* population. Different colours indicate the four populations that have been classified according to the population structure analysis.

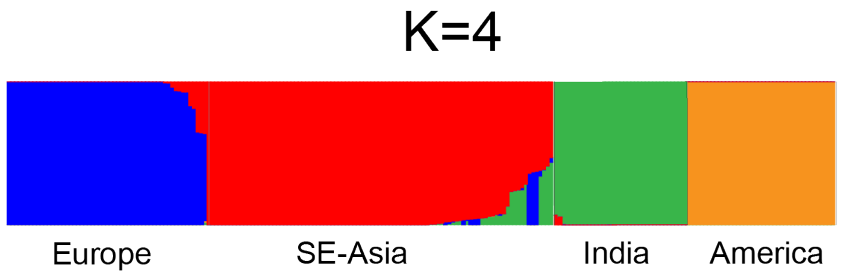


**Supplementary Fig. 23. Population structure analyses based on SVs.**

The plot shows the population structure analysis based on the SV data. The number of stratified groups was estimated as K=4 using fastStructure.

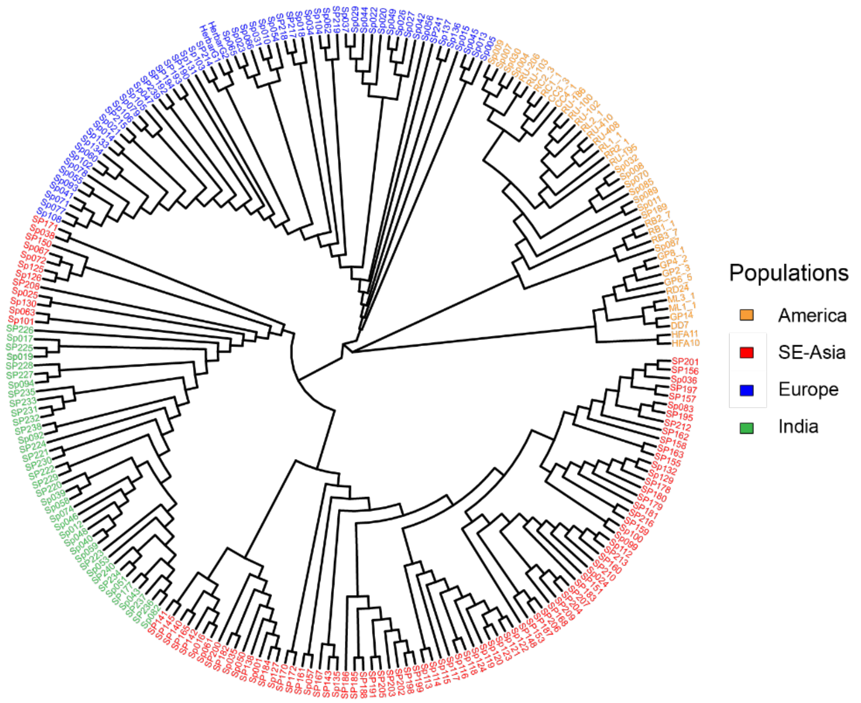


**Supplementary Fig. 24. Neighbour-Joining phylogenetic tree based on SVs.**

An unrooted neighbour-joining tree was reconstructed based on the p-distance calculated using population-level genotyped SVs. Tip names indicate sample names, while different colours represent the different populations to which the sample has been classified based on the population structure analysis: Asian (red), European (blue), Indian (green), and American (yellow).

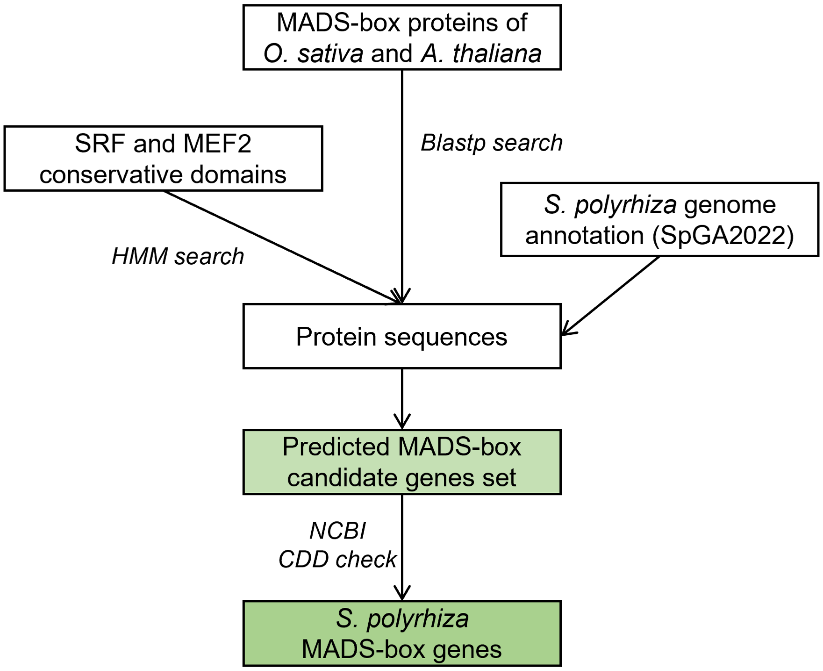


**Supplementary Fig. 25. The *S. polyrhiza* MADS-box annotation pipeline.**

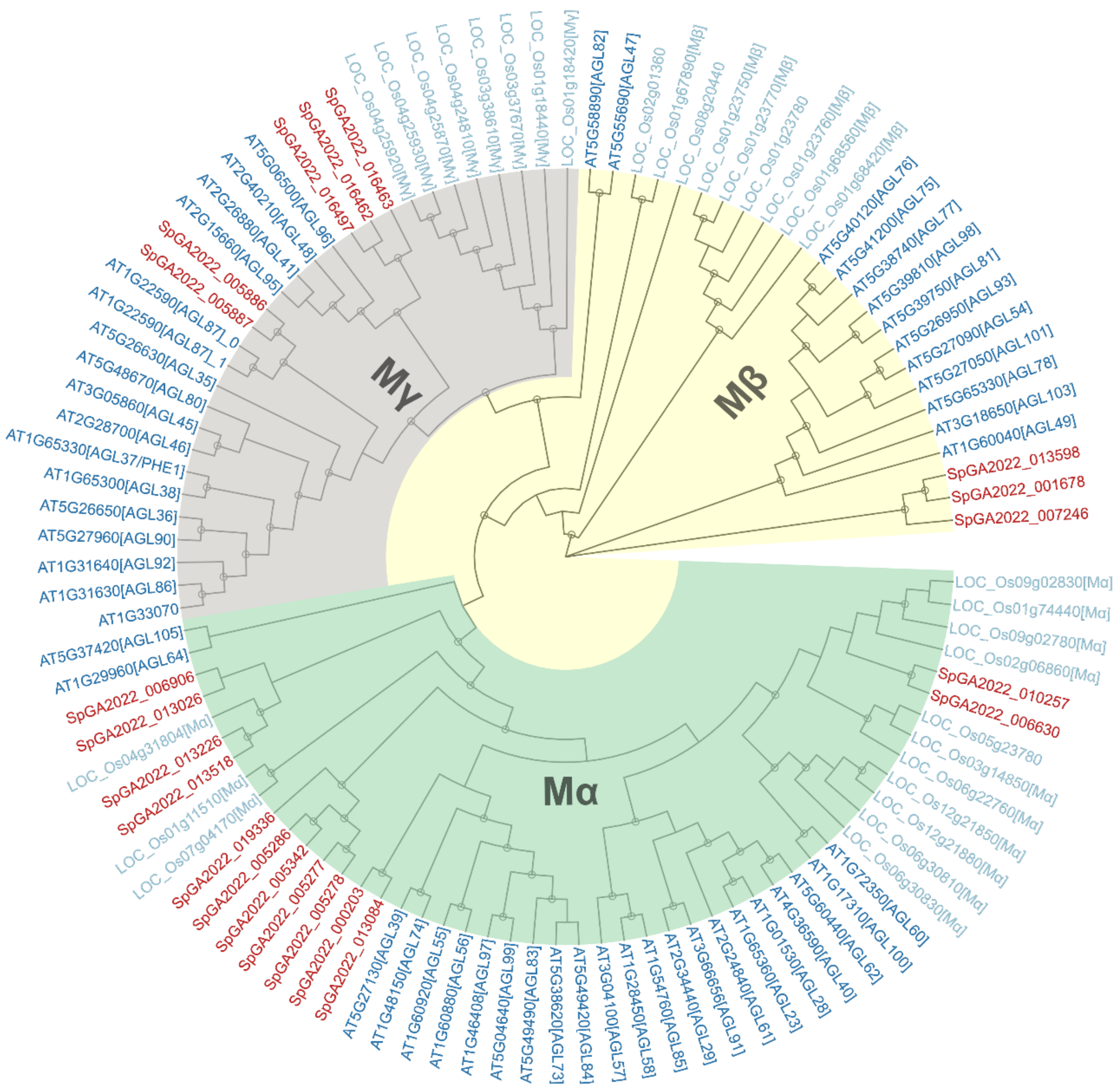


**Supplementary Fig. 26. Phylogeny of Type I MADS-box transcription factors.**

Phylogeny of Type I MADS-box transcription factor subfamily *in O. sativa*, *A. thaliana*, and *S. polyrhiza*. Three Type I MADS-box clades, Mα, Mβ, and Mγ, were highlighted using different colors. Tips are the gene names of *O. sativa* (starting with “LOC_Os” and indicated in light blue), *A. thaliana* (starting with “AT” and indicated in dark blue), and the *S. polyrhiza*’s updated gene annotation (SpGA2022, indicated in red). Genes from *O. sativa* and *A. thaliana* were partially annotated (information in square brackets) by previously classified clades based on public

databases or publications (see methods). Small circles on the internal nodes suggest bootstrapping support higher than 0.75 (max of 1.00).

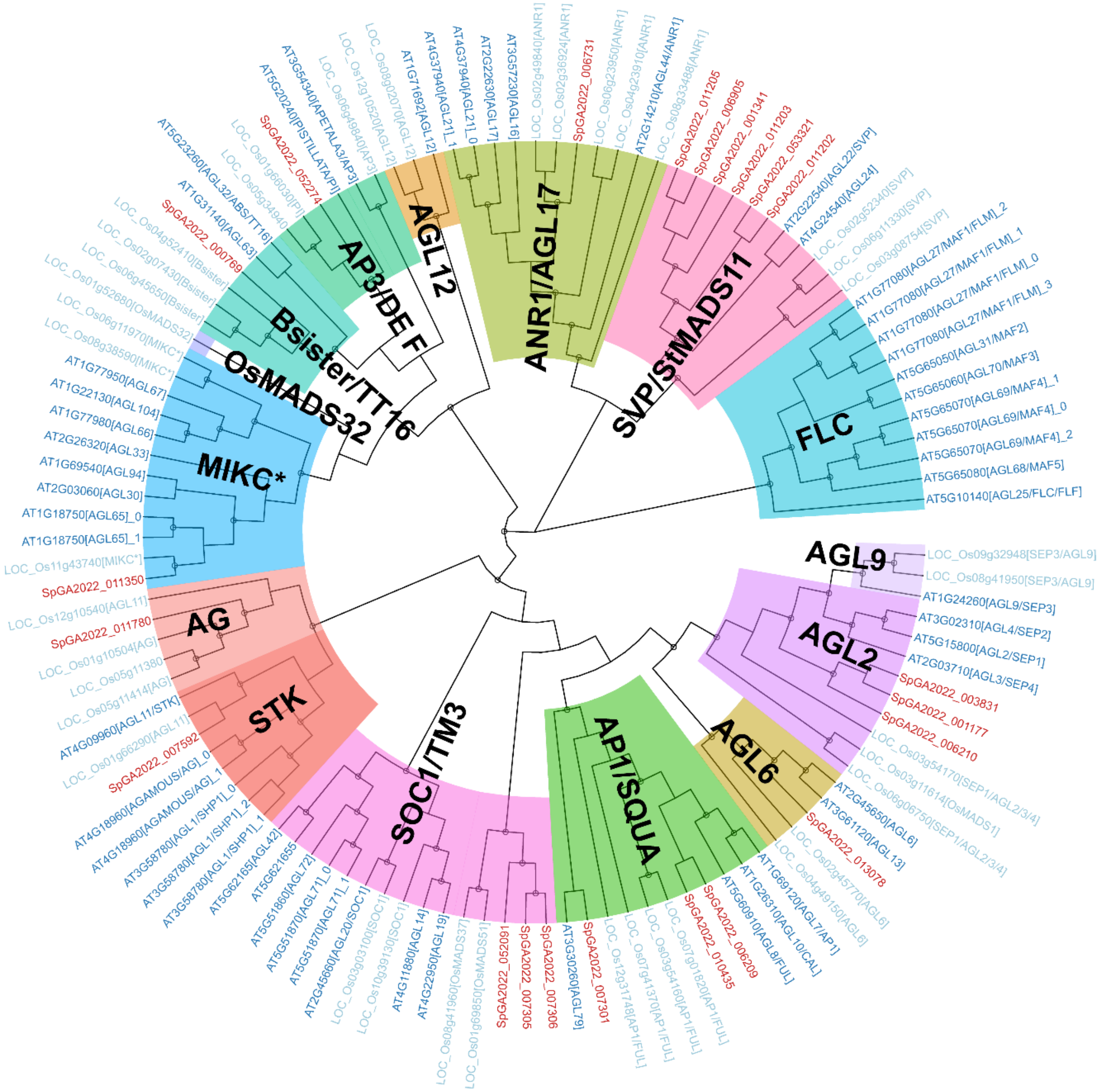


**Supplementary Fig. 27. Phylogeny of Type II MADS-box transcription factors.**

The phylogenetic relationship of 117 Type II MADS-box genes from three species was shown. The 15 subfamilies are marked in different colors.

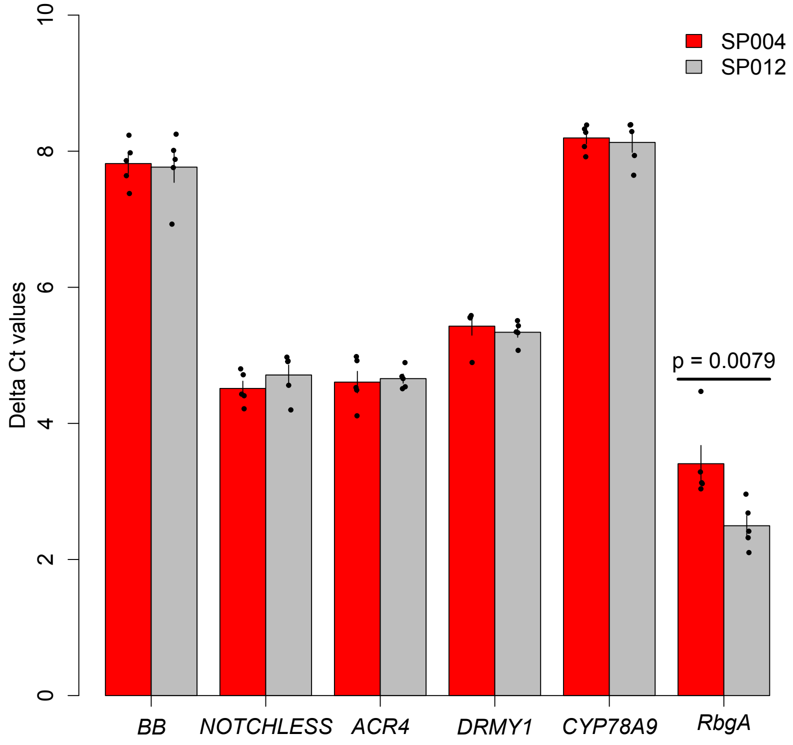


**Supplementary Fig. 28. Expression levels of candidate genes in two genotypes of SP004 and SP012**

The expression of six candidate genes was evaluated in the flowering genotype SP004 (red) and the one non-flowering genotype SP012 (grey). The Delta Ct values are based on the geometric mean of *GAPDH* and *aEF* expression. *RbgA* was differentially expressed between two tested genotypes (Wilcoxon rank sum test).**4. Supplementary Tables**

**Supplementary Table 1. Pairwise nucleotide diversity (π) among different populations.**

| Population | π | $\pi_N$* | $\pi_S$* | $\pi_N/\pi_S$ |
|---|---|---|---|---|
| America | 0.00054 | 0.00023 | 0.00056 | 0.41 |

| | | | | |
|---|---|---|---|---|
| SE-Asia | 0.00129 | 0.00046 | 0.00126 | 0.37 |
| Europe | 0.00081 | 0.00031 | 0.00084 | 0.37 |
| India | 0.00071 | 0.00028 | 0.00071 | 0.40 |
| Combined | 0.00160 | 0.00058 | 0.00155 | 0.37 |

***:** $\pi_N$ and $\pi_S$ refer to the pairwise nucleotide diversity at nonsynonymous and synonymous sites, respectively.

**Supplementary Table 2.** Pi per site for several important plant and animal species. Values here were included only for species with species-wide samples and whole-genome resequencing data is available.

| Species | Pi per site | Wild/cultivated | Reference |
|---|---|---|---|
| *Phaseolus vulgaris* | Between 0.0017 and 0.0050 | Domesticated | Schmutz *et al.*[68] |
| *Populus trichocarpa* | 0.0041 | Wild | Evans *et al.*[69] |
| *Citrullus lanatus* | 0.0076 | Domesticated | Guo *et al.*[70] |
| *Solanum lycopersicum* | 0.0073 | Domesticated | Lin *et al.*[71] |
| Malawi cyclids (mean across several species) | 0.0012 | Wild | Malinksy *et al.*[72] |
| *Prunus persica* | 0.0015 | Domesticated | The International Peach Genome Initiative[73] |
| *Triticum aestivum* | <0.0005 | Domesticated | Pont *et al.*[74] |
| *Drosophila melanogaster* | Between 0.0041 and 0.0070 | Wild | Pool *et al.*[75] |
| *Oryza sativa* | <0.005 | Domesticated | Wang *et al.*[76] |
| *Ovis aries* | Between 0.0010 and 0.0013 | Domesticated | Li *et al.*[77] |
| *Ficedula albicollis* | 0.00386 | Wild | Dutoit *et al.*[78] |

| | | | |
|---|---|---|---|
| *Arabidopsis thaliana* | 0.006 | Wild | The 1001 Genomes Consortium[79] |
| *Homo sapiens* | 0.001 | - | Jorde and Wooding[80] |
| *Vitis vinifera* | 0.0051 | Domesticated | Lijavetzky *et al.*[81] |
| *Spirodela polyrhiza* | 0.0016 | Wild | Present study |

**Supplementary Table 3. The annotation of SVs.**

| | Deletion | Duplication | Insertion |
|---|---|---|---|
| **3'_UTR_variant** | 99 | 80 | 22 |
| **5'_UTR_variant** | 87 | 75 | 15 |
| **coding_sequence_variant** | 169 | 155 | 34 |
| **downstream_gene_variant** | 579 | 79 | 323 |
| **frameshift_variant** | 37 | - | - |
| **inframe_deletion** | 20 | - | - |
| **intergenic_variant** | 604 | 49 | 196 |
| **intron_variant** | 919 | 209 | 303 |
| **start_codon_lost** | 2 | - | - |
| **start_codon_retained_variant** | 2 | - | - |
| **stop_lost** | 54 | - | - |
| **transcript_ablation** | 27 | - | - |
| **upstream_gene_variant** | 591 | 63 | 298 |

**Supplementary Table 4. Intergenic loci selected for demographic inference.**

| Chromosome | From | To | Size (bp) |
|---|---|---|---|
| ChrS01 | 10341926 | 10370644 | 28718 |
| ChrS01 | 6201042 | 6245822 | 44780 |
| ChrS02 | 4580335 | 4614088 | 33753 |
| ChrS02 | 7338329 | 7371076 | 32747 |
| ChrS03 | 2135139 | 2171492 | 36353 |
| ChrS03 | 3324230 | 3353948 | 29718 |
| ChrS04 | 2398564 | 2428164 | 29600 |
| ChrS04 | 5843951 | 5877409 | 33458 |
| ChrS04 | 8231251 | 8276638 | 45387 |
| ChrS05 | 5717537 | 5749587 | 32050 |
| ChrS05 | 5837287 | 5867849 | 30562 |
| ChrS06 | 2104286 | 2154345 | 50059 |
| ChrS06 | 2203817 | 2245642 | 41825 |
| ChrS07 | 4834724 | 4874602 | 39878 |
| ChrS09 | 1416036 | 1445139 | 29103 |
| ChrS09 | 3633841 | 3670635 | 36794 |
| ChrS09 | 3947117 | 3977500 | 30383 |
| ChrS10 | 6686084 | 6733993 | 47909 |
| ChrS11 | 2990169 | 3034837 | 44668 |
| ChrS11 | 5005366 | 5045753 | 40387 |
| ChrS12 | 2218087 | 2244224 | 26137 |
| ChrS12 | 457799 | 491403 | 33604 |
| ChrS13 | 1568966 | 1600801 | 31835 |
| ChrS13 | 5071361 | 5102204 | 30843 |
| ChrS14 | 1987237 | 2018224 | 30987 |
| ChrS14 | 340148 | 375438 | 35290 |
| ChrS14 | 4545337 | 4578899 | 33562 |
| ChrS15 | 1710444 | 1752107 | 41663 |
| ChrS15 | 1753520 | 1781319 | 27799 |
| ChrS15 | 3867569 | 3897413 | 29844 |
| ChrS16 | 1261209 | 1301988 | 40779 |
| ChrS16 | 3523583 | 3556154 | 32571 |
| ChrS16 | 4504899 | 4543897 | 38998 |
| ChrS17 | 2732045 | 2771792 | 39747 |
| ChrS18 | 2486433 | 2511566 | 25133 |
| ChrS18 | 664864 | 689461 | 24597 |
| ChrS19 | 2569311 | 2610161 | 40850 |
| ChrS20 | 1129392 | 1151473 | 22081 |
| ChrS20 | 715644 | 745921 | 30277 |

**Supplementary Table 5. Inferred demographic parameters.**

| Parameter | Prior | Posterior | 95% Confidence intervals |
|---|---|---|---|
| $\boldsymbol{Ne}_{\mathbf{Anc}}$ | $U(1x10^4,4x10^6)$ | 44875 ind. | (41853,48497) |
| $\boldsymbol{Ne}_{\mathbf{AME}}$ | $U(0.3,50)*Ne_{Anc}$ | $8.716*Ne_{Anc}$ (~400000 ind.) | (0.3,24.83) |
| $\boldsymbol{Ne}_{\mathbf{ASIA}}$ | $U(0.3,50)*Ne_{Anc}$ | $14.652*Ne_{Anc}$ (~660000 ind.) | (0.3,45.4) |
| $\boldsymbol{Ne}_{\mathbf{EUR}}$ | $U(0.3,3)*Ne_{Anc}$ | $1.657*Ne_{Anc}$ (~75000 ind.) | (0.83,2.43) |
| $\boldsymbol{Ne}_{\mathbf{IND}}$ | $U(0.3,3)*Ne_{Anc}$ | $3.000*Ne_{Anc}$ (~135000 ind.) | (0.3,3) |
| $\boldsymbol{T}_{\mathbf{AME}}$ | $U(T_{IND},30)*Ne_{Anc}$ | $22.021*Ne_{Anc}$ (~$1x10^6$ gen. ago) | (0.0001,27.43) |
| $\boldsymbol{T}_{\mathbf{ASIA}}$ | $U(T_{IND},30)*Ne_{Anc}$ | $22.454*Ne_{Anc}$ (~$1x10^6$ gen. ago) | (18.39,23.26) |
| $\boldsymbol{T}_{\mathbf{EUR}}$ | $U(0.0001,1)*Ne_{Anc}$ | $0.266*Ne_{Anc}$ (~12000 gen. ago) | (0.13,0.60) |
| $\boldsymbol{T}_{\mathbf{IND}}$ | $U(T_{EUR},30)*Ne_{Anc}$ | $1.120*Ne_{Anc}$ (~51000 gen. ago) | (0.0001,18.19) |
| $\boldsymbol{M}_{\mathbf{ASIA}}$ | $U(10^{-13},10^{-1})$ | $4Ne_{Anc}m$ = $7.4302x10^{-9}$ | $(10^{-11.9},10^{-6.1})$ |
| $\boldsymbol{M}_{\mathbf{EUR}}$ | $U(10^{-13},10^{-1})$ | $4Ne_{Anc}m$ = $1.3932x10^{-5}$ | $(10^{-9.41},10^{-3.28})$ |
| $\boldsymbol{M}_{\mathbf{IND}}$ | $U(10^{-13},10^{-1})$ | $4Ne_{Anc}m$ = $5.1286x10^{-13}$ | $(10^{-13},10^{-10.20})$ |

**Supplementary Table 6. The association of two MADS-box genes impacted SVs and the genome-wide heterozygosity rate within each population of *S. polyrhiza*.**

| SV | Chr | Position | Population | Beta | SE | P value |
|---|---|---|---|---|---|---|
| AGL62 (INS) | ChrS02 | 6841131 | SE-Asia | -0.0015 | 0.0046 | 0.745 |
| | | | India | -0.00038 | 0.005 | 0.938 |
| | | | Europe | 0.047 | 0.009 | **3.57e-07** |
| SOC1 (DEL) | ChrS04 | 5045470 | India | -0.0098 | 0.0047 | **0.037** |

**Supplementary Table 7. The list of 20 methylomes.**

| Clonal_family_ID | Sample_ID | Population |
|---|---|---|
| 9 | SP032 | America |
| 3 | SP087 | America |
| 11 | SP011 | America |
| 59 | SP085 | America |
| 5 | SP004 | America |
| 7 | SP047 | Europe |
| 10 | SP031 | Europe |
| 7 | SP055 | Europe |
| 39 | SP023 | Europe |
| 35 | SP015 | Europe |
| 145 | SP227 | India |
| 16 | SP039 | India |
| 47 | SP043 | India |
| 17 | SP059 | India |
| 154 | SP236 | India |
| 95 | SP165 | SE-Asia |
| 19 | SP072 | SE-Asia |
| 105 | SP182 | SE-Asia |
| 67 | SP115 | SE-Asia |
| 83 | SP150 | SE-Asia |

**Supplementary Table 8. The proportion of methylated cytosines among 20 *S. polyrhiza* methylomes.**

| Population | Sample | mC proportion# | | | |
|---|---|---|---|---|---|
| | | C | CpG | CHG | CHH |
| America | SP011 | 2.01% | 9.37% | 3.51% | 0.13% |
| | SP032 | 1.89% | 9.18% | 3.10% | 0.11% |
| | SP004 | 1.57% | 7.65% | 2.08% | 0.05% |
| | SP085 | 1.34% | 6.45% | 2.04% | 0.08% |
| | SP087 | 1.40% | 6.67% | 1.93% | 0.09% |
| SE-Asia | SP115 | 1.64% | 7.53% | 2.72% | 0.13% |
| | SP150 | 1.82% | 8.11% | 2.40% | 0.17% |
| | SP165 | 2.28% | 11.12% | 4.32% | 0.23% |
| | SP182 | 1.38% | 5.74% | 2.04% | 0.16% |
| | SP072 | 1.46% | 6.73% | 2.07% | 0.14% |
| Europe | SP015 | 1.27% | 6.82% | 0.94% | 0.11% |
| | SP023 | 1.44% | 6.77% | 2.12% | 0.09% |
| | SP031 | 1.88% | 8.93% | 3.60% | 0.15% |
| | SP047 | 1.49% | 7.08% | 2.10% | 0.09% |
| | SP055 | 1.63% | 7.48% | 2.83% | 0.10% |
| India | SP227 | 1.53% | 7.62% | 1.74% | 0.10% |
| | SP236 | 1.42% | 6.94% | 1.81% | 0.09% |
| | SP039 | 1.51% | 7.74% | 1.72% | 0.07% |
| | SP043 | 1.38% | 6.60% | 1.78% | 0.09% |
| | SP059 | 1.41% | 6.77% | 1.97% | 0.09% |

#mC proportion: The fraction of methylated cytosines in all sequencing-covered cytosines.

**Supplementary Table 9. Comparison between annotations of SpGA2022 and Sp7498V2.**

| | SpGA2022 | Sp7498V2 |
|---|---|---|
| # of genes | 20,546 | 19,620 |
| # of genes with UTR on both sides | 7,937 | - |
| # of genes with at least one UTR | 9,500 | - |
| # of single exon gene | 2,191 | 3,806 |
| Total CDS length | 23.6 Mb | 21.7 Mb |
| Total exon length | 30.5 Mb | 21.7 Mb |
| mean exons per mRNA | 5.5 | 5.2 |
| mean gene length | 4,225 | 3,455 |
| mean CDS length | 1,110 | 1,107 |
| mean exon length | 261 | 212 |
| mean five_prime_UTR length | 380 | - |
| mean three_prime_UTR length | 414 | - |
| # of mRNA without a start codon | 191 | 1,222 |
| # of mRNA without a stop codon | 470 | 508 |
| # of mRNA without both start and stop codons | 63 | 2,668 |

**Supplementary Table 10. MADS-box transcription factors identification in *S. polyrhiza*.**

| SUB-FAMILIES | GENE_ID | CLADES |
|---|---|---|
| TypeI | SpGA2022_006630 | Mα |
| TypeI | SpGA2022_010257 | Mα |
| TypeI | SpGA2022_000203 | Mα |
| TypeI | SpGA2022_013084 | Mα |
| TypeI | SpGA2022_005286 | Mα |
| TypeI | SpGA2022_005342 | Mα |
| TypeI | SpGA2022_005277 | Mα |
| TypeI | SpGA2022_005278 | Mα |
| TypeI | SpGA2022_019336 | Mα |
| TypeI | SpGA2022_013226 | Mα |
| TypeI | SpGA2022_013518 | Mα |
| TypeI | SpGA2022_006906 | Mα |
| TypeI | SpGA2022_013026 | Mα |
| TypeI | SpGA2022_001678 | Mβ |
| TypeI | SpGA2022_013598 | Mβ |
| TypeI | SpGA2022_007246 | Mβ |
| TypeI | SpGA2022_005886 | Mγ |
| TypeI | SpGA2022_005887 | Mγ |
| TypeI | SpGA2022_016462 | Mγ |
| TypeI | SpGA2022_016497 | Mγ |
| TypeI | SpGA2022_016463 | Mγ |
| TypeII | SpGA2022_052274 | AP3/DEF |
| TypeII | SpGA2022_000769 | Bsister/TT16 |
| TypeII | SpGA2022_006731 | ANR1/AGL17 |
| TypeII | SpGA2022_001341 | SVP/StMADS11 |
| TypeII | SpGA2022_011205 | SVP/StMADS11 |
| TypeII | SpGA2022_011203 | SVP/StMADS11 |
| TypeII | SpGA2022_053321 | SVP/StMADS11 |
| TypeII | SpGA2022_011202 | SVP/StMADS11 |
| TypeII | SpGA2022_006905 | SVP/StMADS11 |
| TypeII | SpGA2022_011350 | MIKC* |
| TypeII | SpGA2022_007592 | AG/STK |
| TypeII | SpGA2022_011780 | AG/STK |
| TypeII | SpGA2022_052091 | SOC1/TM3 |
| TypeII | SpGA2022_007306 | SOC1/TM3 |
| TypeII | SpGA2022_007305 | SOC1/TM3 |
| TypeII | SpGA2022_010435 | AP1/SQUA |
| TypeII | SpGA2022_006209 | AP1/SQUA |
| TypeII | SpGA2022_007301 | AP1/SQUA |
| TypeII | SpGA2022_013078 | AGL6 |
| TypeII | SpGA2022_001177 | SEP/AGL2 |
| TypeII | SpGA2022_003831 | SEP/AGL2 |

| | | |
|---|---|---|
| TypeII | SpGA2022_006210 | SEP/AGL2 |

**Supplementary Table 11. Primers used for RT-qPCR gene expression study**

| Gene | Forward primer 5'-3' | Reverse primer 5'-3' | Product size | Primer efficiency |
|---|---|---|---|---|
| *GAPDH* | AGCATCCAAGAAG GTGAAGATCGGC | TTGTAGTCGGTCG TGATGAAGGGG | 132 bp | 104.4 % |
| *aEF* | TCGAAGCCGGCATT TCCAAGGACG | TCGCCTTCGAGTA CTTGGGTGTCG | 129 bp | 99.8 % |
| *BB* | CTCACCACAATGGC ACCAATCCTC | CTTGTACTTTGAG ACTGGGAGCGAC | 161 bp | 84.7 % |
| *NOTCHLESS* | CTGGCAGTAACTTG TGTGAGGTGG | TGCGGAGTGTGTA TTCGGTGCTC | 169 bp | 99.9 % |
| *ACR4* | TTGCCCTCCTCGTA CTGCATGTC | TCTCCTGGGACCT GCGGATAG | 189 bp | 92.2 % |
| *DRMY1* | GCCATCAAGATGC CTATCATTGGG | TTGTGAAGTTGCT GGGTTGCTGG | 186 bp | 92.0 % |
| *CYP78A9* | GCCGTATCATCAAG CAACACCGC | CCCCTCGAAATAT CATCTCCCAGAG | 147 bp | 90.6 % |
| *RbgA* | CTGATGTGAATGTC AAGCGTAGAGC | TTCAACTCTCTTG TGACACCTGGC | 163 bp | 98.4 % |

**Supplementary Table 12. The expression validation of 8 candidate genes using qRT-PCR and RNA-seq**

| Gene | Orthologs in *A. thaliana* | qRT-PCR | RNA-seq |
|---|---|---|---|
| **SpGA2022_005278** | *AGL62* | Not expressed | No expressed |
| **SpGA2022_006111** | *BB* | expressed | Expressed |
| **SpGA2022_013078** | *AGL6* | Not expressed | No expressed |
| **SpGA2022_005107** | *NOTCHLESS* | expressed | Expressed |
| **SpGA2022_052159** | *ACR4* | expressed | No expressed |
| **SpGA2022_052378** | *DRMY1* | expressed | Expressed |
| **SpGA2022_055195** | *CYP78A9* | expressed | Expressed |
| **SpGA2022_007853** | *RgbA* | expressed | Expressed |

## 5. Supplementary Reference


1 An, D. *et al.* Plant evolution and environmental adaptation unveiled by long-read whole-genome sequencing of *Spirodela*. *Proc. Natl. Acad. Sci. USA* **116**, 18893-18899 (2019).

2 Jiang, H. S., Lei, R., Ding, S. W. & Zhu, S. F. Skewer: a fast and accurate adapter trimmer for next-generation sequencing paired-end reads. *BMC Bioinformatics* **15** (2014).

3 Kim, D., Paggi, J. M., Park, C., Bennett, C. & Salzberg, S. L. Graph-based genome alignment and genotyping with HISAT2 and HISAT-genotype. *Nat. Biotechnol.* **37**, 907-+ (2019).

4 Li, H. *et al.* The Sequence Alignment/Map format and SAMtools. *Bioinformatics* **25**, 2078-2079 (2009).

5 Shao, M. F. & Kingsford, C. Accurate assembly of transcripts through phase-preserving graph decomposition. *Nat. Biotechnol.* **35**, 1167-+ (2017).

6 Li, H. Minimap2: pairwise alignment for nucleotide sequences. *Bioinformatics* **34**, 3094-3100 (2018).

7 Ouyang, S. *et al.* The TIGR Rice Genome Annotation Resource: Improvements and new features. *Nucleic Acids Res.* **35**, D883-D887 (2007).

8 Portwood, J. L. *et al.* MaizeGDB 2018: the maize multi-genome genetics and genomics database. *Nucleic Acids Res.* **47**, D1146-D1154 (2019).

9 Lamesch, P. *et al.* The Arabidopsis Information Resource (TAIR): improved gene annotation and new tools. *Nucleic Acids Res.* **40**, D1202-D1210 (2012).

10 Sterck, L., Billiau, K., Abeel, T., Rouze, P. & van de Peer, Y. ORCAE: online resource for community annotation of eukaryotes. *Nat. Methods* **9**, 1041-1041 (2012).

11 Holt, C. & Yandell, M. MAKER2: an annotation pipeline and genome-database management tool for second-generation genome projects. *BMC Bioinformatics* **12** (2011).

12 Bruna, T., Hoff, K. J., Lomsadze, A., Stanke, M. & Borodovsky, M. BRAKER2: automatic eukaryotic genome annotation with GeneMark-EP plus and AUGUSTUS supported by a protein database. *NAR Genom. Bioinform.* **3** (2021).

13 Boratyn, G. M. *et al.* BLAST: a more efficient report with usability improvements. *Nucleic Acids Res.* **41**, W29-W33 (2013).

14 Quevillon, E. *et al.* InterProScan: protein domains identifier. *Nucleic Acids Res.* **33**, W116-W120 (2005).

15 Hernández-Salmerón, J. E. & Moreno-Hagelsieb, G. Progress in quickly finding orthologs as reciprocal best hits: comparing blast, last, diamond and MMseqs2. *BMC Genomics* **21** (2020).

16 Eggertsson, H. P. *et al.* GraphTyper2 enables population-scale genotyping of structural variation using pangenome graphs. *Nat. Commun.* **10** (2019).

17 Chen, X. Y. *et al.* Manta: rapid detection of structural variants and indels for germline and cancer sequencing applications. *Bioinformatics* **32**, 1220-1222 (2016).

18 Layer, R. M., Chiang, C., Quinlan, A. R. & Hall, I. M. LUMPY: a probabilistic framework for structural variant discovery. *Genome Biol.* **15** (2014).

19 Chiang, C. *et al.* SpeedSeq: ultra-fast personal genome analysis and interpretation. *Nat. Methods* **12**, 966-968 (2015).

20 Cameron, D. L. *et al.* GRIDSS: sensitive and specific genomic rearrangement detection using positional de Bruijn graph assembly. *Genome Res.* **27**, 2050-2060 (2017).

21 Rausch, T. *et al.* DELLY: structural variant discovery by integrated paired-end and split-read analysis. *Bioinformatics* **28**, i333-i339 (2012).

22 Wala, J. A. *et al.* SvABA: genome-wide detection of structural variants and indels by local assembly. *Genome Res.* **28**, 581-591 (2018).

23 Danecek, P. *et al.* Twelve years of SAMtools and BCFtools. *Gigascience* **10** (2021).

24 Li, H. Tabix: fast retrieval of sequence features from generic TAB-delimited files. *Bioinformatics* **27**, 718-719 (2011).

25 Danecek, P. *et al.* The variant call format and VCFtools. *Bioinformatics* **27**, 2156-2158 (2011).

26 Rice, P., Longden, I. & Bleasby, A. EMBOSS: The European molecular biology open software suite. *Trends Genet.* **16**, 276-277 (2000).

27 Yu, G. C., Smith, D. K., Zhu, H. C., Guan, Y. & Lam, T. T. Y. GGTREE: an R package for visualization and annotation of phylogenetic trees with their covariates and other associated data. *Methods Ecol. Evol.* **8**, 28-36 (2017).

28 Belyeu, J. R. *et al.* Samplot: a platform for structural variant visual validation and automated filtering. *Genome Biol.* **22** (2021).

29 Buels, R. *et al.* JBrowse: a dynamic web platform for genome visualization and analysis. *Genome Biol.* **17** (2016).

30 Kawahara, Y. *et al.* Improvement of the *Oryza sativa* Nipponbare reference genome using next generation sequence and optical map data. *Rice* **6** (2013).

31 Eddy, S. R. Accelerated Profile HMM Searches. *PLoS Comp. Biol.* **7** (2011).

32 Marchler-Bauer, A. *et al.* CDD: NCBI's conserved domain database. *Nucleic Acids Res.* **43**, D222-D226 (2015).

33 Parenicova, L. *et al.* Molecular and phylogenetic analyses of the complete MADS-box transcription factor family in *Arabidopsis*: New openings to the MADS world. *Plant Cell* **15**, 1538-1551 (2003).

34 Arora, R. *et al.* MADS-box gene family in rice: genome-wide identification, organization and expression profiling during reproductive development and stress. *BMC Genomics* **8** (2007).

35 Hu, J. *et al.* The pineapple MADS-box gene family and the evolution of early monocot flower. *Sci. Rep.* **11** (2021).

36 Fatima, M. *et al.* Expression profiling of MADS-box gene family revealed its role in vegetative development and stem ripening in *S. spontaneum*. *Sci. Rep.* **10** (2020).

37 Lin, Z. Y., Cao, D. D., Damaris, R. N. & Yang, P. F. Genome-wide identification of MADS-box gene family in sacred lotus (*Nelumbo nucifera*) identifies a SEPALLATA homolog gene involved in floral development. *BMC Plant Biol.* **20** (2020).

38 Kuraku, S., Zmasek, C. M., Nishimura, O. & Katoh, K. aLeaves facilitates on-demand exploration of metazoan gene family trees on MAFFT sequence alignment server with enhanced interactivity. *Nucleic Acids Res.* **41**, W22-W28 (2013).

39 Price, M. N., Dehal, P. S. & Arkin, A. P. FastTree: Computing Large Minimum Evolution Trees with Profiles instead of a Distance Matrix. *Mol. Biol. Evol.* **26**, 1641-1650 (2009).

40 Emms, D. M. & Kelly, S. OrthoFinder: phylogenetic orthology inference for comparative genomics. *Genome Biol.* **20** (2019).

41 Berardini, T. Z. *et al.* The Arabidopsis information resource: Making and mining the "gold standard" annotated reference plant genome. *Genesis* **53**, 474-485 (2015).

42 Gel, B. *et al.* regioneR: an R/Bioconductor package for the association analysis of genomic regions based on permutation tests. *Bioinformatics* **32**, 289-291 (2016).

43 Wakeley, J. & Hey, J. Estimating ancestral population parameters. *Genetics* **145**, 847-855 (1997).

44 Ewing, G. & Hermisson, J. MSMS: a coalescent simulation program including recombination, demographic structure and selection at a single locus. *Bioinformatics* **26**, 2064-2065 (2010).

45 Fagundes, N. J. R. *et al.* Statistical evaluation of alternative models of human evolution. *Proc. Natl. Acad. Sci. USA* **104**, 17614-17619 (2007).

46 Tavare, S., Balding, D. J., Griffiths, R. C. & Donnelly, P. Inferring coalescence times from DNA sequence data. *Genetics* **145**, 505-518 (1997).

47 Pritchard, J. K., Seielstad, M. T., Perez-Lezaun, A. & Feldman, M. W. Population growth of human Y chromosomes: A study of Y chromosome microsatellites. *Mol. Biol. Evol.* **16**, 1791-1798 (1999).

48 Beaumont, M. A., Zhang, W. Y. & Balding, D. J. Approximate Bayesian computation in population genetics. *Genetics* **162**, 2025-2035 (2002).

49 Wegmann, D., Leuenberger, C. & Excoffier, L. Efficient Approximate Bayesian Computation Coupled With Markov Chain Monte Carlo Without Likelihood. *Genetics* **182**, 1207-1218 (2009).

50 Csillery, K., Francois, O. & Blum, M. G. B. abc: an R package for approximate Bayesian computation (ABC). *Methods Ecol. Evol.* **3**, 475-479 (2012).

51 Akalin, A. *et al.* methylKit: a comprehensive R package for the analysis of genome-wide DNA methylation profiles. *Genome Biol.* **13** (2012).

52 Bolger, A. M., Lohse, M. & Usadel, B. Trimmomatic: a flexible trimmer for Illumina sequence data. *Bioinformatics* **30**, 2114-2120 (2014).

53 Liao, Y., Smyth, G. K. & Shi, W. featureCounts: an efficient general purpose program for assigning sequence reads to genomic features. *Bioinformatics* **30**, 923-930 (2014).

54 Love, M. I., Huber, W. & Anders, S. Moderated estimation of fold change and dispersion for RNA-seq data with DESeq2. *Genome Biol* **15**, 550 (2014).

55 Hellemans, J., Mortier, G., De Paepe, A., Speleman, F. & Vandesompele, J. qBase relative quantification framework and software for management and automated analysis of real-time quantitative PCR data. *Genome Biol* **8**, R19 (2007).

56 Kemena, C., Dohmen, E. & Bornberg-Bauer, E. DOGMA: a web server for proteome and transcriptome quality assessment. *Nucleic Acids Res.* **47**, W507-W510 (2019).

57 Hoang, P. N. T. *et al.* Generating a high-confidence reference genome map of the Greater Duckweed by integration of cytogenomic, optical mapping, and Oxford Nanopore technologies. *Plant J.* **96**, 670-684 (2018).

58 Lemay, M. A. *et al.* Combined use of Oxford Nanopore and Illumina sequencing yields insights into soybean structural variation biology. *BMC Biol.* **20** (2022).

59 Zhou, Y. F. *et al.* The population genetics of structural variants in grapevine domestication. *Nat. Plants* **5**, 965-979 (2019).

60 Guan, J. *et al.* Genome structure variation analyses of peach reveal population dynamics and a 1.67 Mb causal inversion for fruit shape. *Genome Biol.* **22** (2021).

61 Coen, E. S. & Meyerowitz, E. M. The War of the Whorls - Genetic Interactions Controlling Flower Development. *Nature* **353**, 31-37 (1991).

62 Gramzow, L. & Theissen, G. A hitchhiker's guide to the MADS world of plants. *Genome Biol.* **11** (2010).

63 Gramzow, L. & Theissen, G. Phylogenomics reveals surprising sets of essential and dispensable clades of MIKCc-group MADS-box genes in flowering plants. *J. Exp. Zool. Part B* **324**, 353-362 (2015).

64 Theissen, G. & Theissen, G. Stranger than Fiction: Loss of MADS-Box Genes During Evolutionary Miniaturization of the Duckweed Body Plan Loss of MADS-Box Genes in Duckweeds. *Compend Pl Genome*, 91-101 (2020).

65 de Oliveira Silva, F. M. *et al.* The genetic architecture of photosynthesis and plant growth-related traits in tomato. *Plant Cell Environ* **41**, 327-341 (2018).

66 Ellis, C. M. *et al.* AUXIN RESPONSE FACTOR1 and AUXIN RESPONSE FACTOR2 regulate senescence and floral organ abscission in *Arabidopsis thaliana*. *Development* **132**, 4563-4574 (2005).

67 Ravelo-Ortega, G. *et al.* Early sensing of phosphate deprivation triggers the formation of extra root cap cell layers via SOMBRERO through a process antagonized by auxin signaling. *Plant Mol. Biol.* **108**, 77-91 (2022).

68 Schmutz, J. *et al.* A reference genome for common bean and genome-wide analysis of dual domestications. *Nat. Genet.* **46**, 707-713 (2014).

69 Evans, L. M. *et al.* Population genomics of *Populus trichocarpa* identifies signatures of selection and adaptive trait associations. *Nat. Genet.* **46**, 1089-1096 (2014).

70 Guo, S. *et al.* The draft genome of watermelon (*Citrullus lanatus*) and resequencing of 20 diverse accessions. *Nat. Genet.* **45**, 51-58 (2013).

71 Lin, T. *et al.* Genomic analyses provide insights into the history of tomato breeding. *Nat. Genet.* **46**, 1220-1226 (2014).

72 Malinsky, M. *et al.* Whole-genome sequences of *Malawi cichlids* reveal multiple radiations interconnected by gene flow. *Nat. Ecol. Evol.* **2**, 1940-1955 (2018).

73 International Peach Genome, I. *et al.* The high-quality draft genome of peach (*Prunus persica*) identifies unique patterns of genetic diversity, domestication and genome evolution. *Nat. Genet.* **45**, 487-494 (2013).

74 Pont, C. *et al.* Tracing the ancestry of modern bread wheats. *Nat. Genet.* **51**, 905-+ (2019).

75 Pool, J. E. *et al.* Population Genomics of Sub-Saharan *Drosophila melanogaster*: African Diversity and Non-African Admixture. *PLoS Genet.* **8** (2012).

76 Wang, W. *et al.* Genomic variation in 3,010 diverse accessions of Asian cultivated rice. *Nature* **557**, 43-49 (2018).

77 Li, X. *et al.* Whole-genome resequencing of wild and domestic sheep identifies genes associated with morphological and agronomic traits. *Nat Commun* **11**, 2815 (2020).

78 Dutoit, L., Burri, R., Nater, A., Mugal, C. F. & Ellegren, H. Genomic distribution and estimation of nucleotide diversity in natural populations: perspectives from the collared flycatcher (*Ficedula albicollis*) genome. *Mol Ecol Resour* **17**, 586-597 (2017).

79 Genomes Consortium. Electronic address, m. n. g. o. a. a. & Genomes, C. 1,135 Genomes Reveal the Global Pattern of Polymorphism in *Arabidopsis thaliana*. *Cell* **166**, 481-491 (2016).

80 Jorde, L. B. & Wooding, S. P. Genetic variation, classification and 'race'. *Nat. Genet.* **36**, S28-33 (2004).

81 Lijavetzky, D., Cabezas, J. A., Ibanez, A., Rodriguez, V. & Martinez-Zapater, J. M. High throughput SNP discovery and genotyping in grapevine (*Vitis vinifera* L.) by combining a re-sequencing approach and SNPlex technology. *BMC Genomics* **8**, 424 (2007).
