# Supplementary material for: Population genomics and epigenomics of Spirodela polyrhiza provide insights into the evolution of facultative asexuality
Source: Commun Biol. 2024 May 16;7:581. doi: 10.1038/s42003-024-06266-7 (PMC11099151; doi:10.1038/s42003-024-06266-7)
Supplement: Supplementary file 3 — Description of Additional Supplementary Files [file 42003_2024_6266_MOESM3_ESM.pdf]

Description of Additional Supplementary Files

File name: Supplementary Data 1
Description: The file describes detailed information on all 228 *S. polyrhiza* genotypes, including their sequencing statistics, sampling regions, and Accession numbers in NCBI.

File name: Supplementary Data 2
Description: The table contains the annotation of all SNPs. Each row records one annotated gene. The first column is the gene ID, and the following columns are the number of SNPs annotated as different categories.

File name: Supplementary Data 3
Description: The table contains all the annotation information of SVs.

File name: Supplementary Data 4
Description: The table contains gene families as well as members from each gene family identified in the *S. polyrhiza* genome.

File name: Supplementary Data 5
Description: The table contains the results of enrichment analysis of small INDELs and SVs among all the gene families identified.

File name: Supplementary Data 6
Description: The table contains the grouping of clonal families of all 228 *S. polyrhiza* genotypes.

File name: Supplementary Data 7
Description: The file contains the alignment (against to the homologs from *Arabidopsis thaliana*) and expression of 34 candidate genes that were discussed in the manuscript.

File name: Supplementary Data 8
Description: The table contains the list of genes detected under positive selection at the species-wide level according to RAiSD, SweeD and LASSI.

File name: Supplementary Data 9
Description: This table contains the list of genes (rows) belonging to the top 1% CLR values of 3P-CLR and the population branches where they are under selection (columns). Fields with “1” indicate that a signature of selection was found, and fields with “0” indicate no signature of selection was found.

File name: Supplementary Data 10
Description: The file contains detailed information for the NCBI CDD check of annotated MADS-box TFs. The first sheet records all 43 MADS-box TFs that passed the CDD check, while the second sheet records other candidates that failed the CDD check.

File name: **Supplementary Data 11**

Description: The file contains the list of differentially methylated regions (DMRs) genes, which are also found to be under selection in the corresponding populations. Additional columns recorded the Arabidopsis orthologs; accordingly, presumed functions were also provided.

File name: **Supplementary Data 12**

Description: The file contains the list of differentially expressed genes (DEGs) that are also found to be under selection in the corresponding populations. Again, those genes' Arabidopsis orthologs and potential functions were provided.
