# Supplementary material for: Population genomics and epigenomics of Spirodela polyrhiza provide insights into the evolution of facultative asexuality
Source: Commun Biol. 2024 May 16;7:581. doi: 10.1038/s42003-024-06266-7 (PMC11099151; doi:10.1038/s42003-024-06266-7)

**Validation and expression of candidate genes.**

| **Candidate gene** | **Ortholog/homolog in Arabidopsis** | **Common name** | **Page** |
| --- | --- | --- | --- |
| SpGA2022_052159 | AT3G59420.1 | *ACR4* | 2 |
| SpGA2022_013078 | AT2G45650.1 | *AGL6* | 3 |
| SpGA2022_005278 | AT5G60440.1 | *AGL62* | 4 |
| SpGA2022_015550 | AT5G55730.1 | *AGP* | 5 |
| SpGA2022_052274 | AT5G20240.1 | *APETALA3* | 6 |
| SpGA2022_053541 | AT3G48190.2 | *ATM* | 7 |
| SpGA2022_006111 | AT3G63530.1 | *BB* | 10 |
| SpGA2022_051517 | AT1G69770.1 | *CMT3* | 11 |
| SpGA2022_010831 | AT5G18420.2 | *CCR4-NOT* | 12 |
| SpGA2022_050728 | AT1G18040.1 | *CDK* | 13 |
| SpGA2022_053214 | AT5G49890.1 | *CLC* | 14 |
| SpGA2022_015102 | AT2G01730.1 | *CPSF* | 15 |
| SpGA2022_055195 | AT3G61880.2 | *CYP78A9* | 16 |
| SpGA2022_052378 | AT1G58220.1 | *DRMY1* | 17 |
| SpGA2022_052156 | AT1G05960.2 | *EFOP3* | 18 |
| SpGA2022_015101 | AT3G60260.1 | *ELMOD* | 20 |
| SpGA2022_053158 | AT5G26030.1 | *FC1* | 21 |
| SpGA2022_013448 | AT3G04610.1 | *FLK* | 22 |
| SpGA2022_055227 | AT2G33680.5 | *GEND1* | 23 |
| SpGA2022_010691 | AT2G27470.1 | *NF-Y_AT2G27470* | 24 |
| SpGA2022_005107 | AT5G52820.1 | *NOTCHLESS* | 25 |
| SpGA2022_054699 | AT5G47650.2 | *NUDT2* | 26 |
| SpGA2022_002887 | AT1G55540.2 | *NUP214* | 27 |
| SpGA2022_054509 | AT2G34710.1 | *PHABULOSA/PHAVOLUTA* | 29 |
| SpGA2022_055362 | AT1G07990.1 | *PPP* | 30 |
| SpGA2022_014906 | AT5G52580.2 | *RabGAP* | 31 |
| SpGA2022_007853 | AT4G02790.1 | *RbgA* | 32 |
| SpGA2022_052273 | AT1G21650.3 | *SECA2* | 33 |
| SpGA2022_007306 | AT2G45660.1 | *SOC1* | 35 |
| SpGA2022_006905 | AT2G22540.1 | *SVP-*group | 36 |
| SpGA2022_051406 | AT3G09080.3 | *Transducin/WD40* | 37 |
| SpGA2022_055984 | AT5G20520.1 | *WAVY* | 39 |
| SpGA2022_014600 | AT5G57450.1 | *Xrcc3* | 40 |
| SpGA2022_051355 | AT2G42010.2 | *phospholipase_D* | 41 |

# SpGA2022_052159 (*ACR4*)

**Putative function:** Similar to CR4: Serine/threonine-protein kinase-like protein CR4 (*Oryza sativa* subsp. japonica)

***Arabidopsis* ortholog/homolog:** AT3G59420.1

**Alignment:**


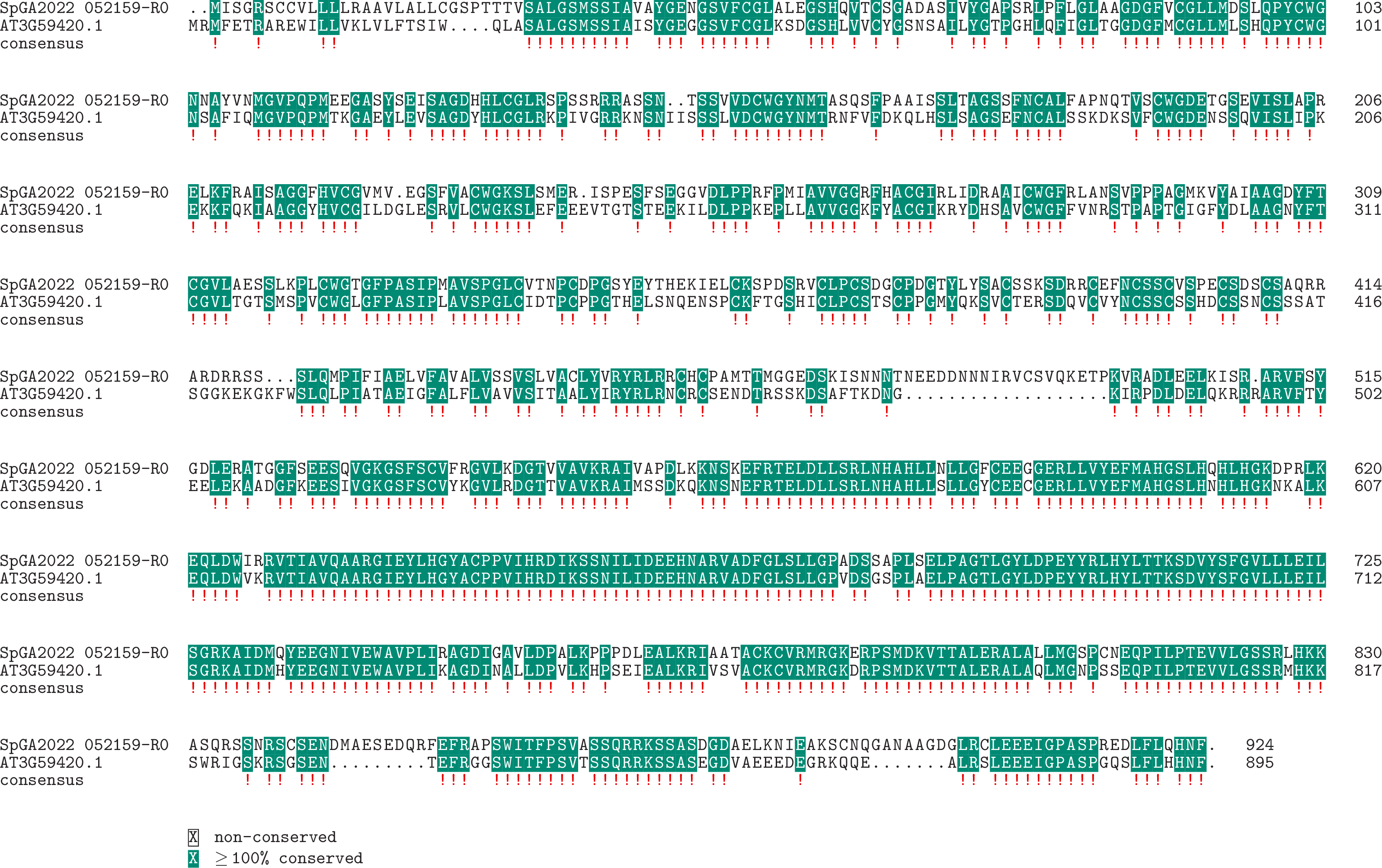


**Expression:**


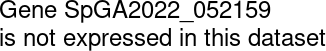


# SpGA2022_013078 (*AGL6*)

**Putative function:** Similar to MADS6: MADS-box transcription factor 6 (*Oryza sativa* subsp. japonica)

***Arabidopsis* ortholog/homolog:** AT2G45650.1

**Alignment:**


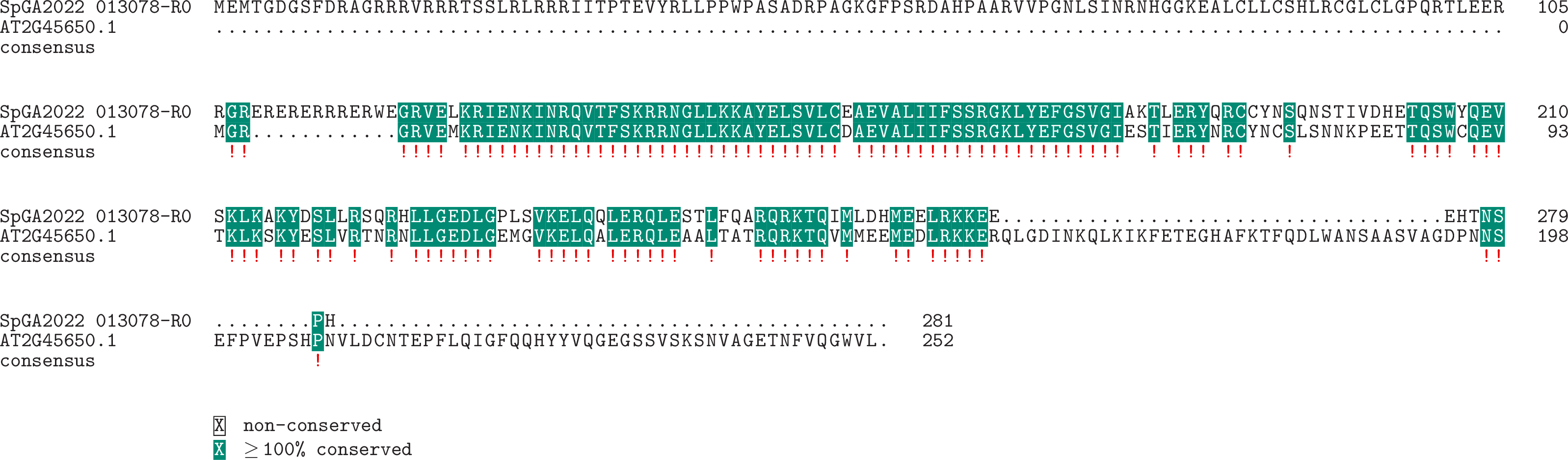


**Expression:**


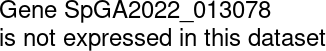


# SpGA2022_005278 (*AGL62*)

**Putative function:** Similar to AGL62: Agamous-like MADS-box protein AGL62 (*Arabidopsis thaliana*)

***Arabidopsis* ortholog/homolog:** AT5G60440.1

**Alignment:**


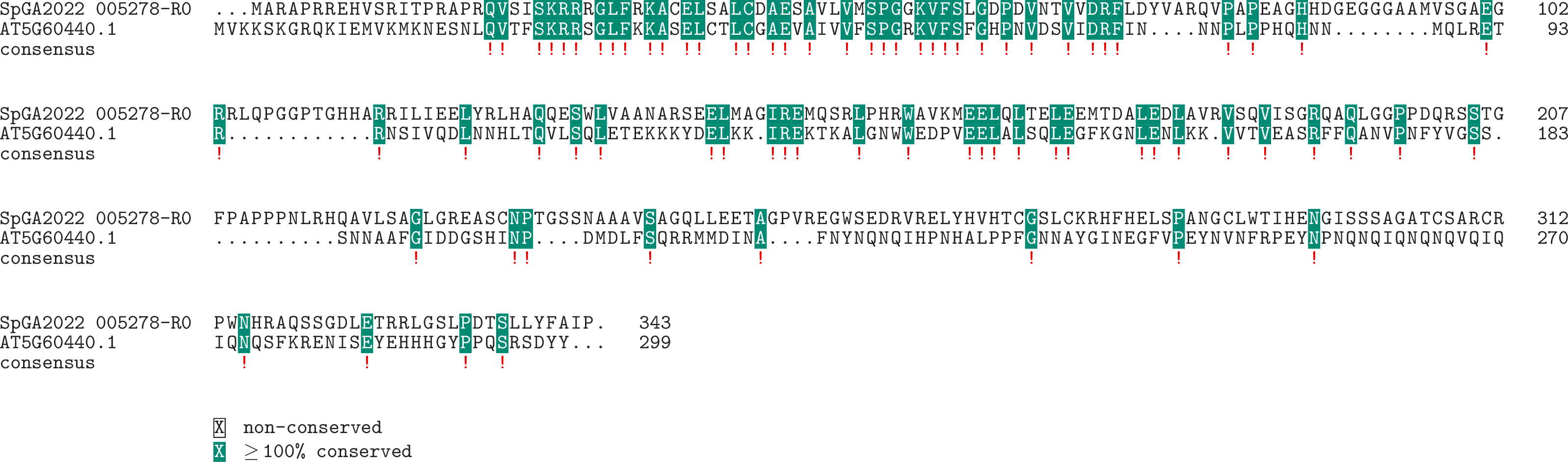


**Expression:**


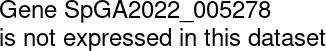


# SpGA2022_015550 (*AGP*)

**Putative function:** Similar to FLA1: Fasciclin-like arabinogalactan protein 1 (*Arabidopsis thaliana*)

***Arabidopsis* ortholog/homolog:** AT5G55730.1

**Alignment:**


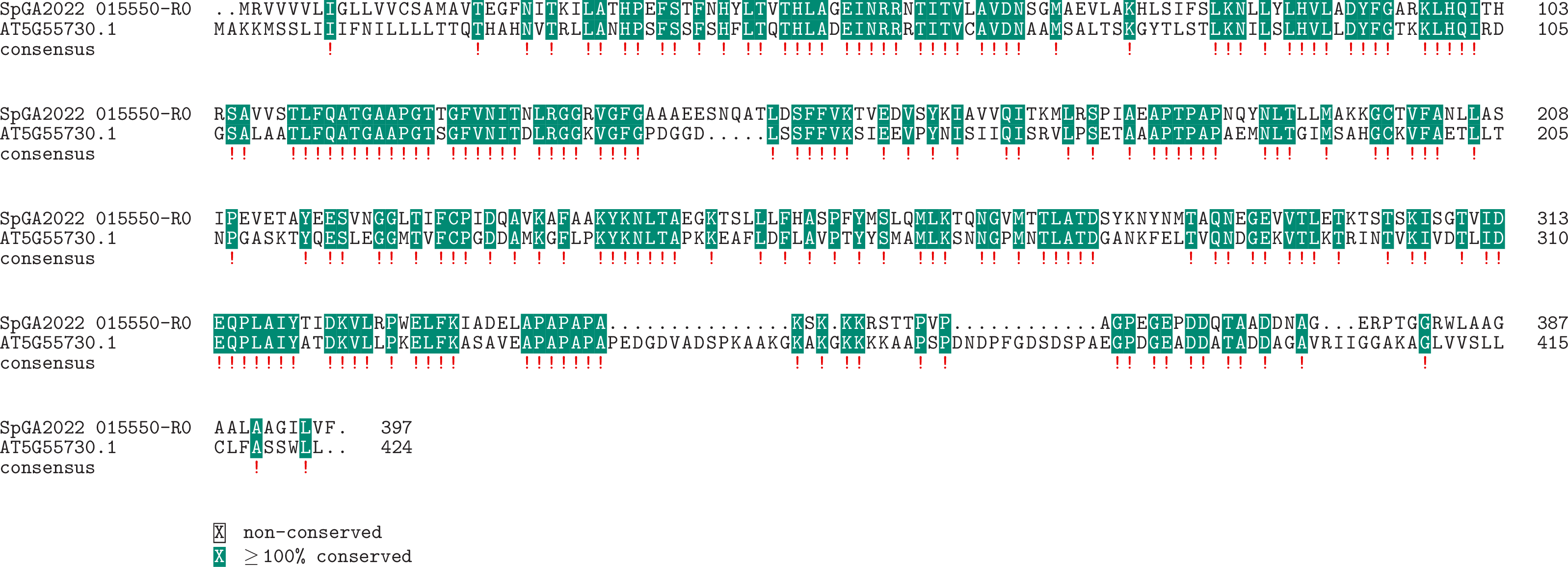


**Expression:**


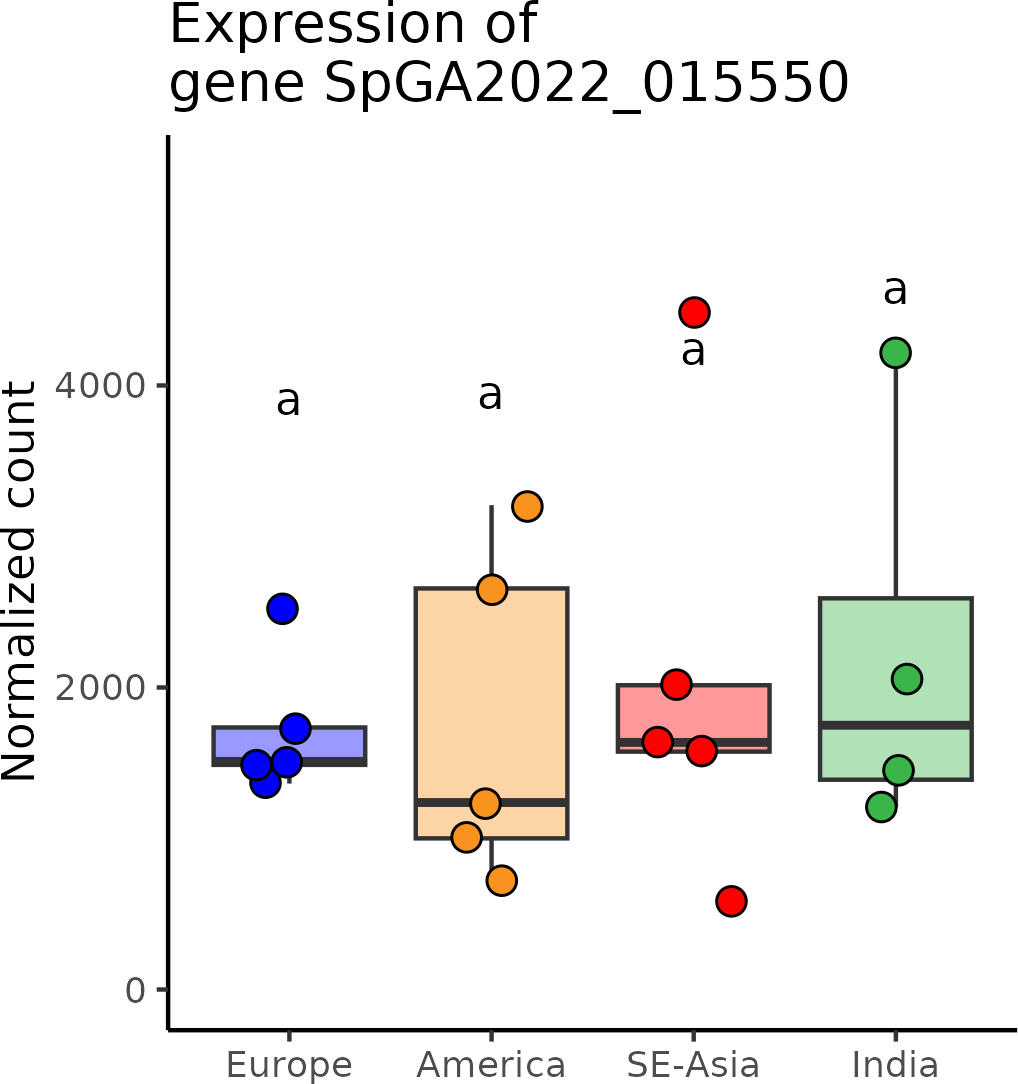


# SpGA2022_052274 (*APETALA3*)

**Putative function:** Similar to MADS9: Agamous-like MADS-box protein MADS9 (*Vitis vinifera*)

***Arabidopsis* ortholog/homolog:** AT5G20240.1

**Alignment:**


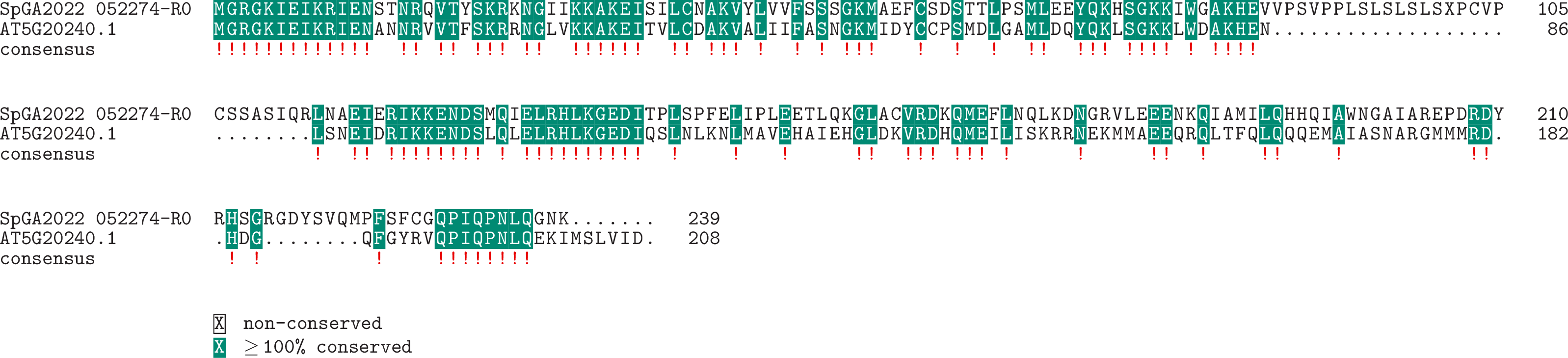


**Expression:**


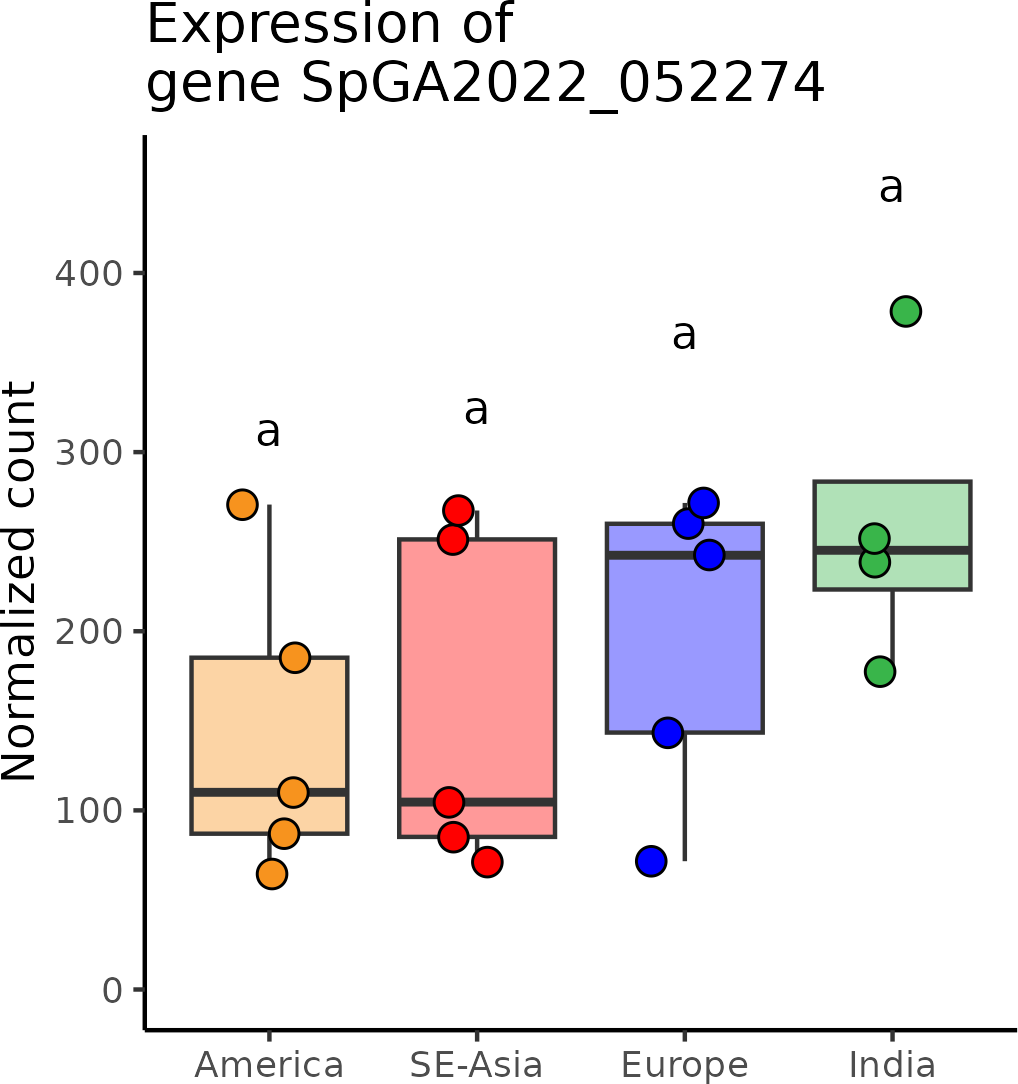


# SpGA2022_053541 (*ATM*)

**Putative function:** Similar to ATM: Serine/threonine-protein kinase ATM (*Arabidopsis thaliana*)

***Arabidopsis* ortholog/homolog:** AT3G48190.2

**Alignment:**


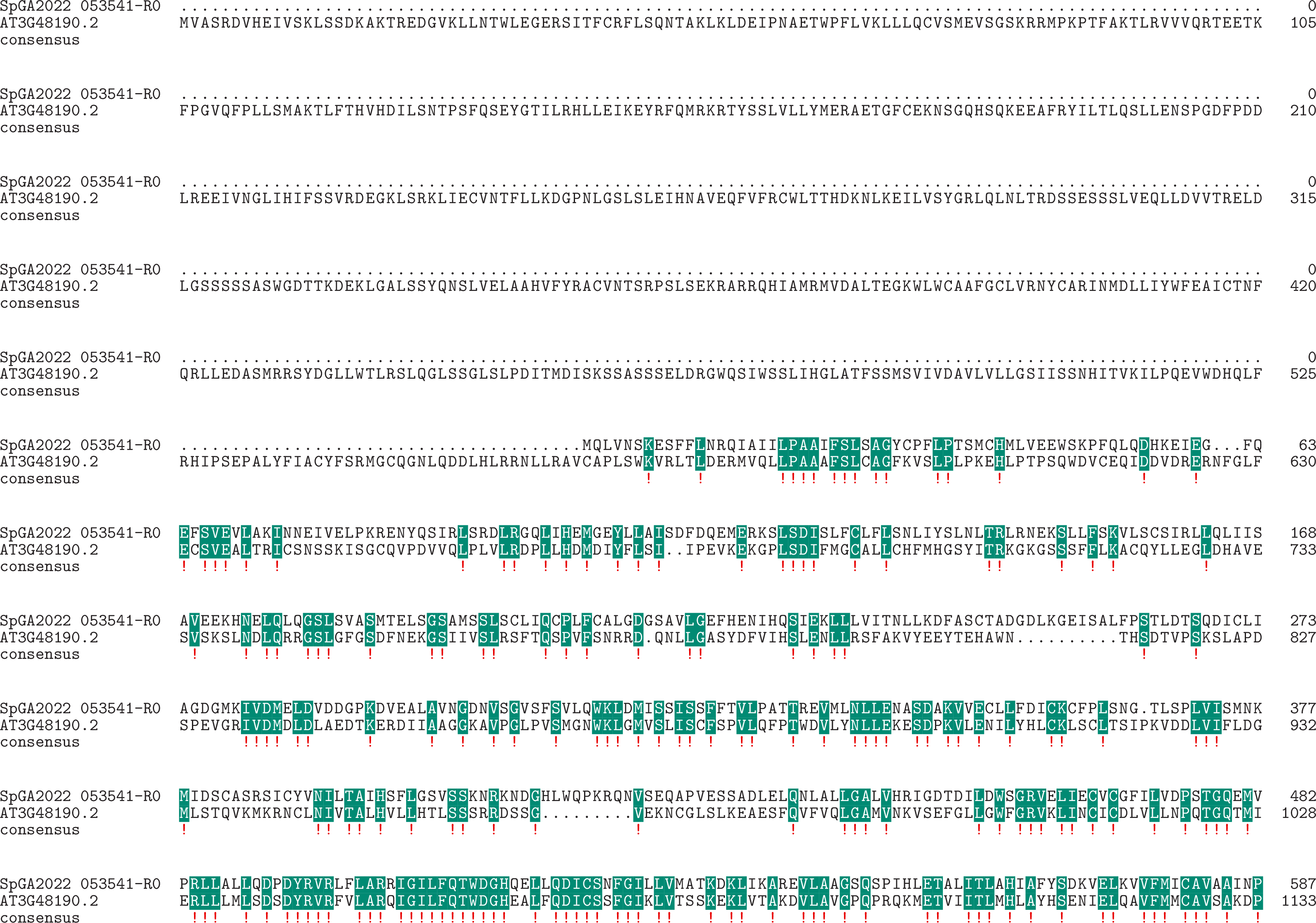


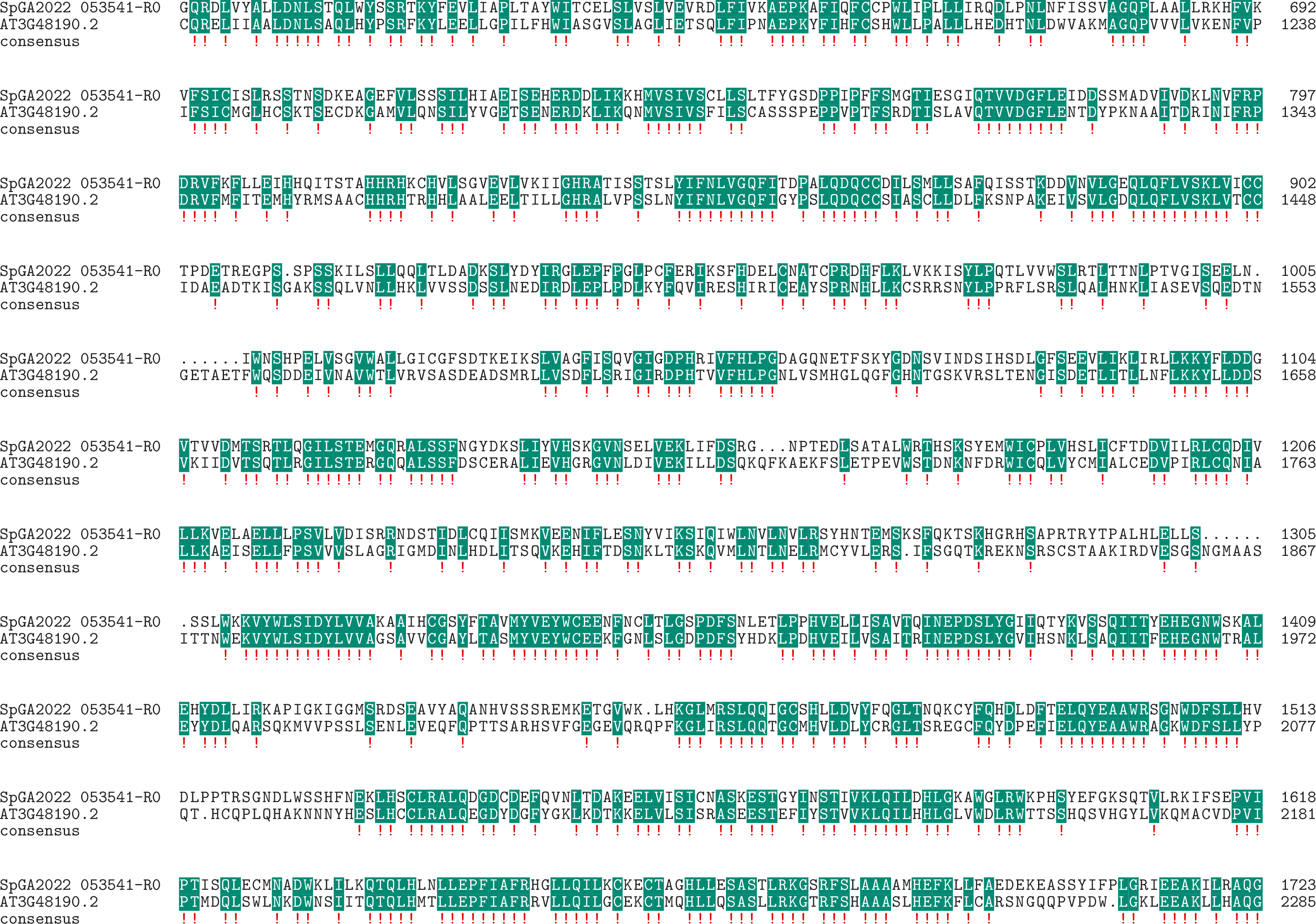


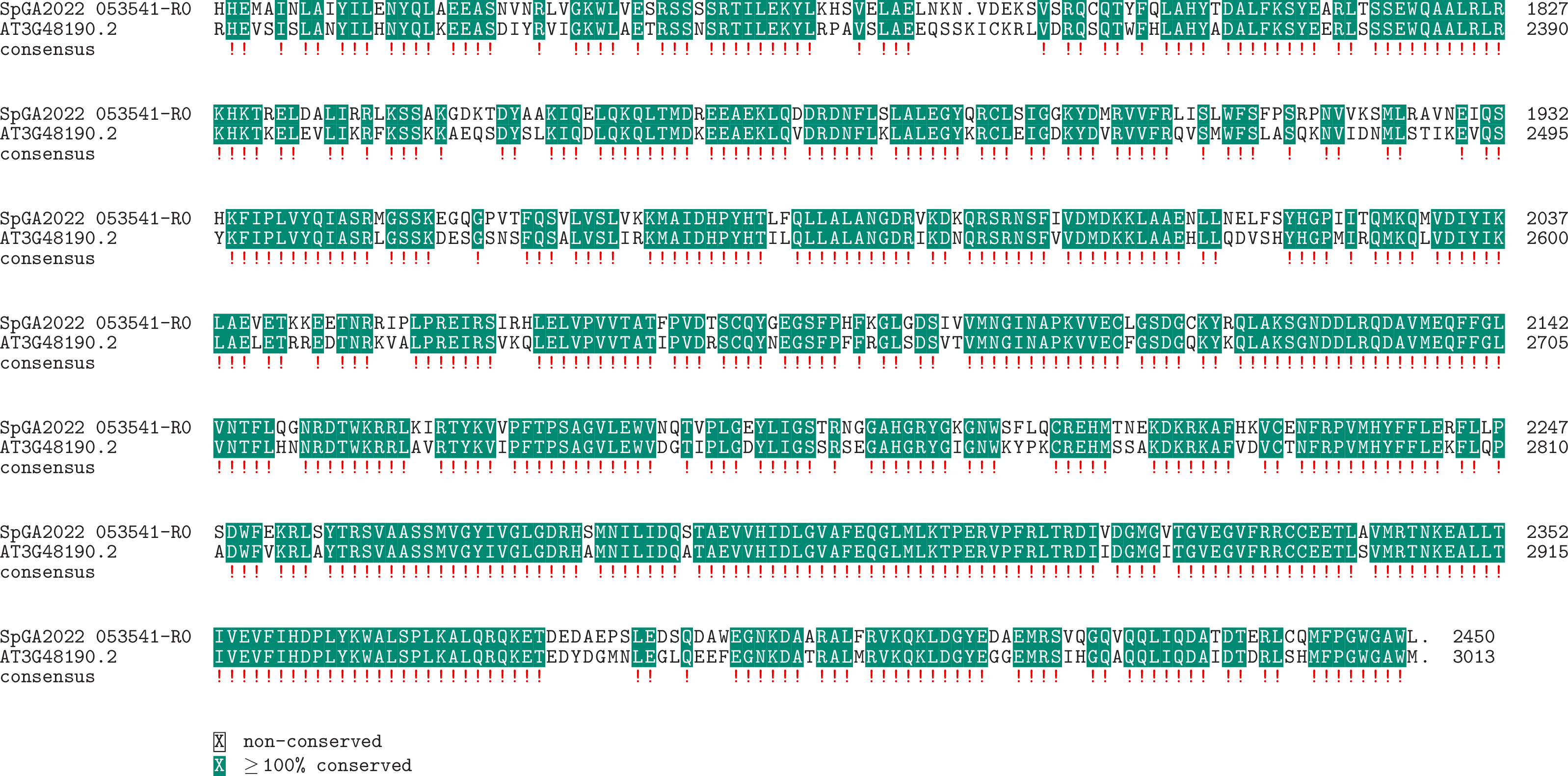


**Expression:**


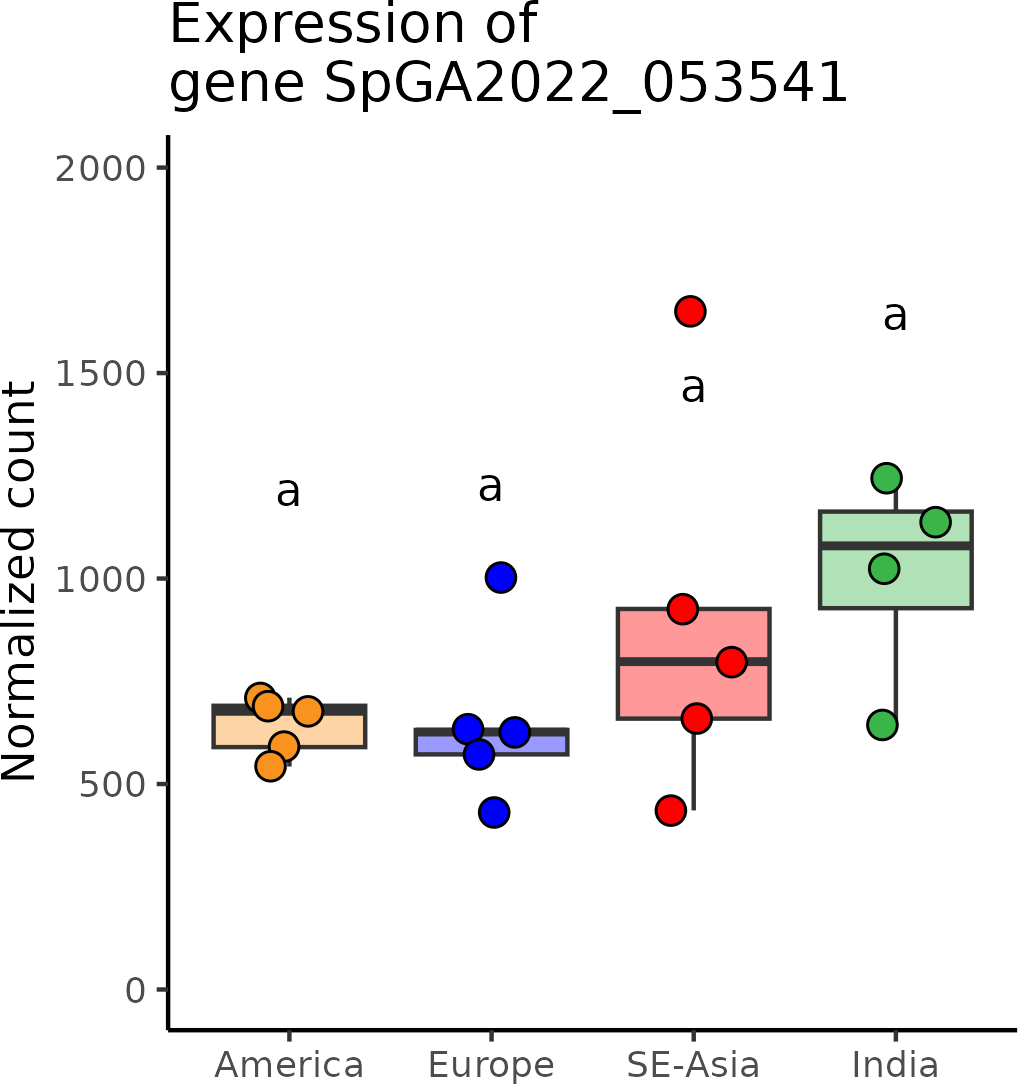


# SpGA2022_006111 (*BB*)

**Putative function:** Similar to BB: E3 ubiquitin-protein ligase BIG BROTHER (*Arabidopsis thaliana*)

***Arabidopsis* ortholog/homolog:** AT3G63530.1

**Alignment:**


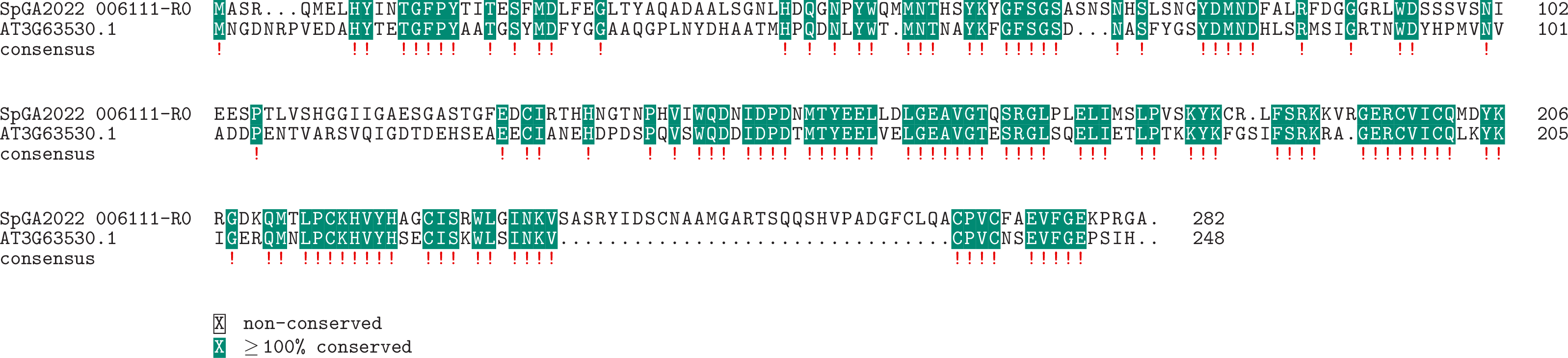


**Expression:**


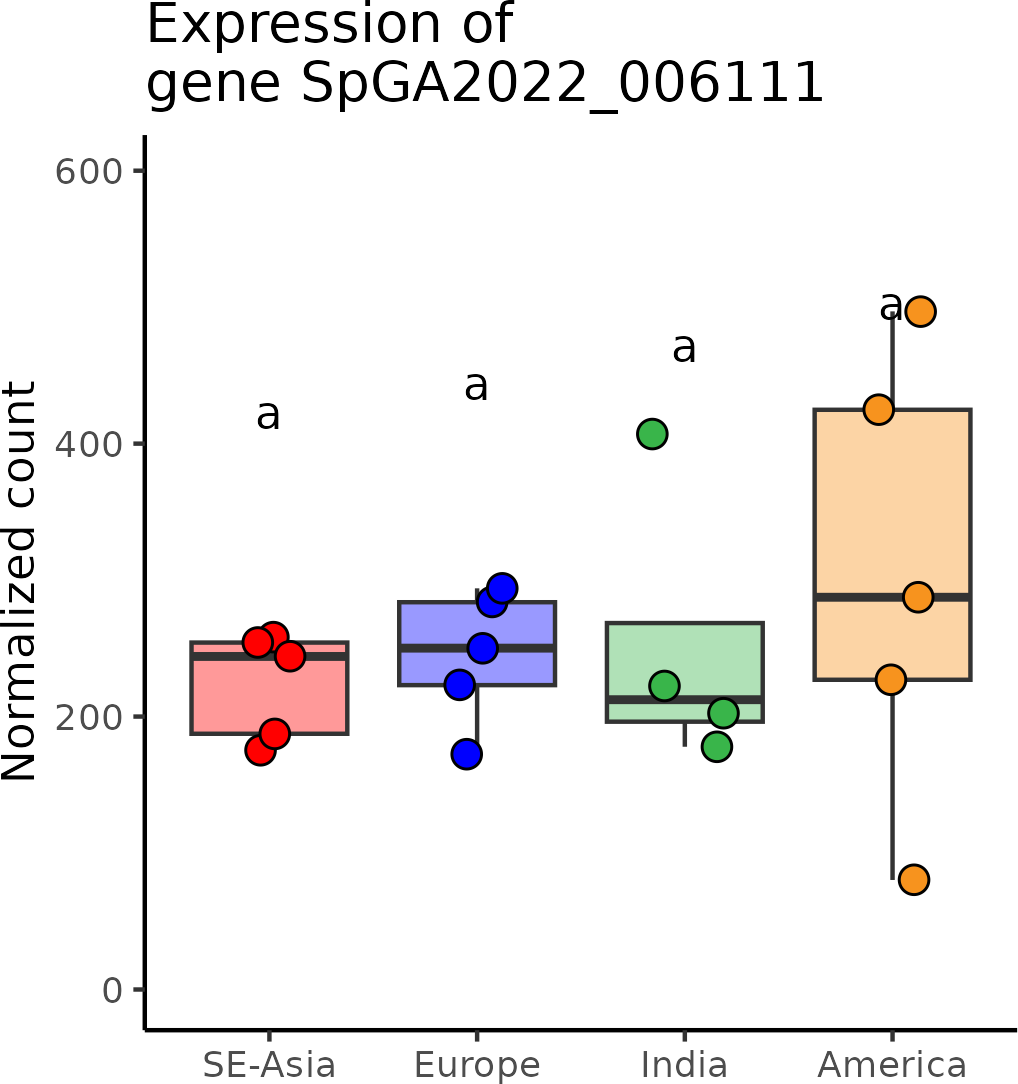


# SpGA2022_051517 (*CMT3*)

**Putative function:** Similar to CMT1: DNA (cytosine-5)-methyltransferase CMT1 (*Oryza sativa* subsp. japonica)

***Arabidopsis* ortholog/homolog:** AT1G69770.1

**Alignment:**


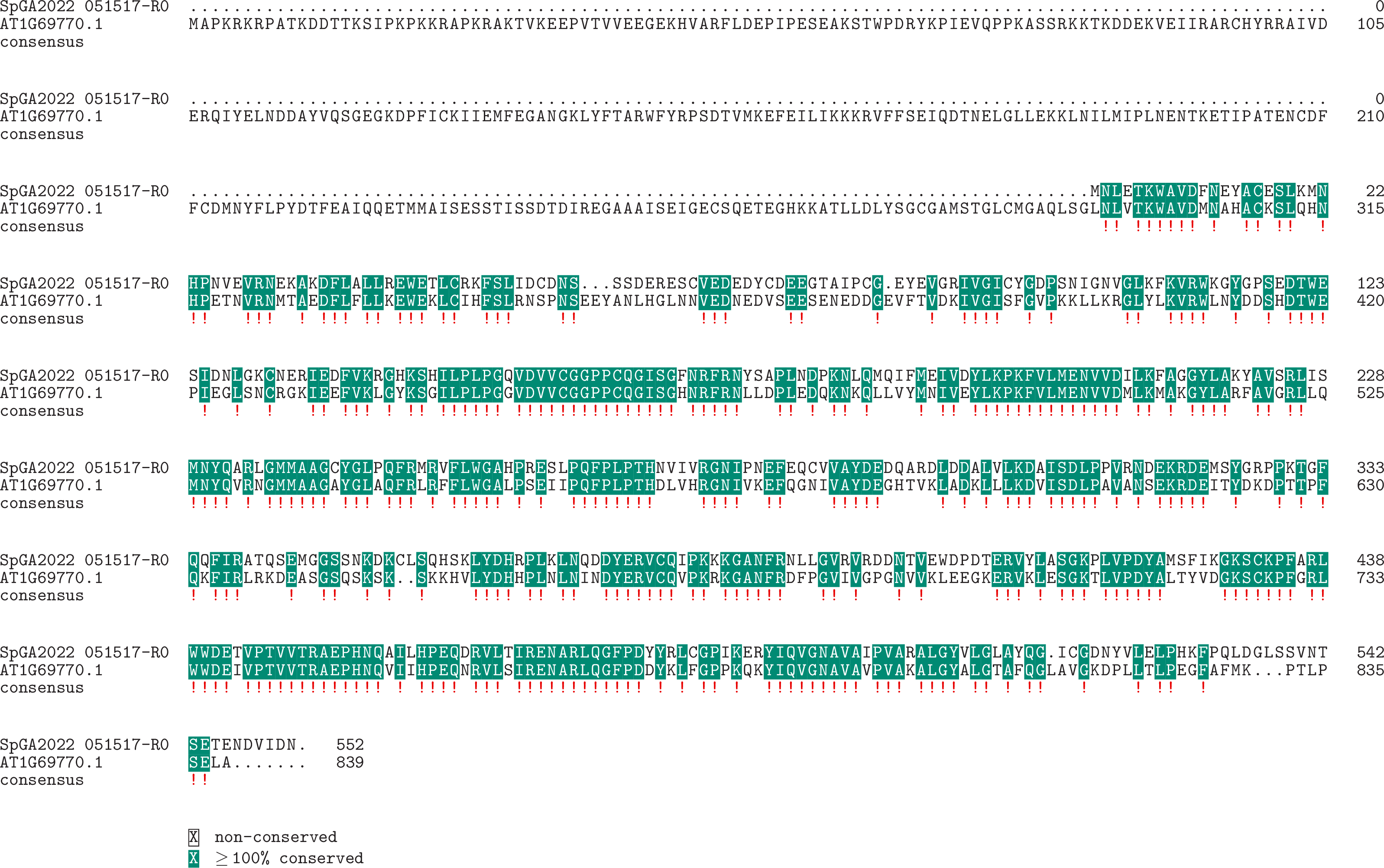


**Expression:**


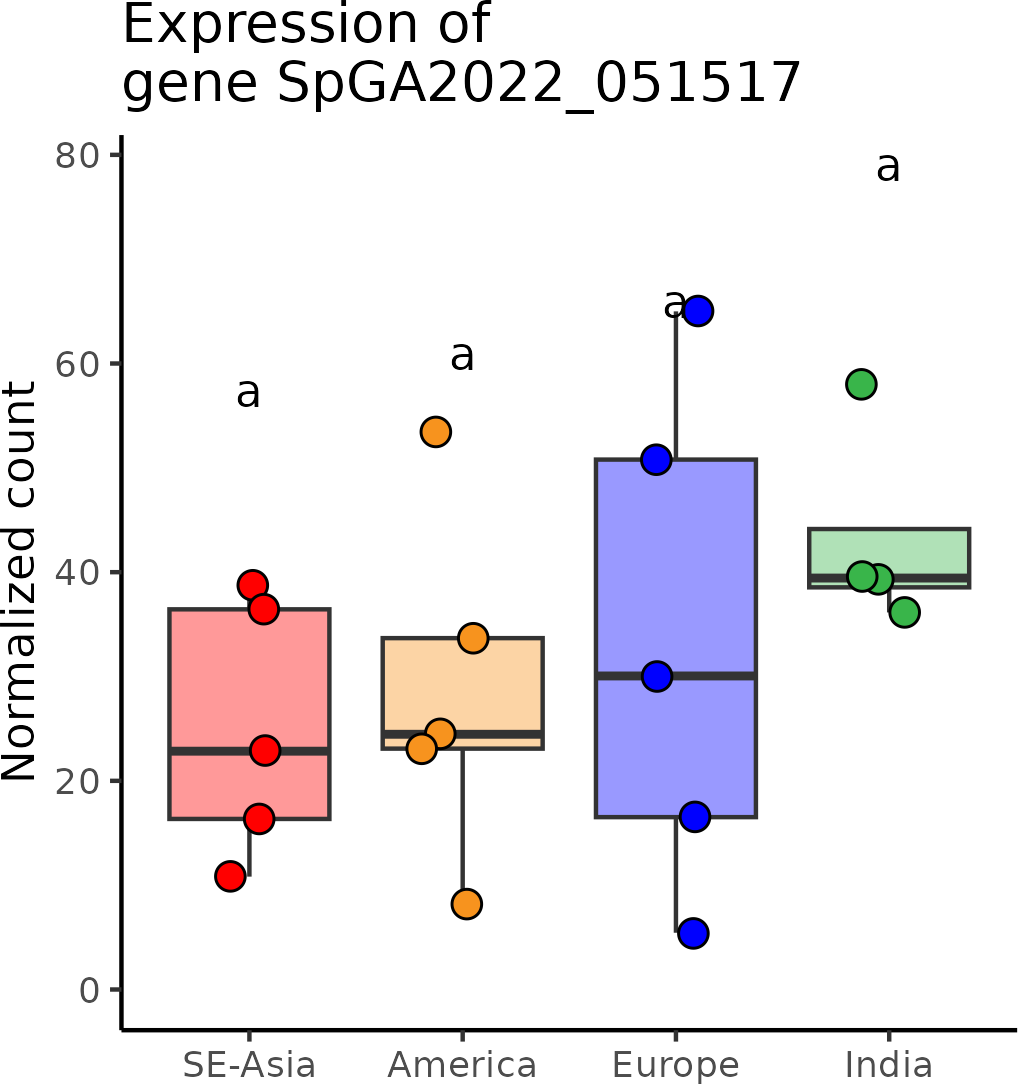


# SpGA2022_010831 (*CCR4-NOT*)

**Putative function:** Similar to Cnot11: CCR4-NOT transcription complex subunit 11 (*Rattus norvegicus*)

***Arabidopsis* ortholog/homolog:** AT5G18420.2

**Alignment:**


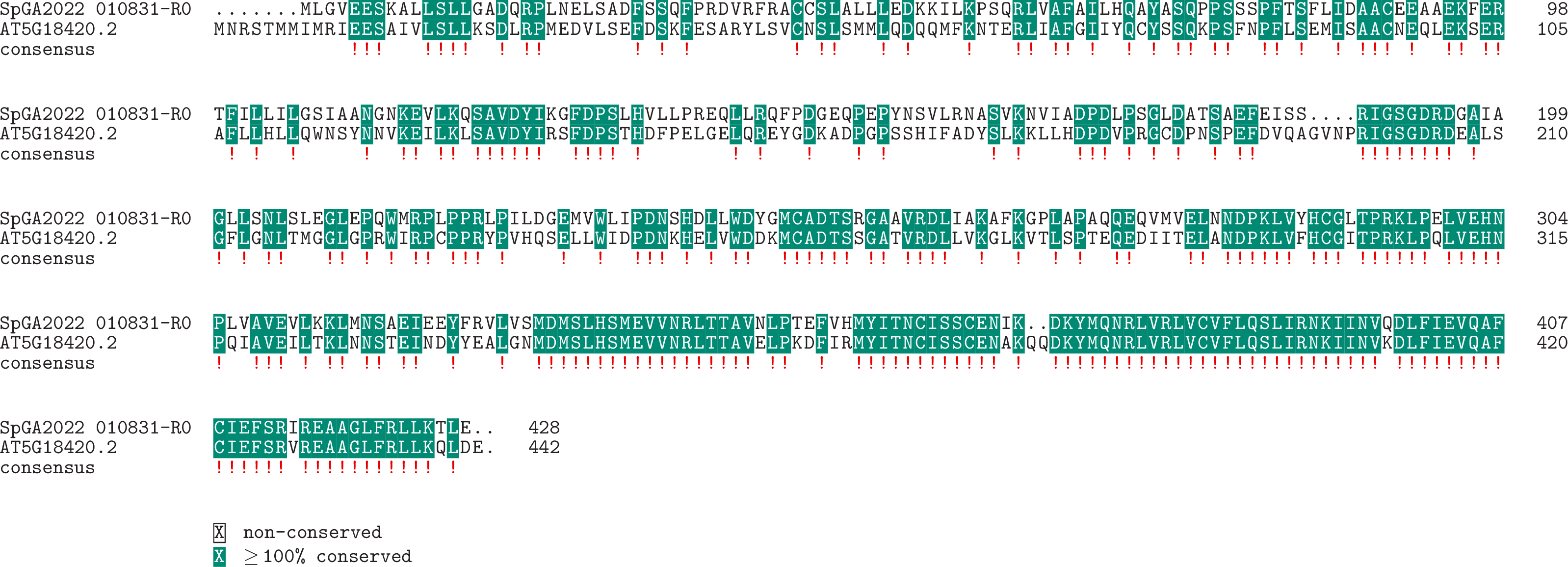


**Expression:**


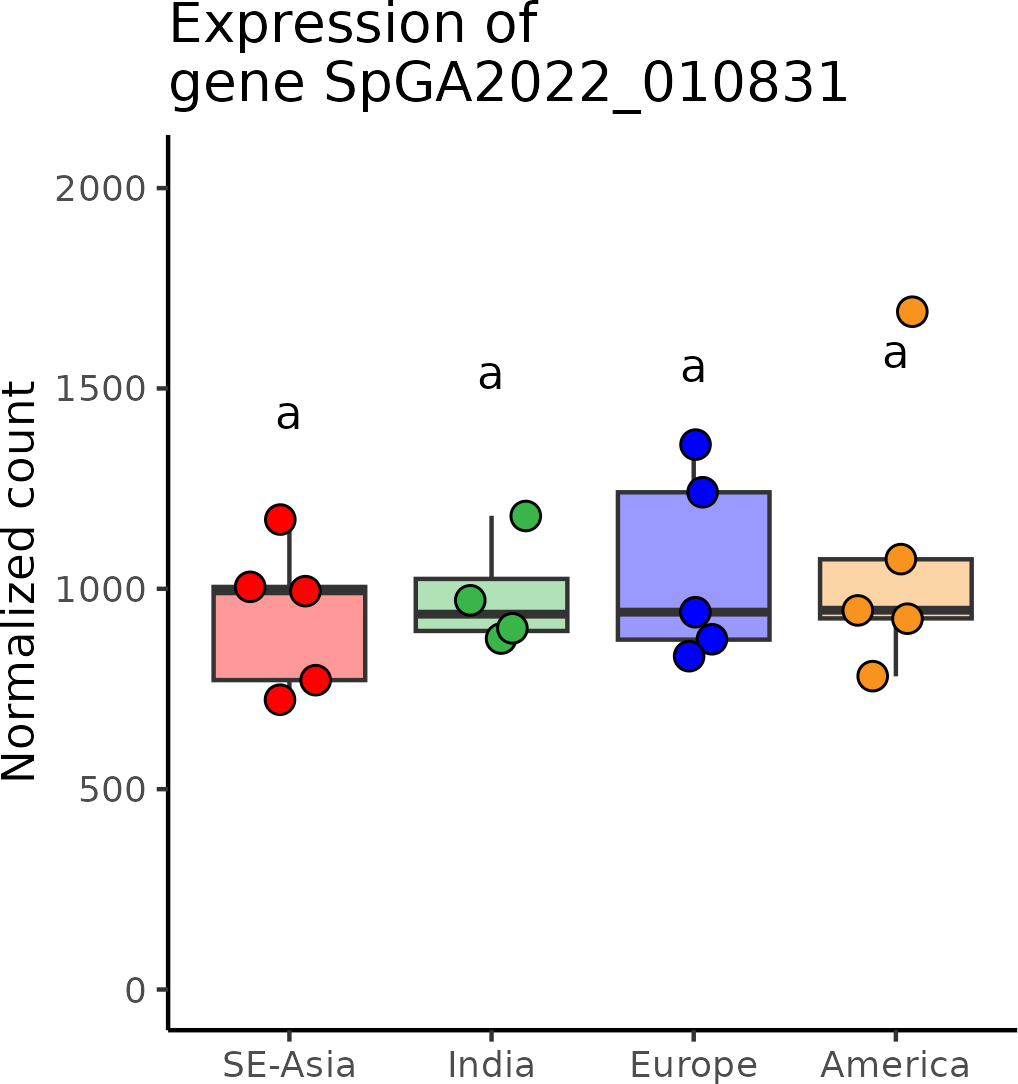


# SpGA2022_050728 (*CDK*)

**Putative function:** Similar to CDKD-1: Cyclin-dependent kinase D-1 (*Oryza sativa* subsp. japonica)

***Arabidopsis* ortholog/homolog:** AT1G18040.1

**Alignment:**


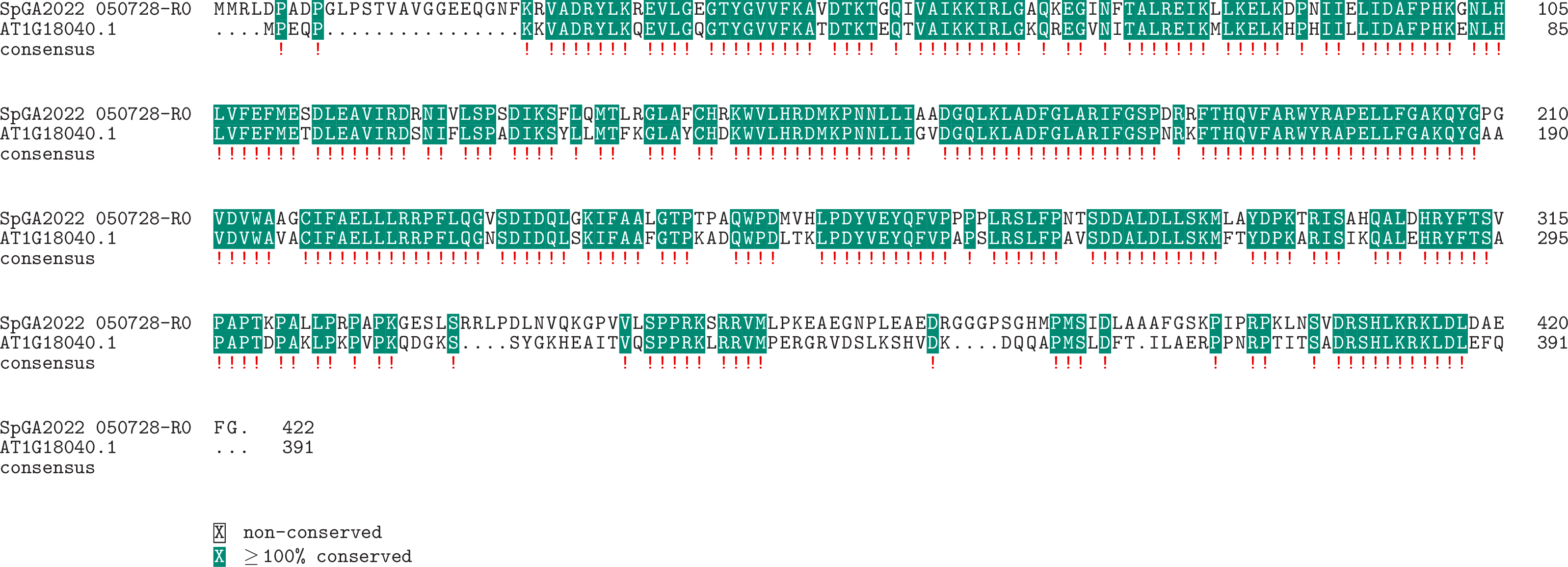


**Expression:**


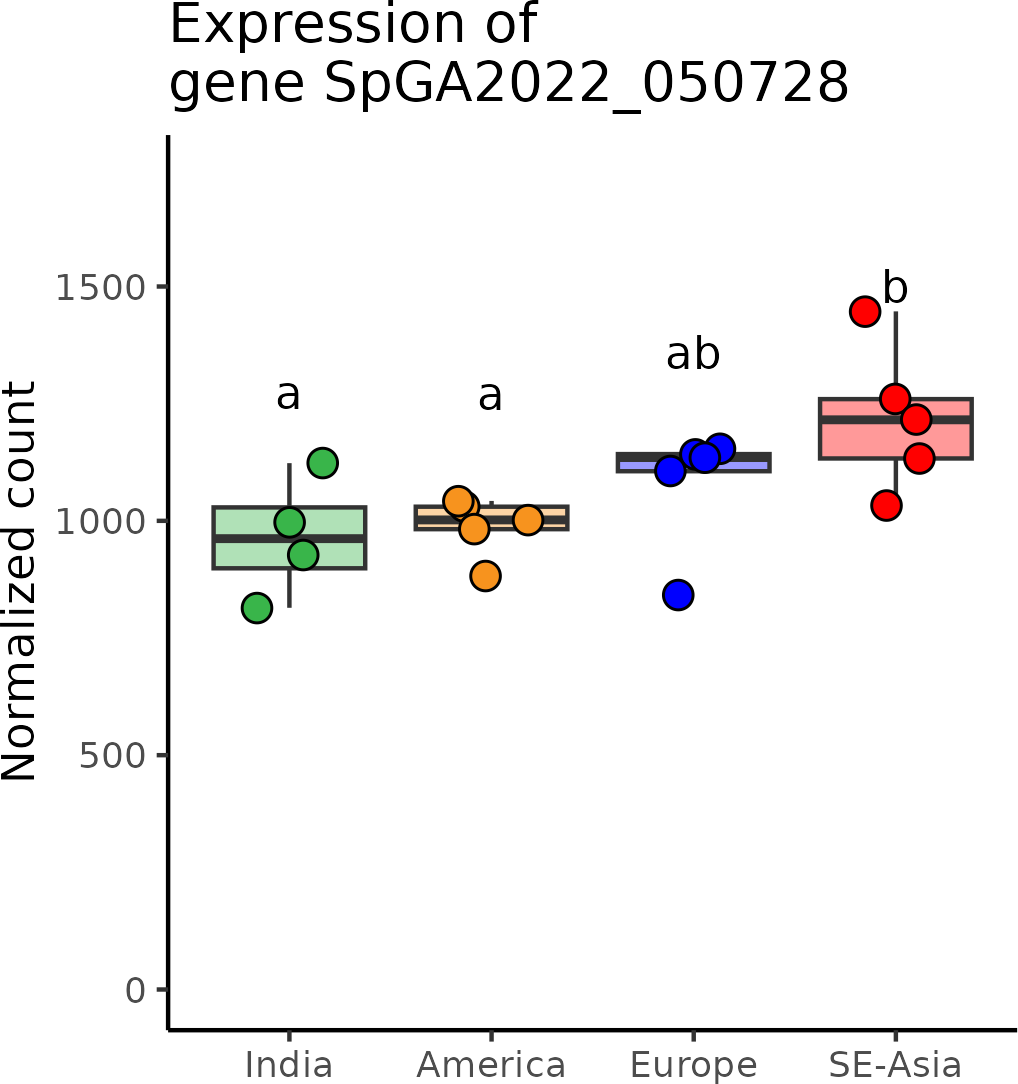


# SpGA2022_053214 (*CLC*)

**Putative function:** Similar to CLC-C: Chloride channel protein CLC-c (*Arabidopsis thaliana*)

***Arabidopsis* ortholog/homolog:** AT5G49890.1

**Alignment:**


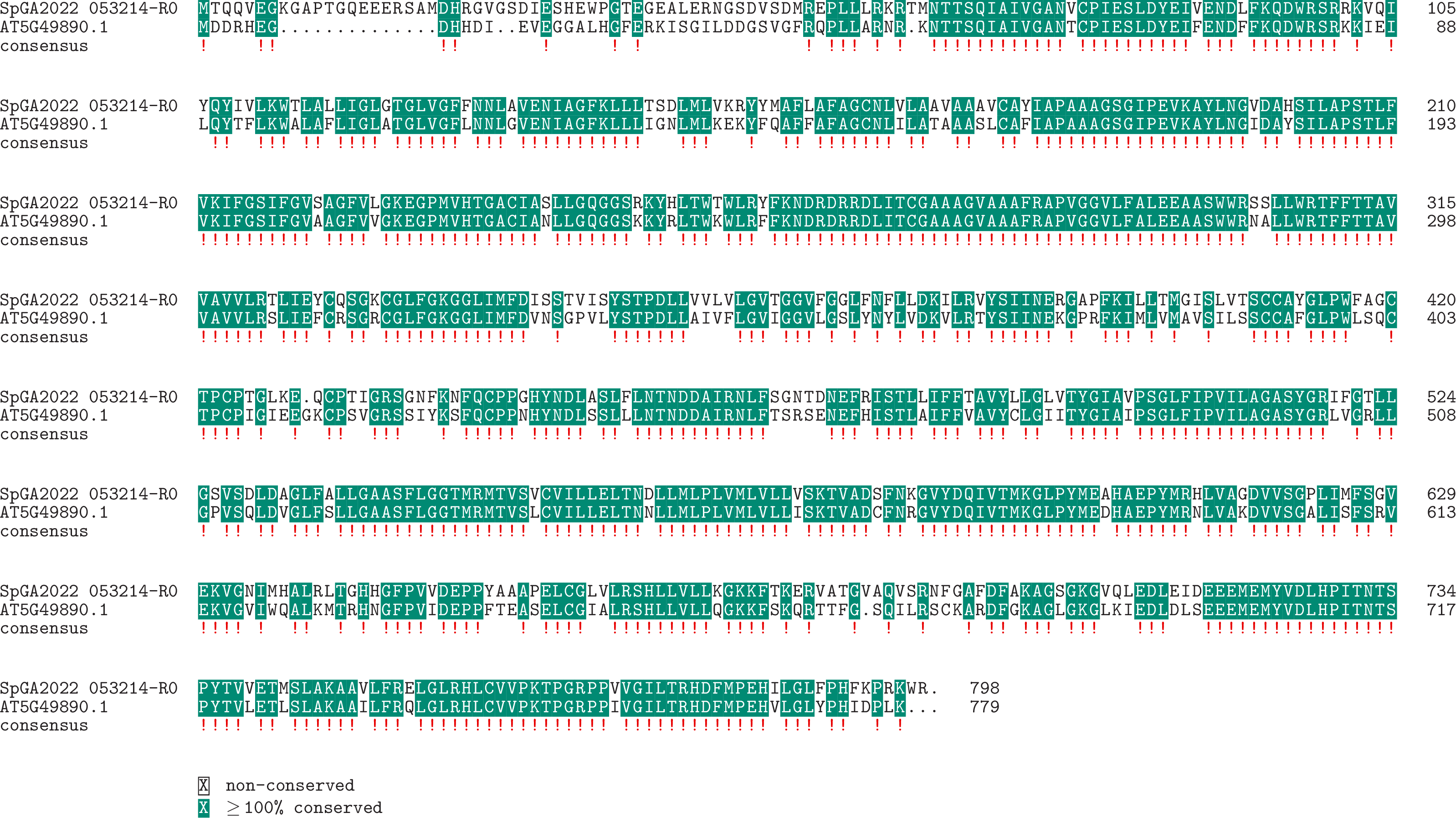


**Expression:**


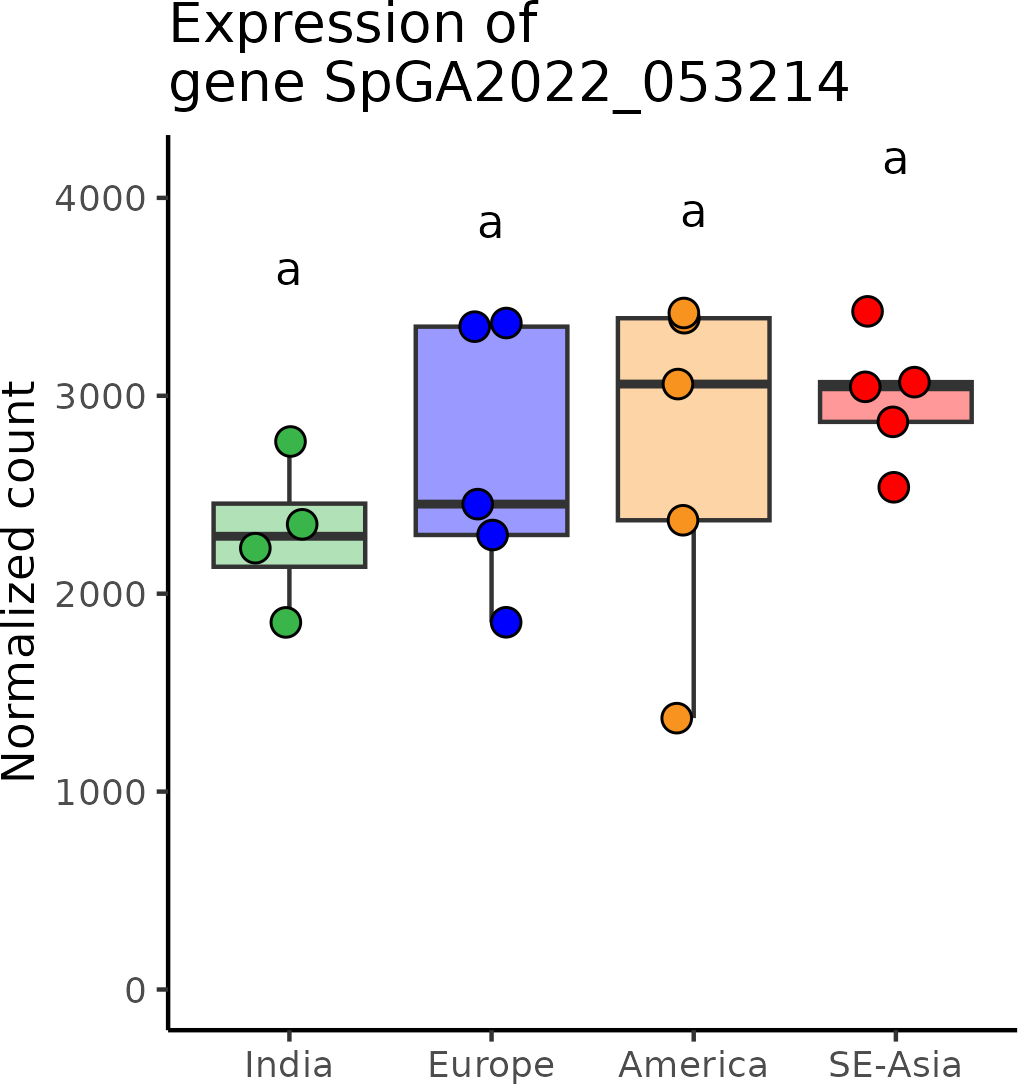


# SpGA2022_015102 (*CPSF*)

**Putative function:** Similar to CPSF73-II: Cleavage and polyadenylation specificity factor subunit 3-II (*Arabidopsis thaliana*)

***Arabidopsis* ortholog/homolog:** AT2G01730.1

**Alignment:**


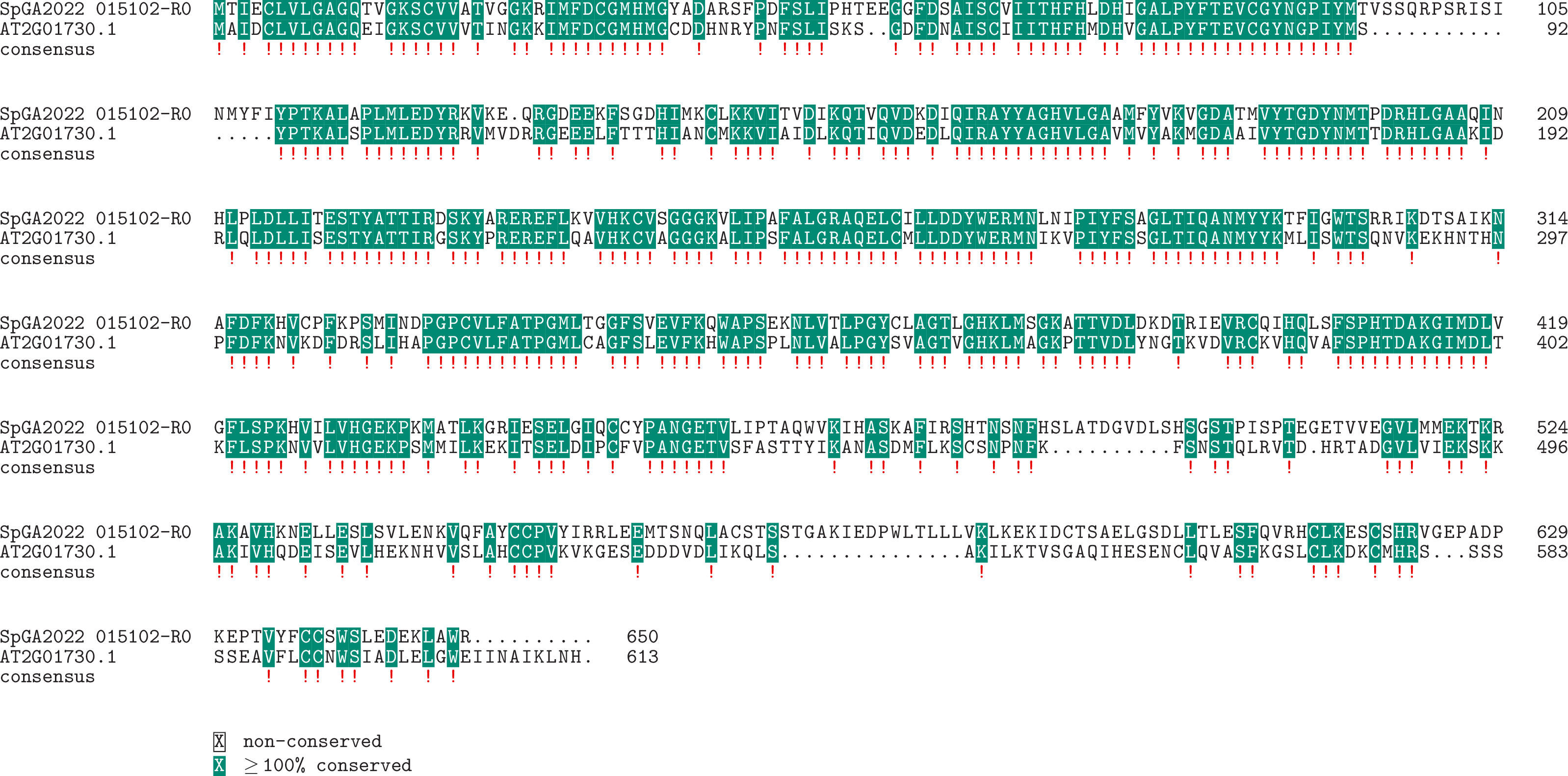


**Expression:**


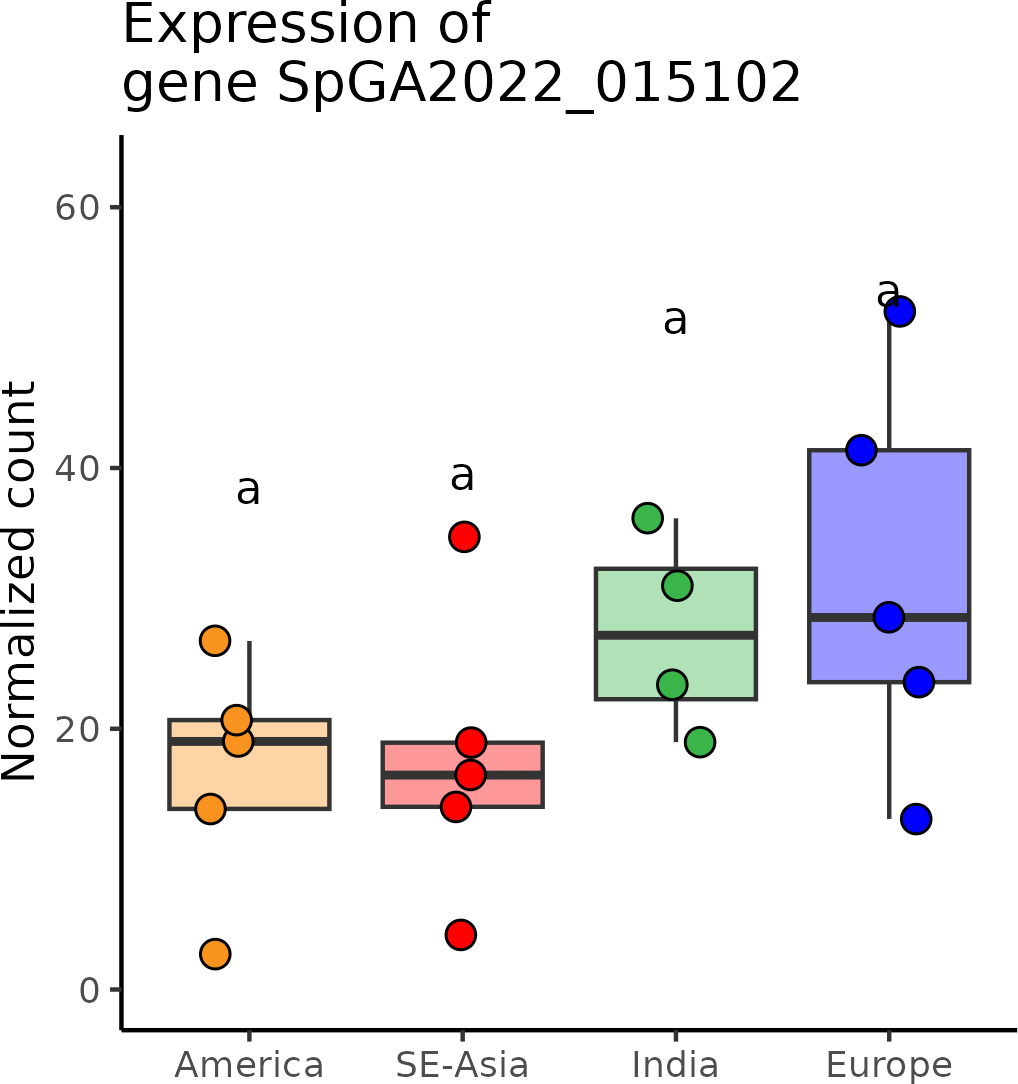


# SpGA2022_055195 (*CYP78A9*)

**Putative function:** Similar to CYP78A6: Cytochrome P450 78A6 (*Arabidopsis thaliana*)

***Arabidopsis* ortholog/homolog:** AT3G61880.2

**Alignment:**


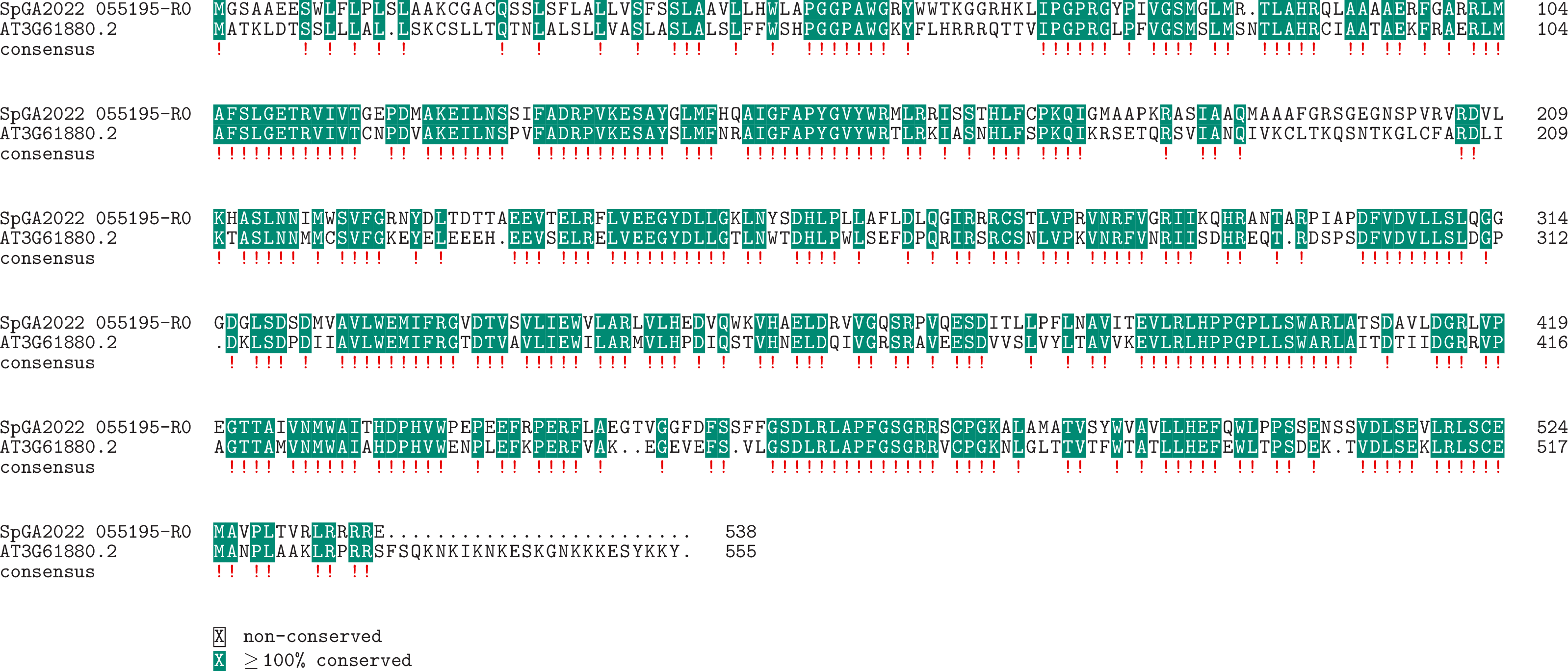


**Expression:**


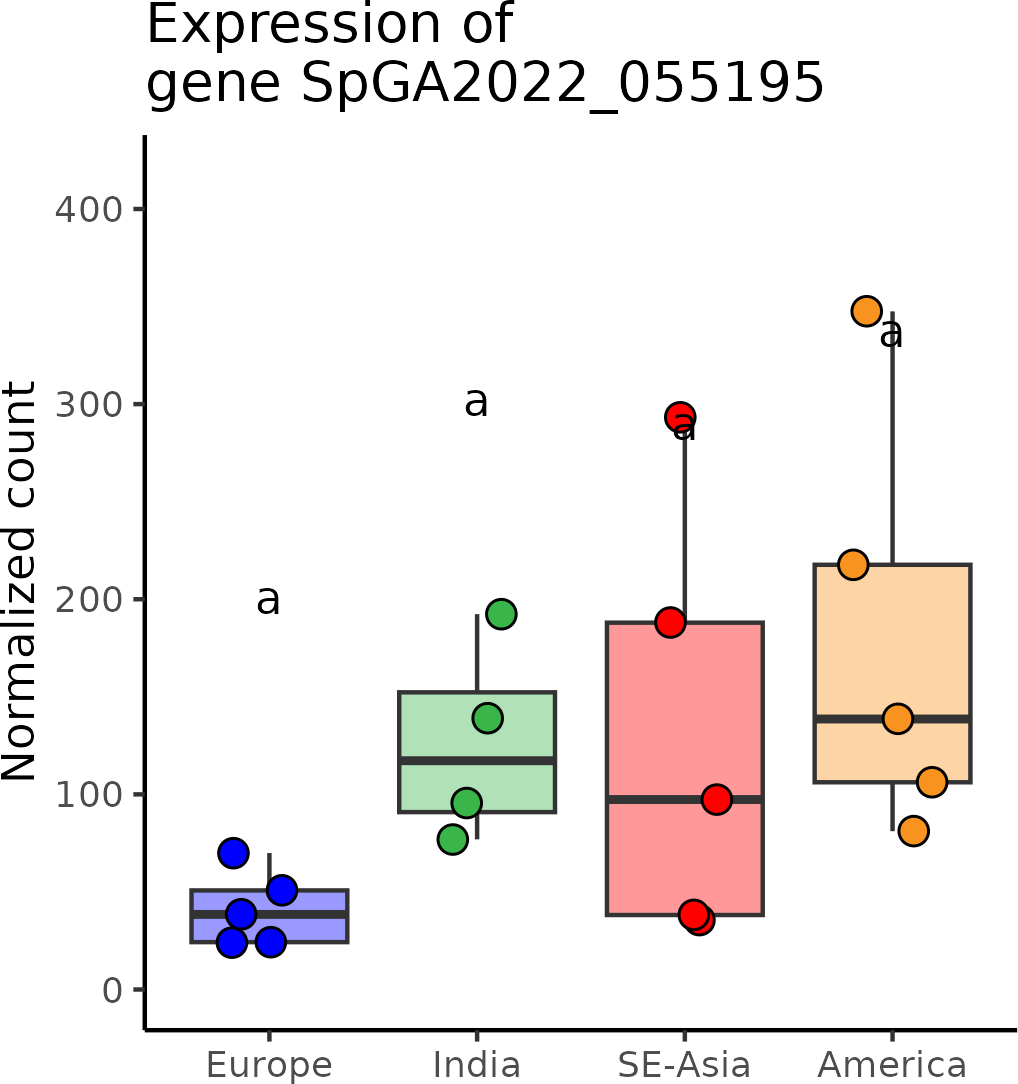


# SpGA2022_052378 (*DRMY1*)

**Putative function:** Protein of unknown function

***Arabidopsis* ortholog/homolog:** AT1G58220.1

**Alignment:**


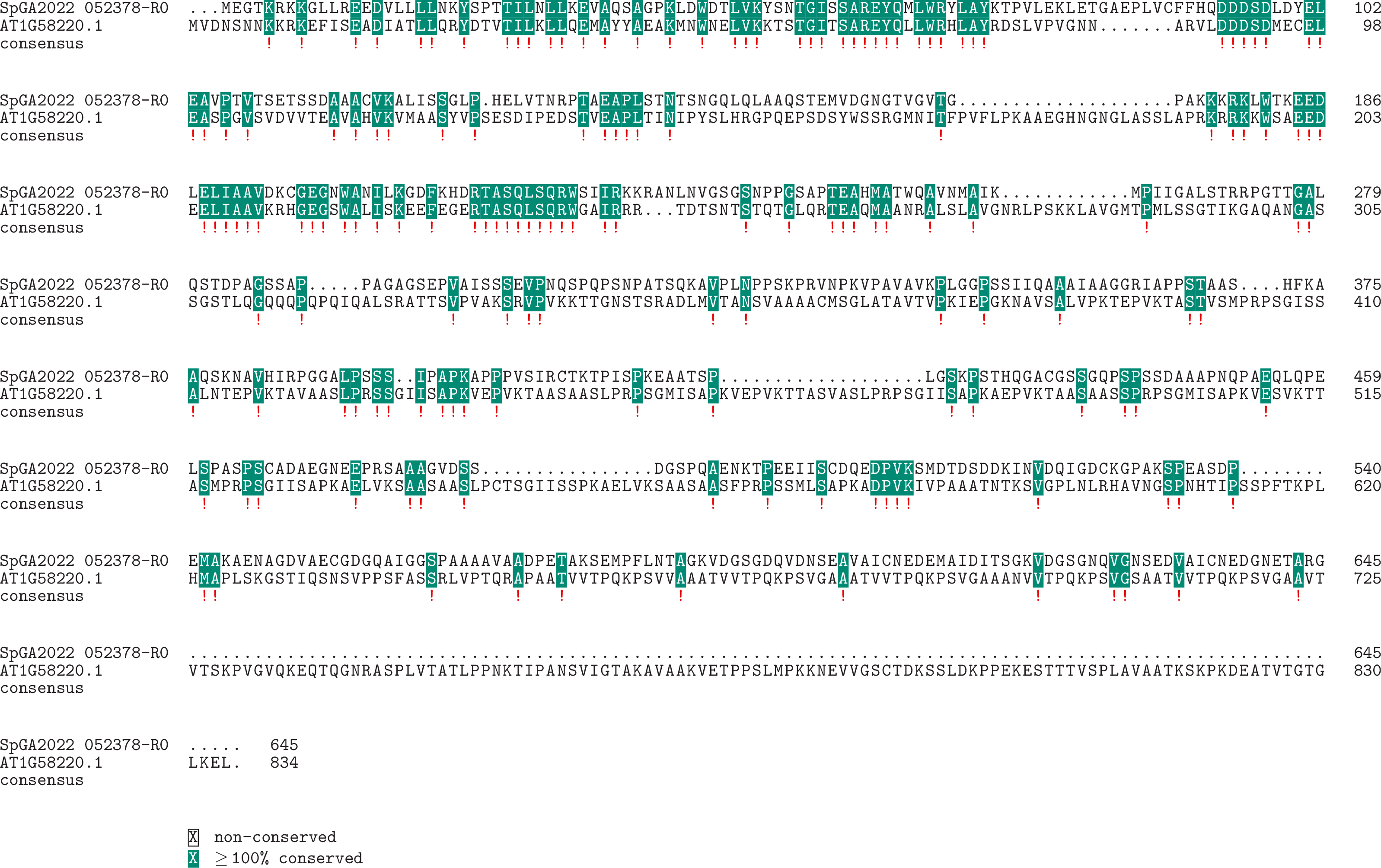


**Expression:**


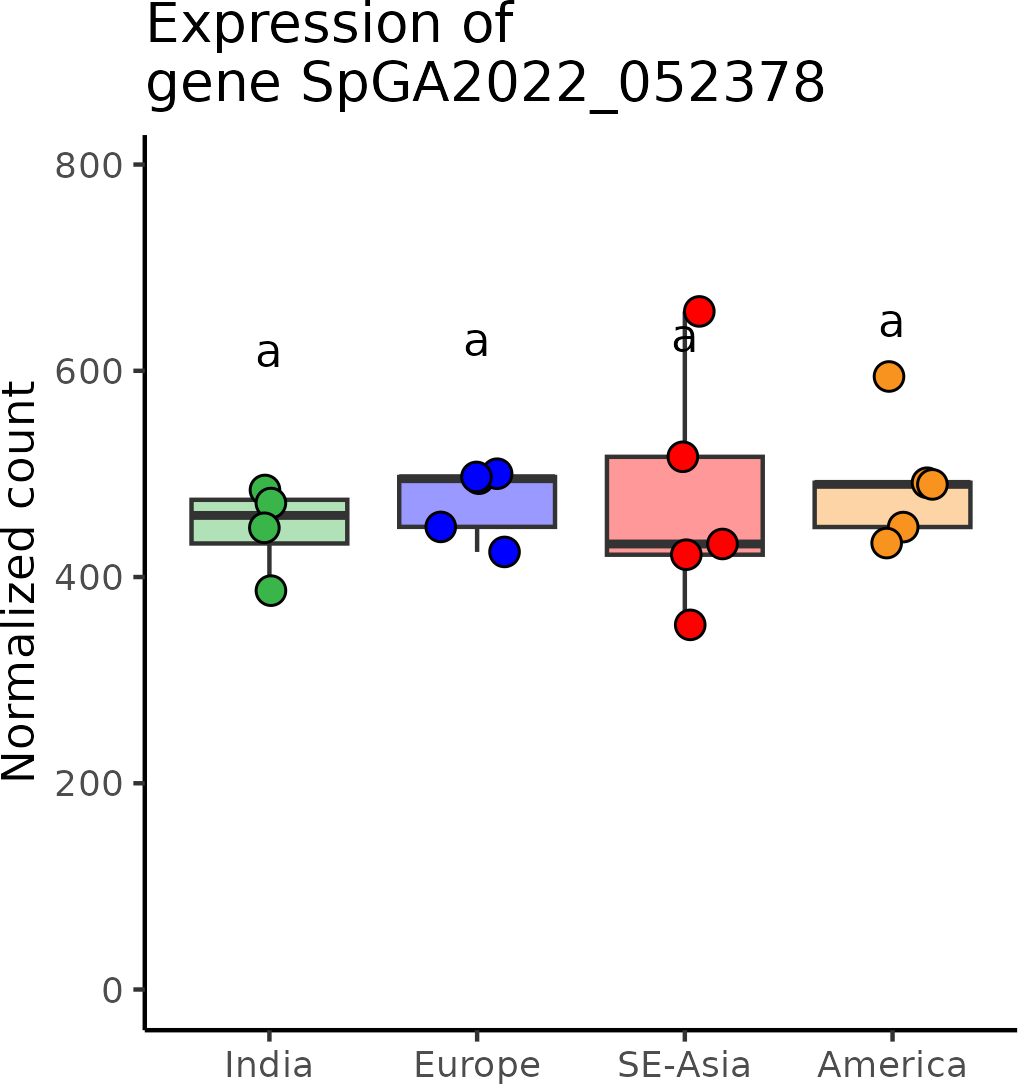


# SpGA2022_052156 (*EFOP3*)

**Putative function:** Similar to SRL2: Protein SEMI-ROLLED LEAF 2 (*Oryza sativa* subsp. japonica)

***Arabidopsis* ortholog/homolog:** AT1G05960.2

**Alignment:**


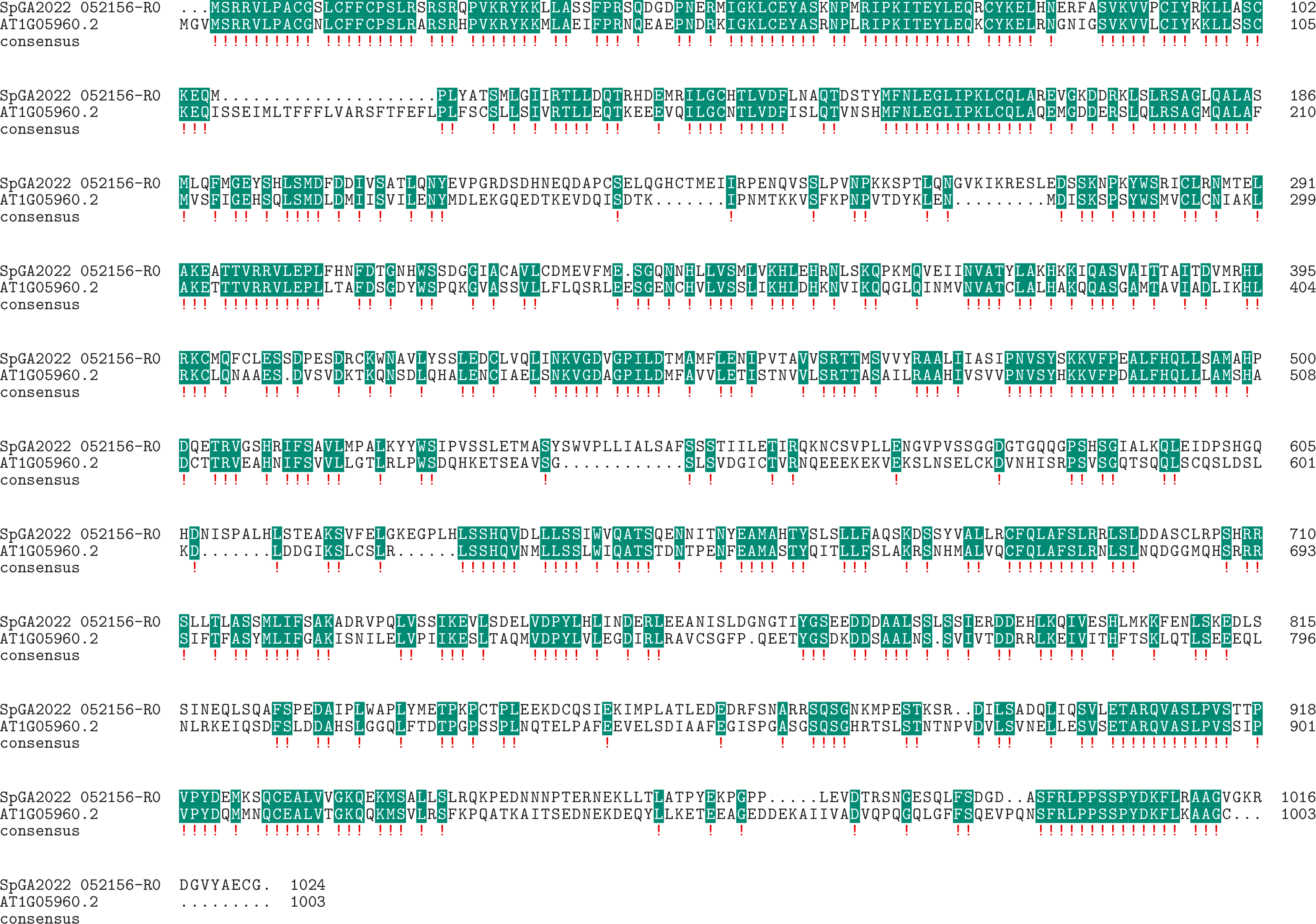


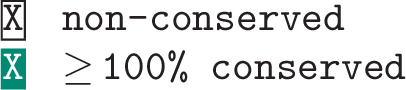


**Expression:**


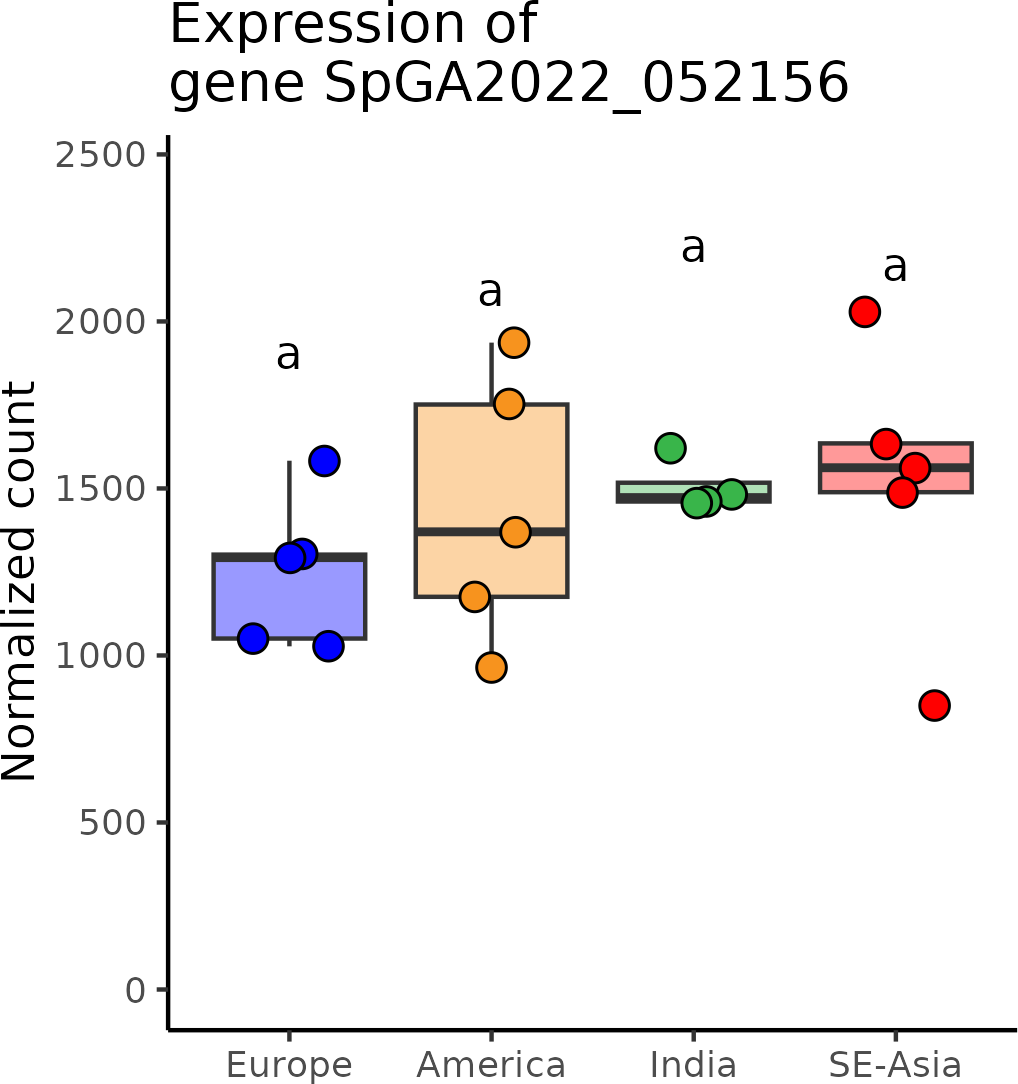


# SpGA2022_015101 (*ELMOD*)

**Putative function:** Similar to elmoA: ELMO domain-containing protein A (*Dictyostelium discoideum*)

***Arabidopsis* ortholog/homolog:** AT3G60260.1

**Alignment:**


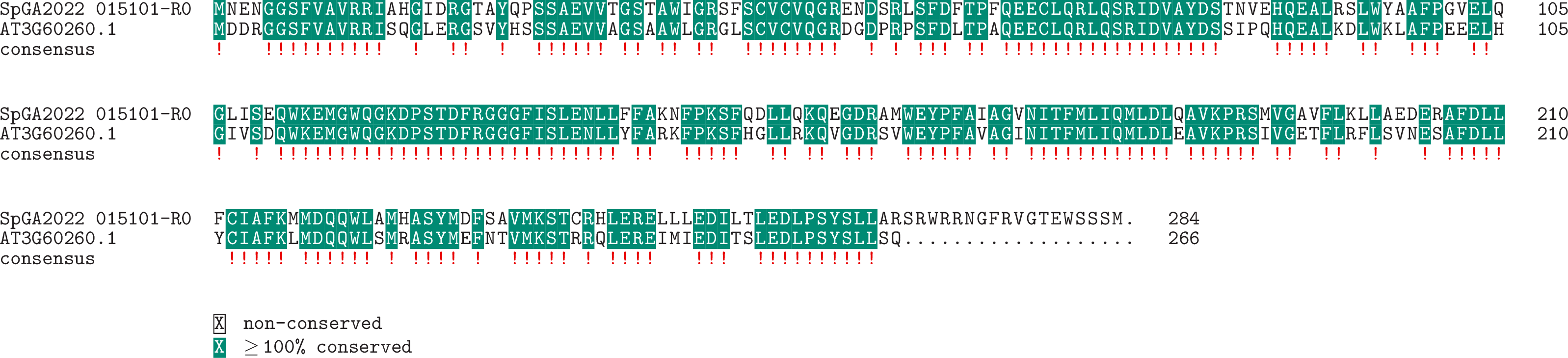


**Expression:**


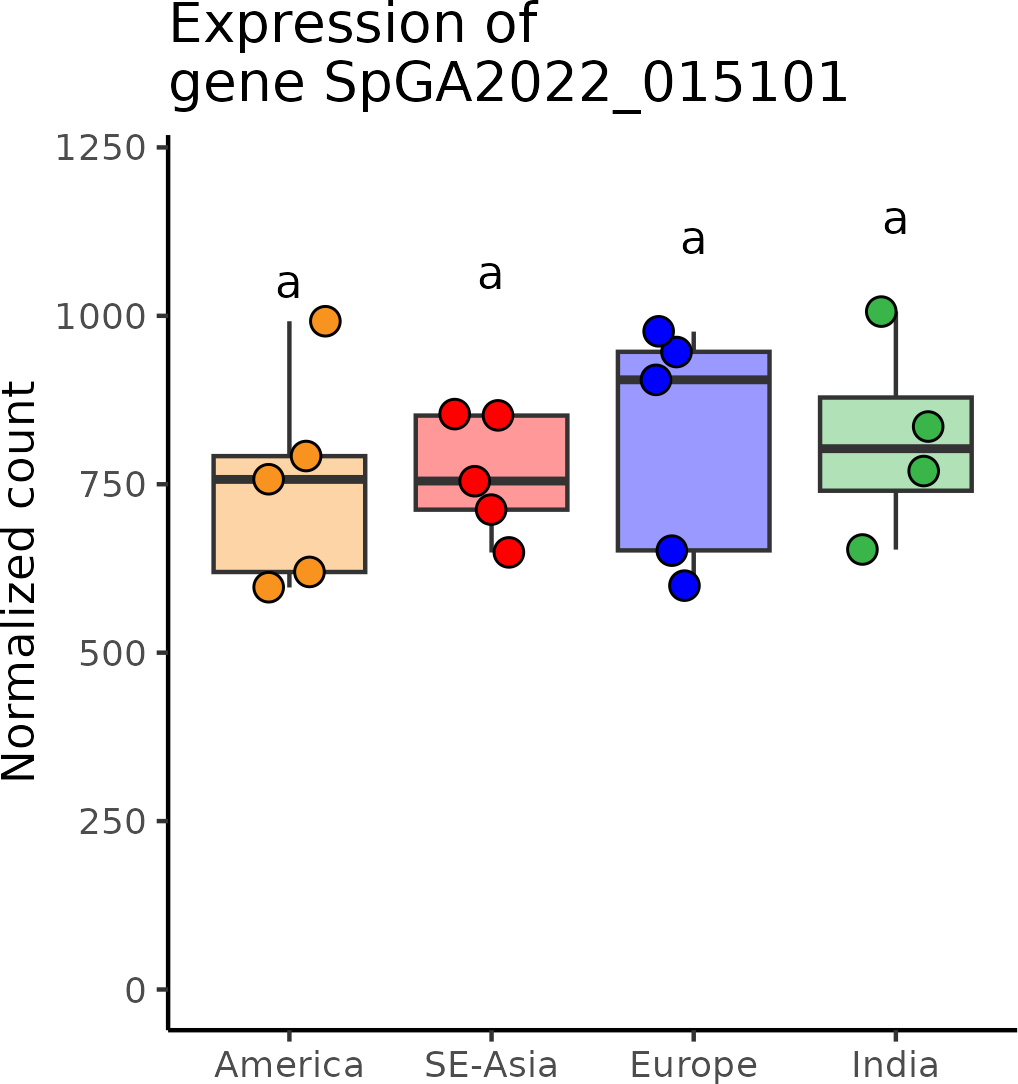


# SpGA2022_053158 (*FC1*)

**Putative function:** Similar to HEMH: Ferrochelatase-2

***Arabidopsis* ortholog/homolog:** AT5G26030.1

**Alignment:**


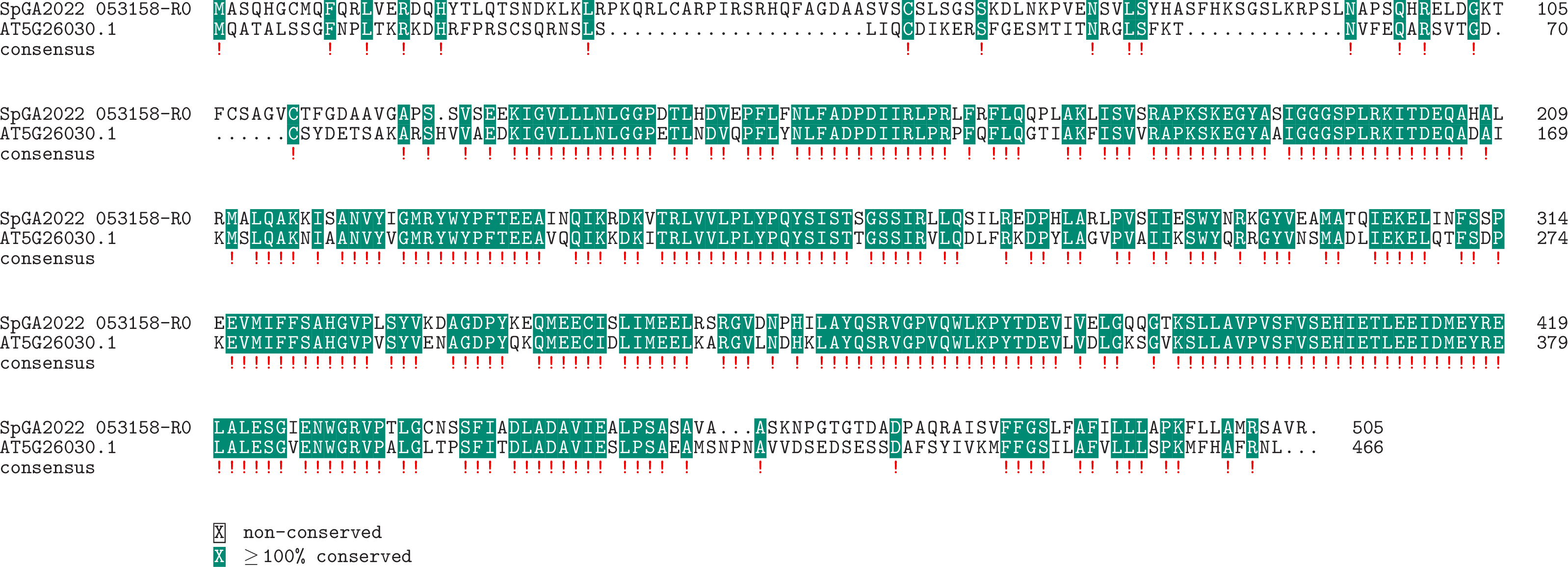


**Expression:**


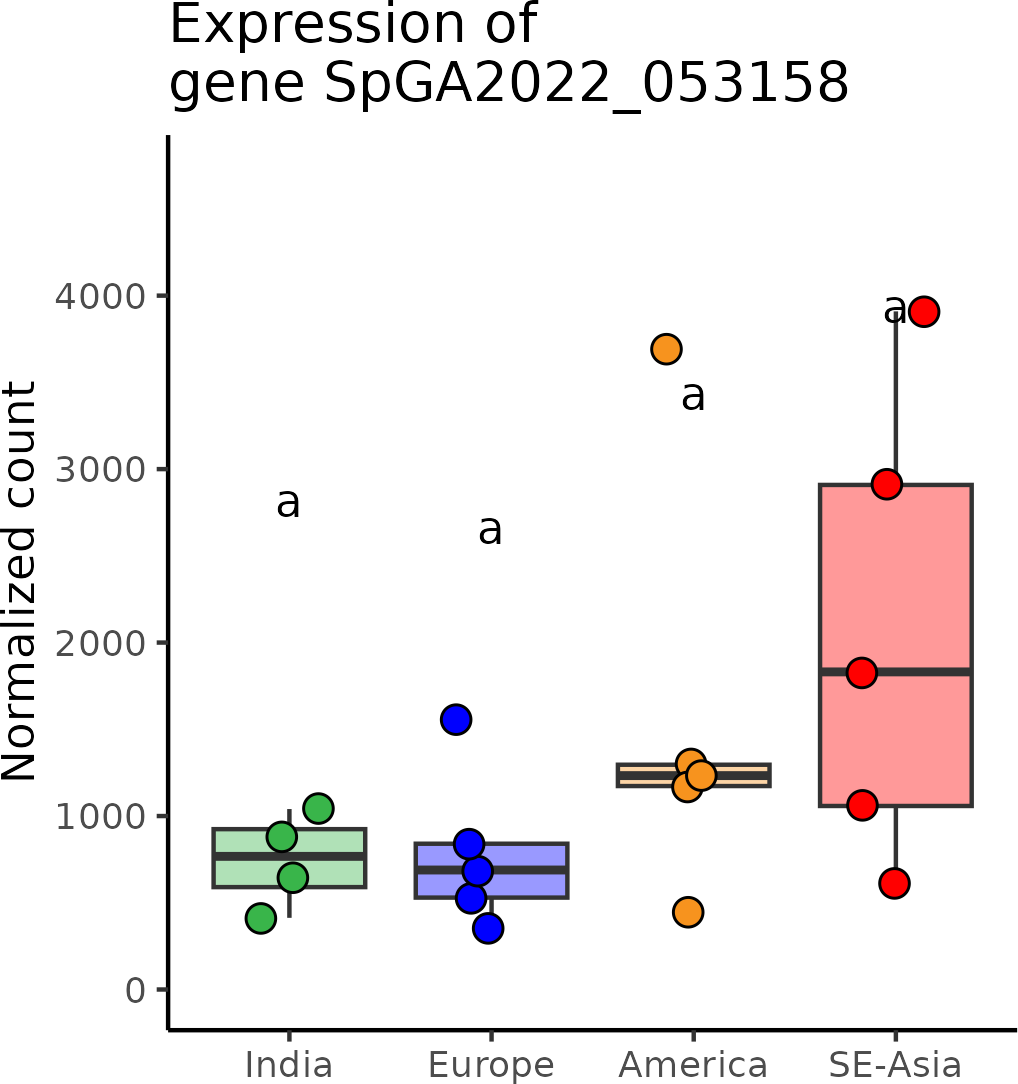


# SpGA2022_013448 (*FLK*)

**Putative function:** Similar to FLK: Flowering locus K homology domain (*Arabidopsis thaliana*)

***Arabidopsis* ortholog/homolog:** AT3G04610.1

**Alignment:**


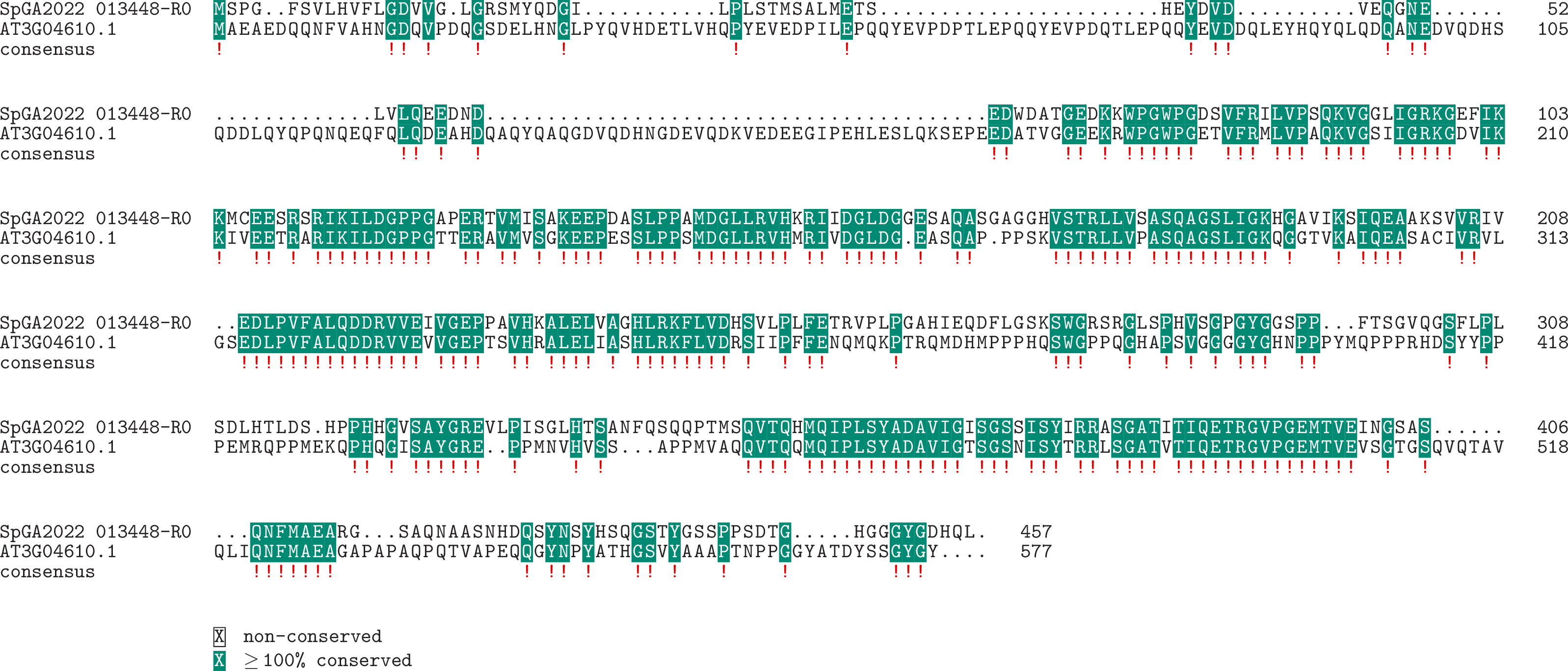


**Expression:**


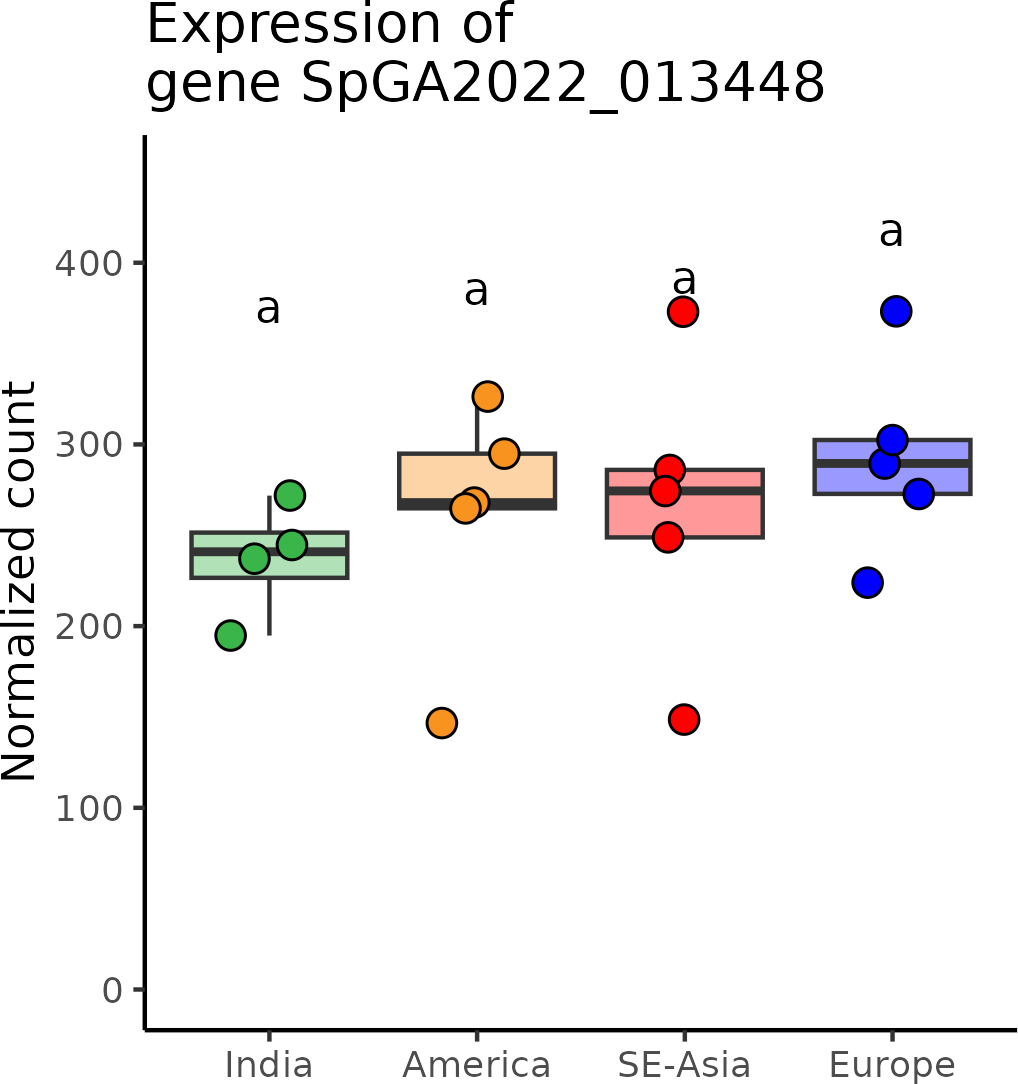


# SpGA2022_055227 (*GEND1*)

**Putative function:** Similar to PCMP-E19: Pentatricopeptide repeat-containing protein At2g33680 (*Arabidopsis thaliana*)

***Arabidopsis* ortholog/homolog:** AT2G33680.5

**Alignment:**


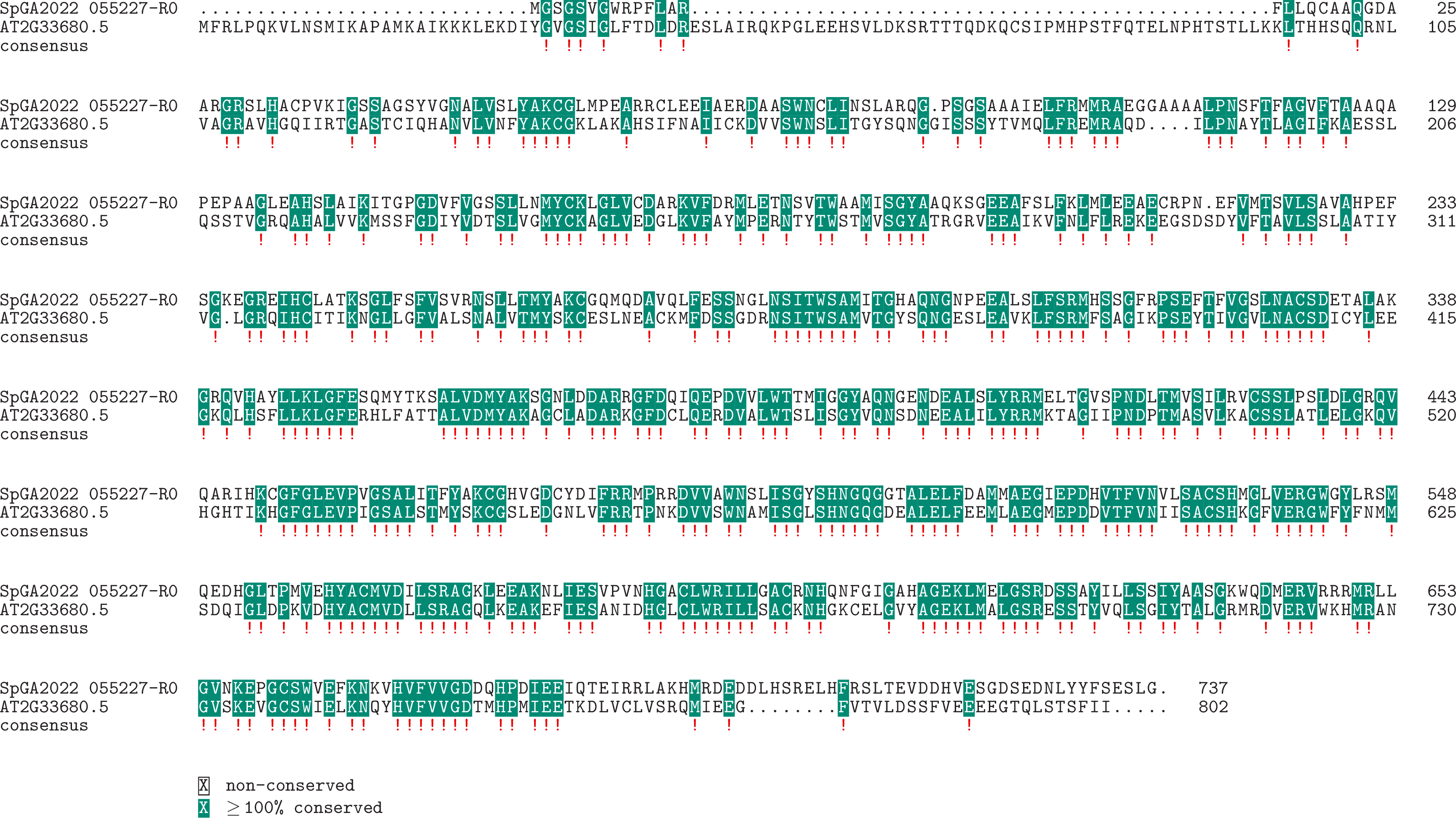


**Expression:**


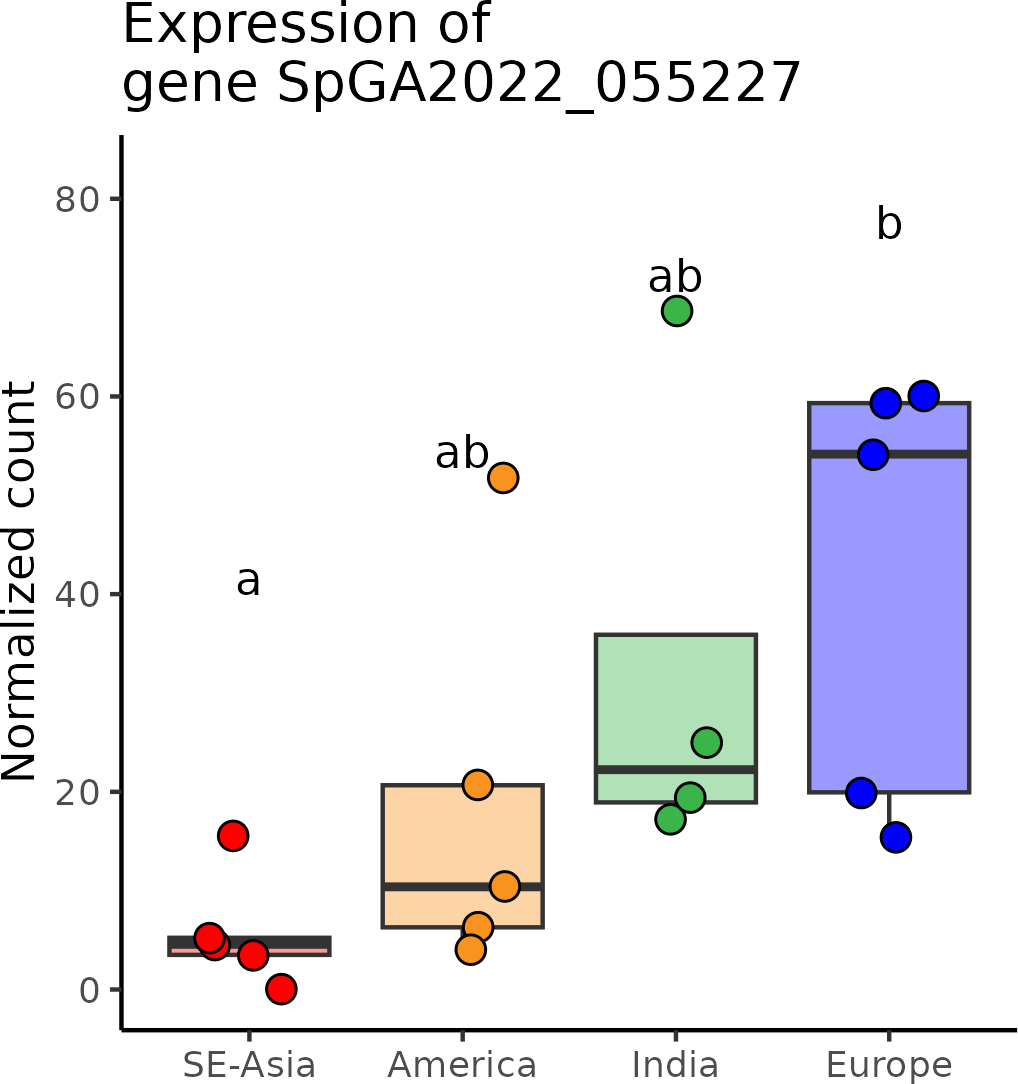


# SpGA2022_010691 (*NF-Y_AT2G27470*)

**Putative function:** Protein of unknown function

***Arabidopsis* ortholog/homolog:** AT2G27470.1

**Alignment:**


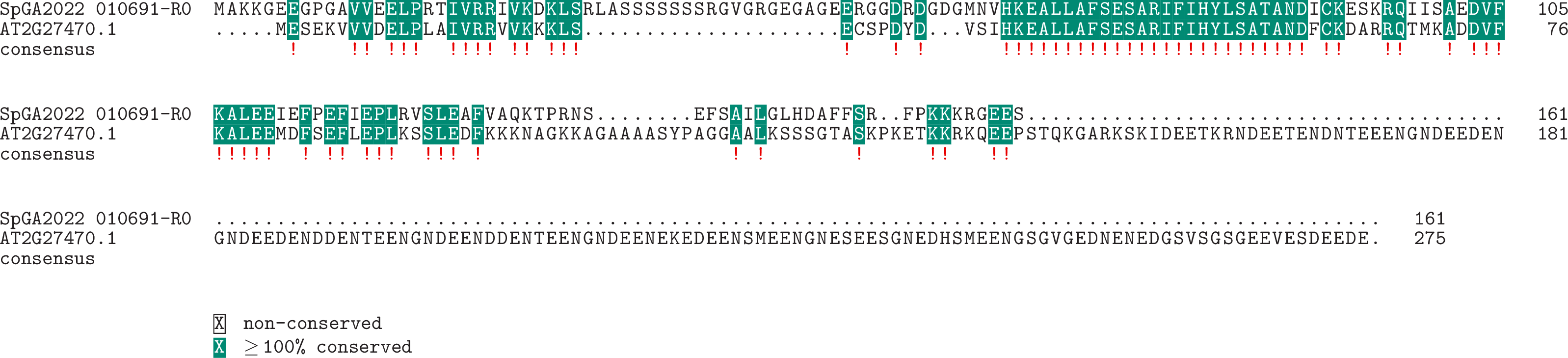


**Expression:**


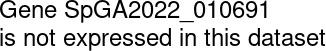


# SpGA2022_005107 (*NOTCHLESS*)

**Putative function:** Similar to NLE1: Notchless protein homolog (*Arabidopsis thaliana*)

***Arabidopsis* ortholog/homolog:** AT5G52820.1

**Alignment:**


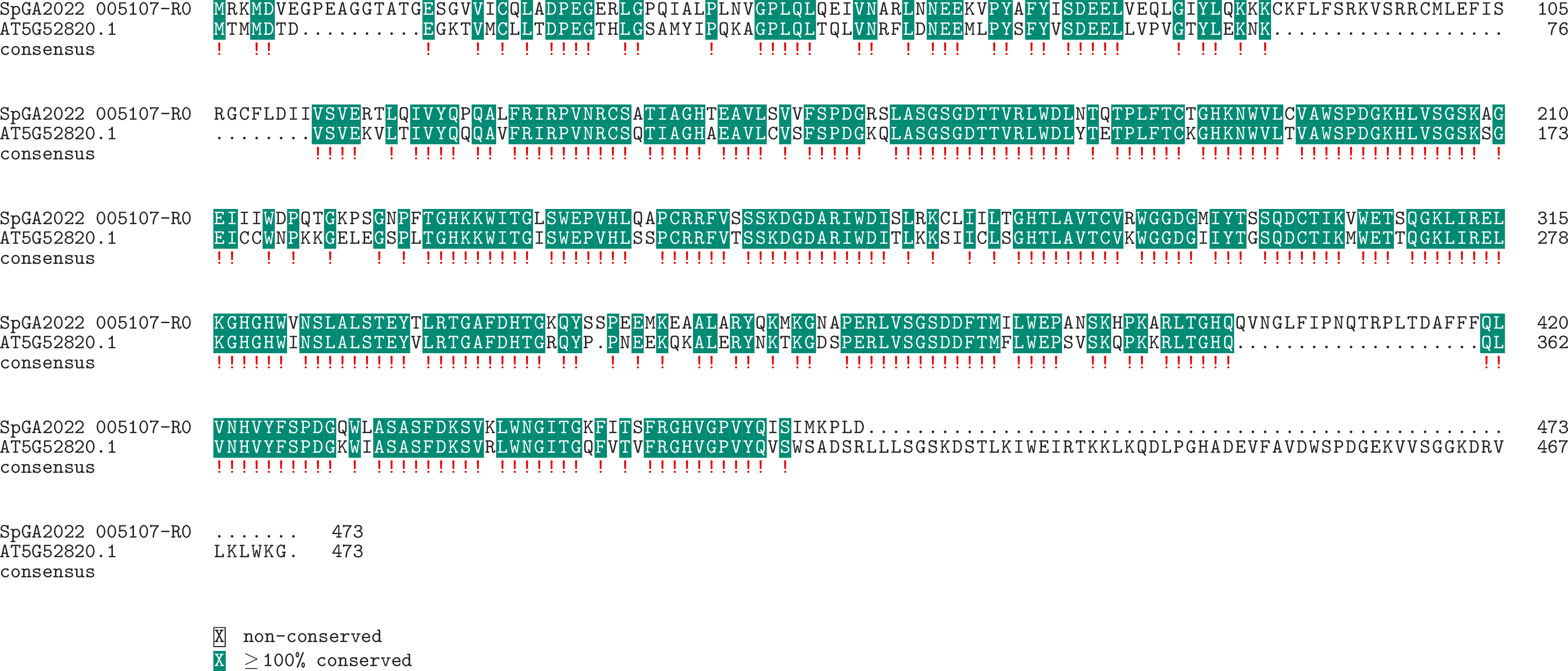


**Expression:**


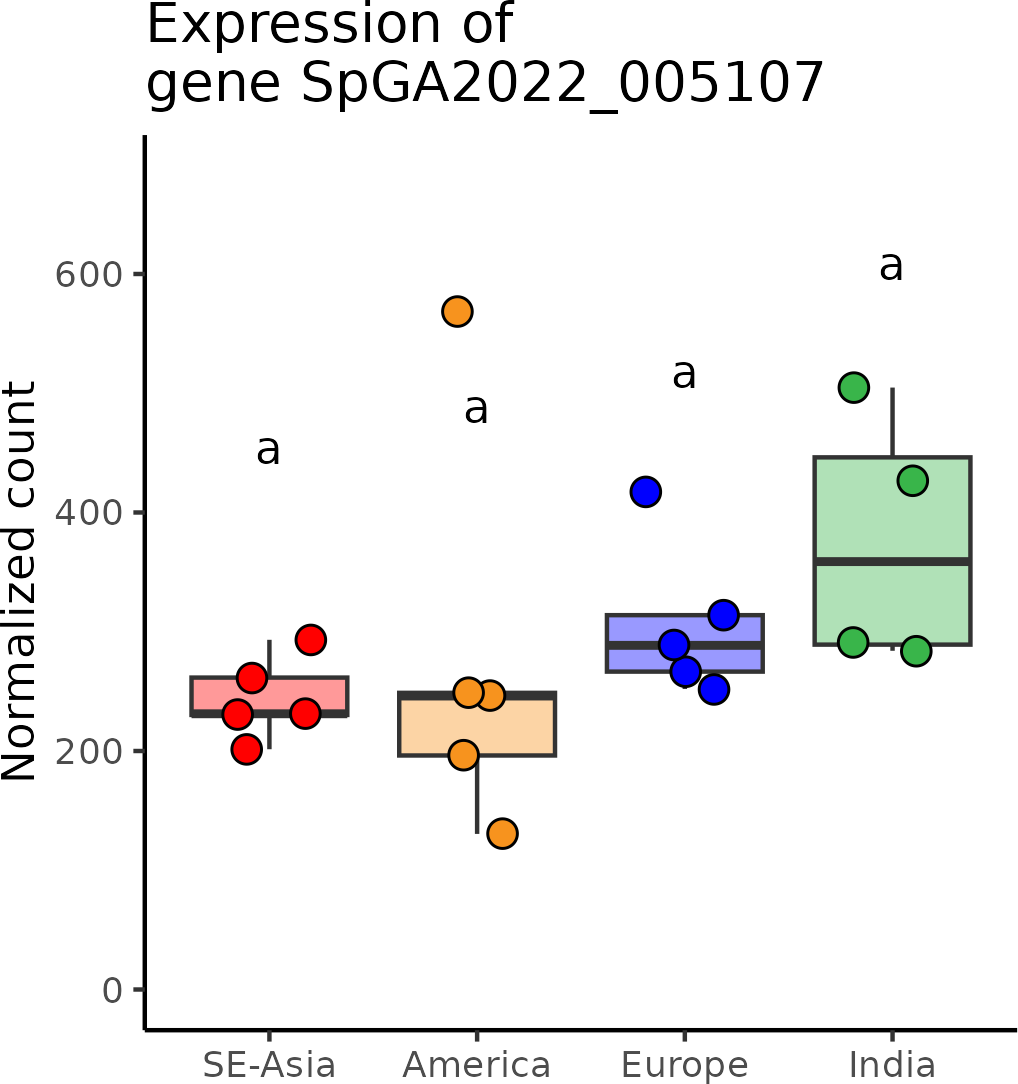


# SpGA2022_054699 (*NUDT2*)

**Putative function:** Similar to NUDT2: Nudix hydrolase 2 (*Arabidopsis thaliana*)

***Arabidopsis* ortholog/homolog:** AT5G47650.2

**Alignment:**


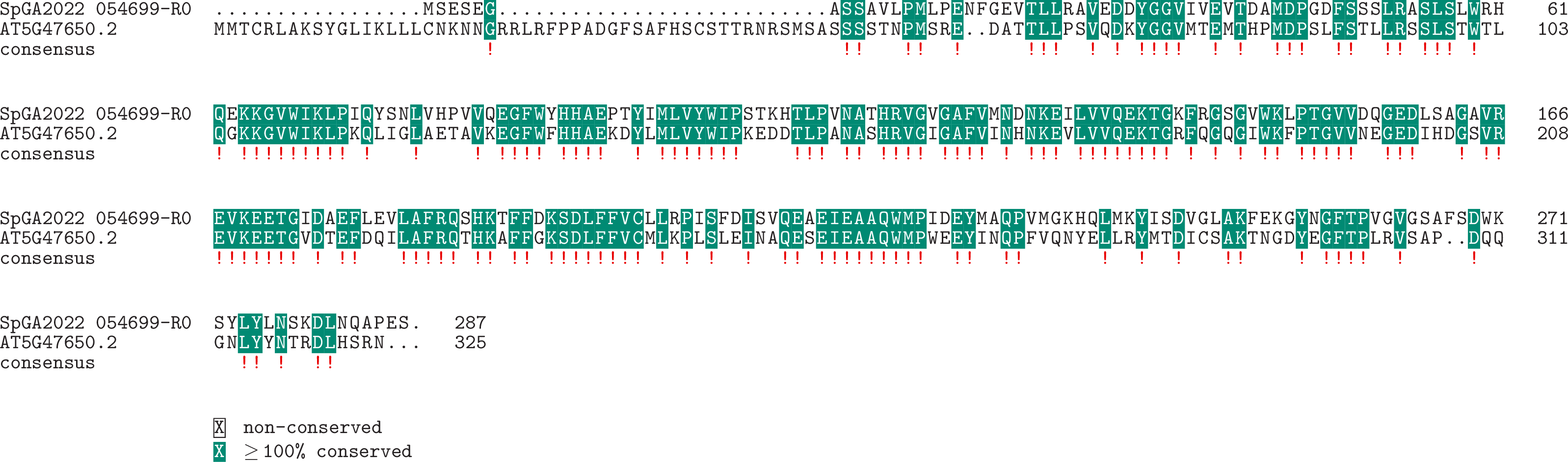


**Expression:**


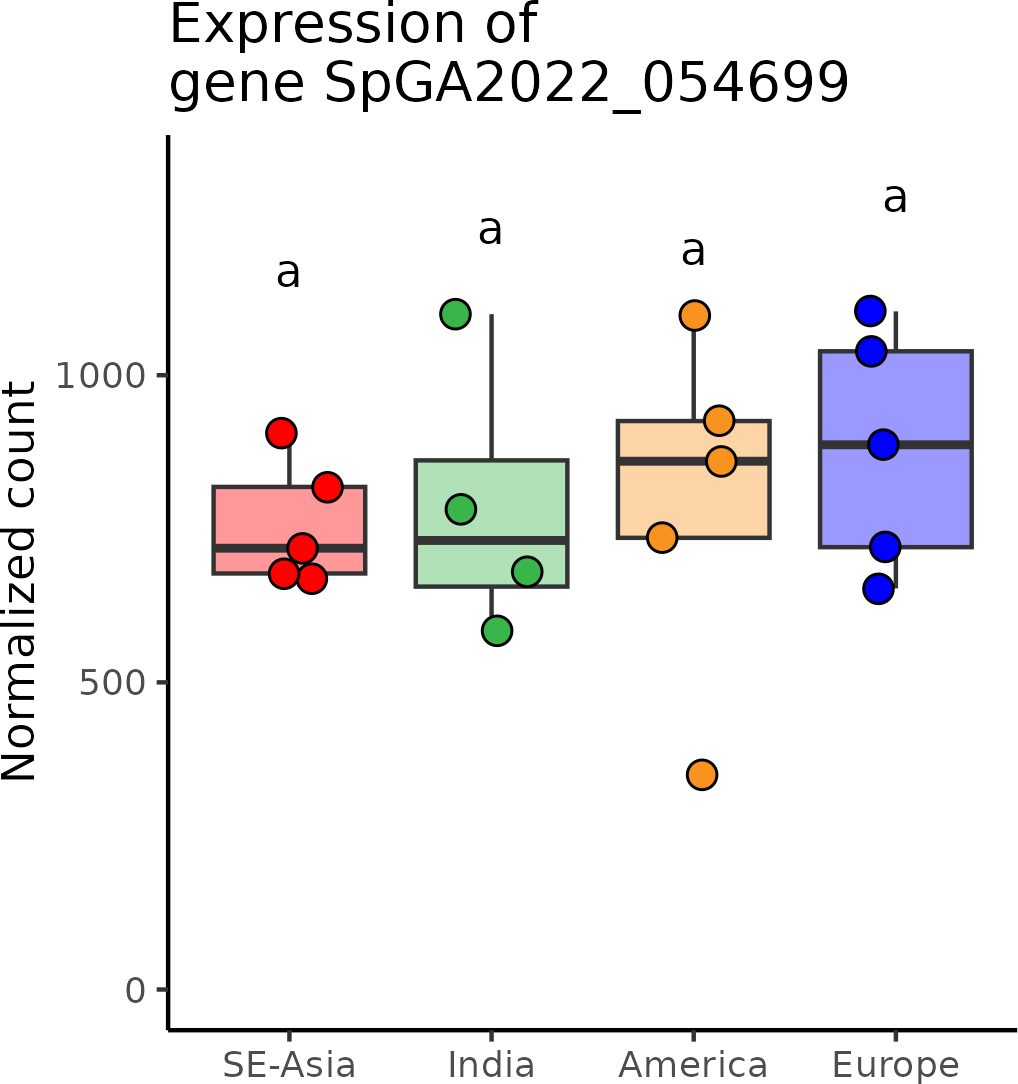


# SpGA2022_002887 (*NUP214*)

**Putative function:** Similar to NUP214: Nuclear pore complex protein NUP214 (*Arabidopsis thaliana*) Similar to NUP214: Nuclear pore complex protein NUP214 (*Arabidopsis thaliana*) ***Arabidopsis* ortholog/homolog:** AT1G55540.2

**Alignment:**


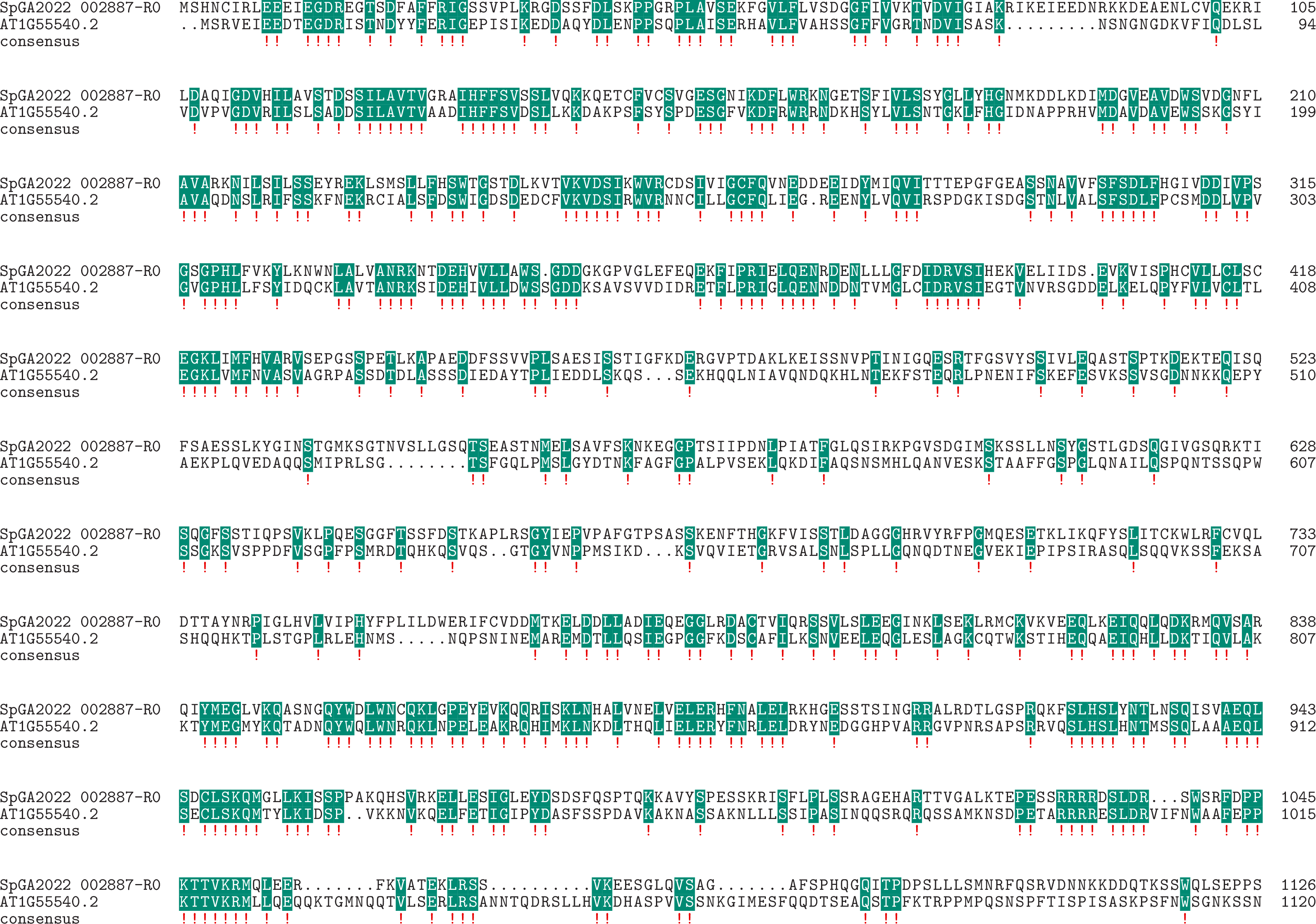


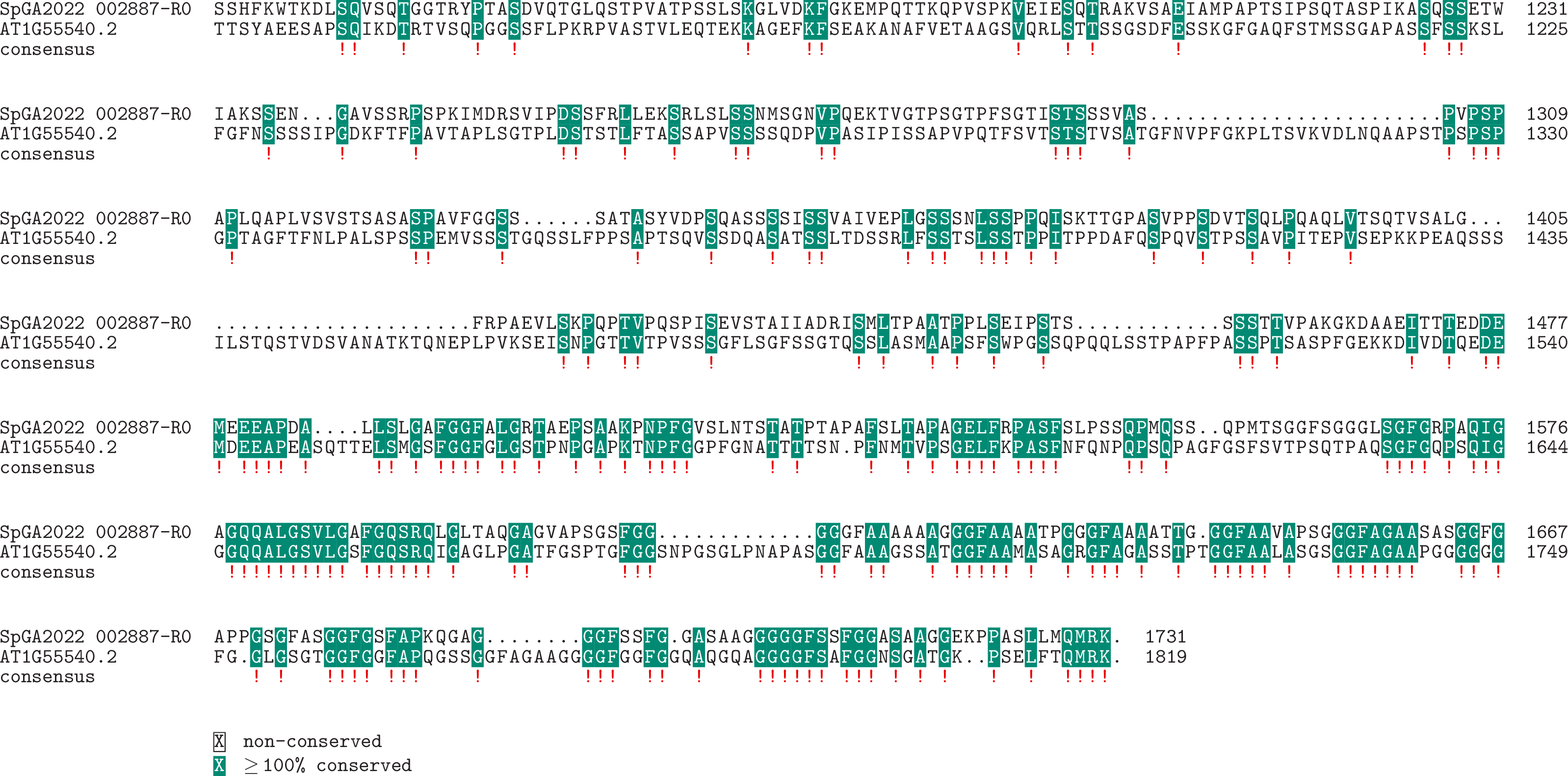


**Expression:**


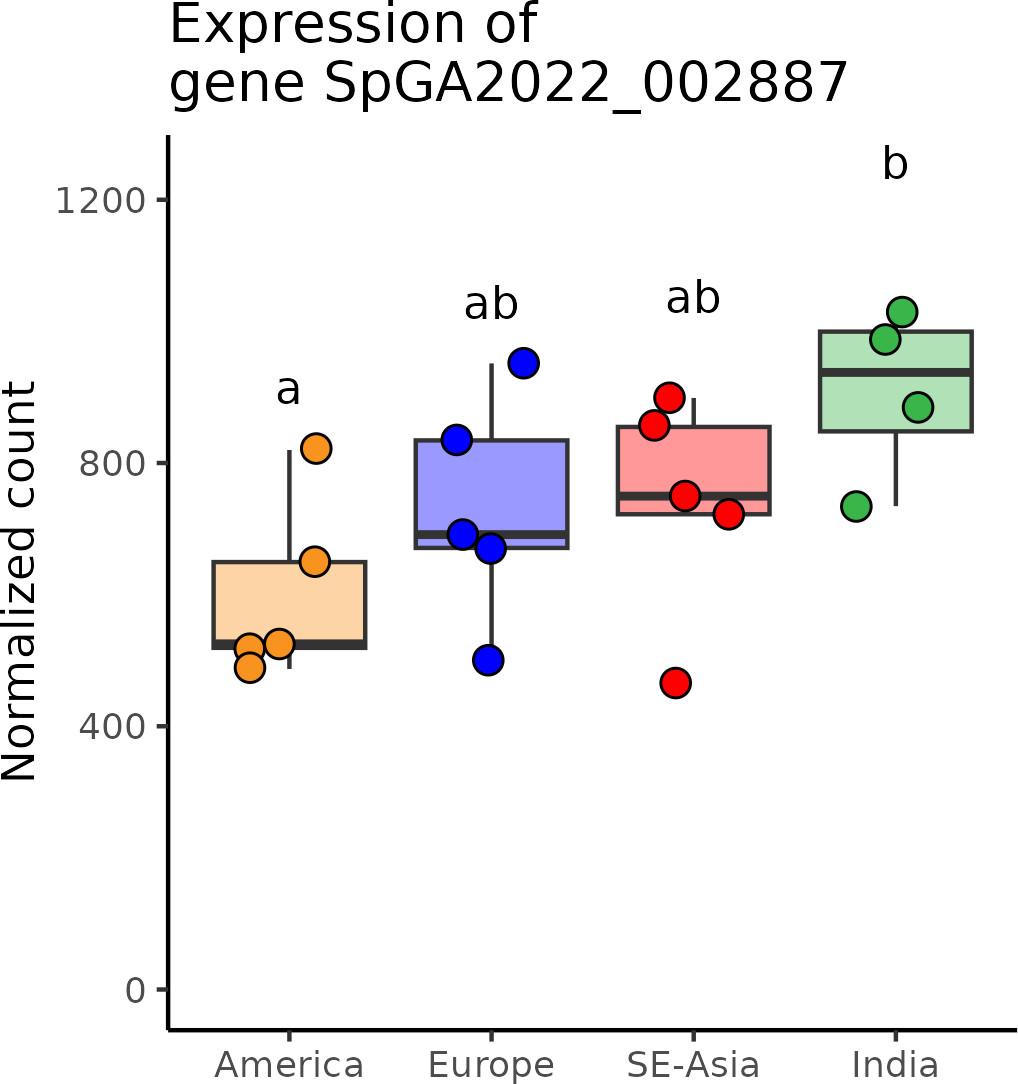


# SpGA2022_054509 (*PHABULOSA/PHAVOLUTA*)

**Putative function:** Similar to HOX32: Homeobox-leucine zipper protein HOX32 (*Oryza sativa* subsp. japonica)

***Arabidopsis* ortholog/homolog:** AT2G34710.1

**Alignment:**


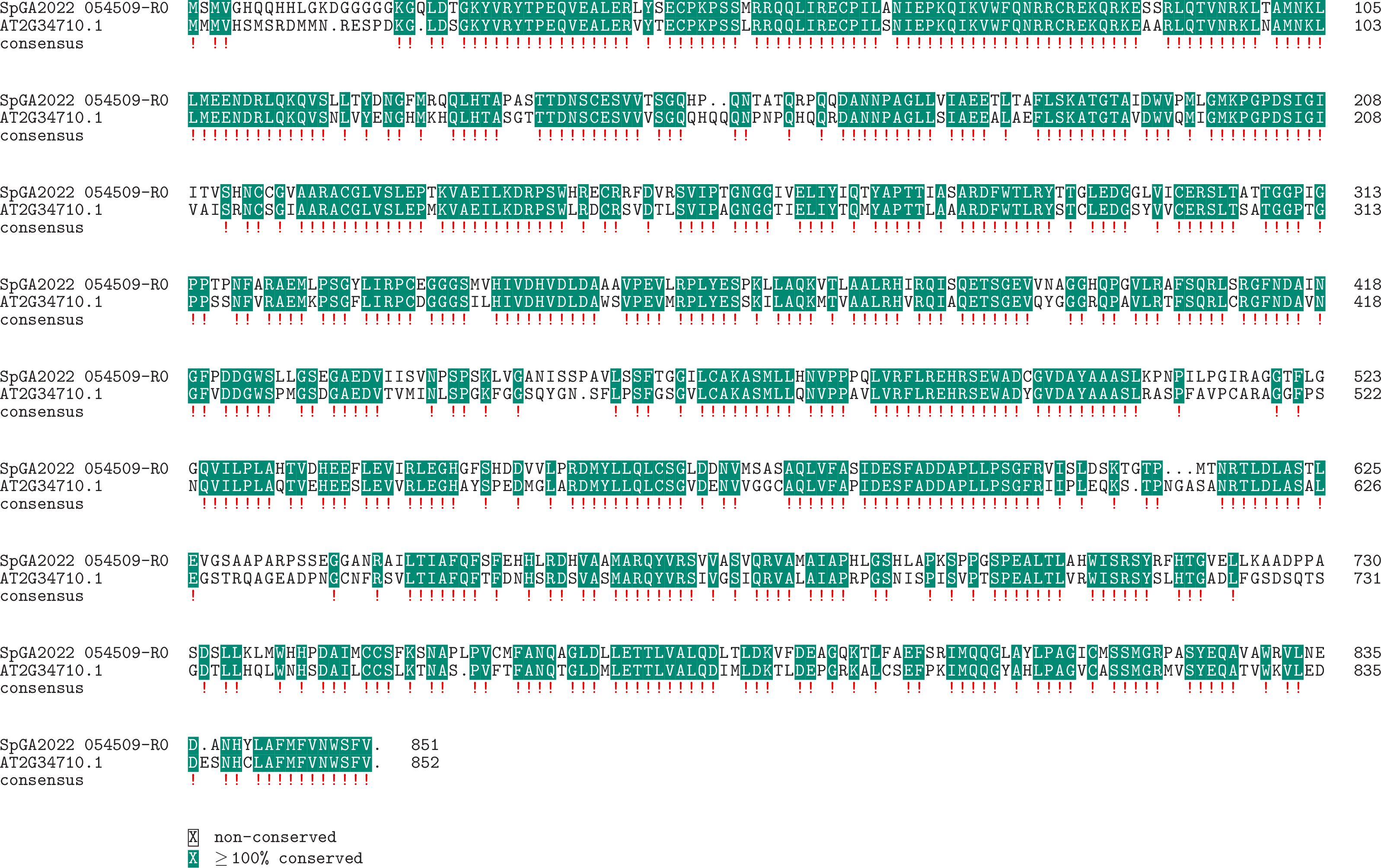


**Expression:**


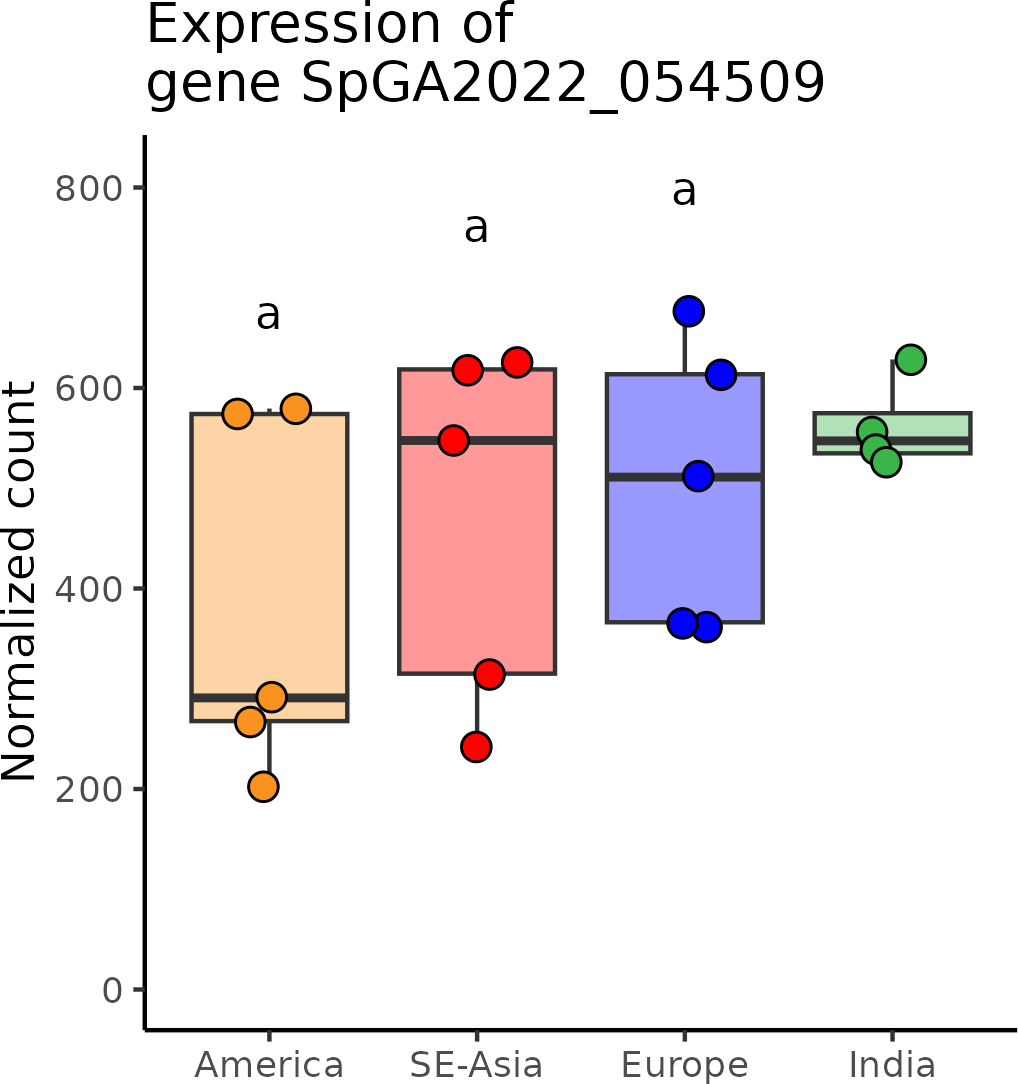


# SpGA2022_055362 (*PPP*)

**Putative function:** Similar to Ppp6r2: Serine/threonine-protein phosphatase 6 regulatory subunit 2 (*Mus musculus*)

***Arabidopsis* ortholog/homolog:** AT1G07990.1

**Alignment:**


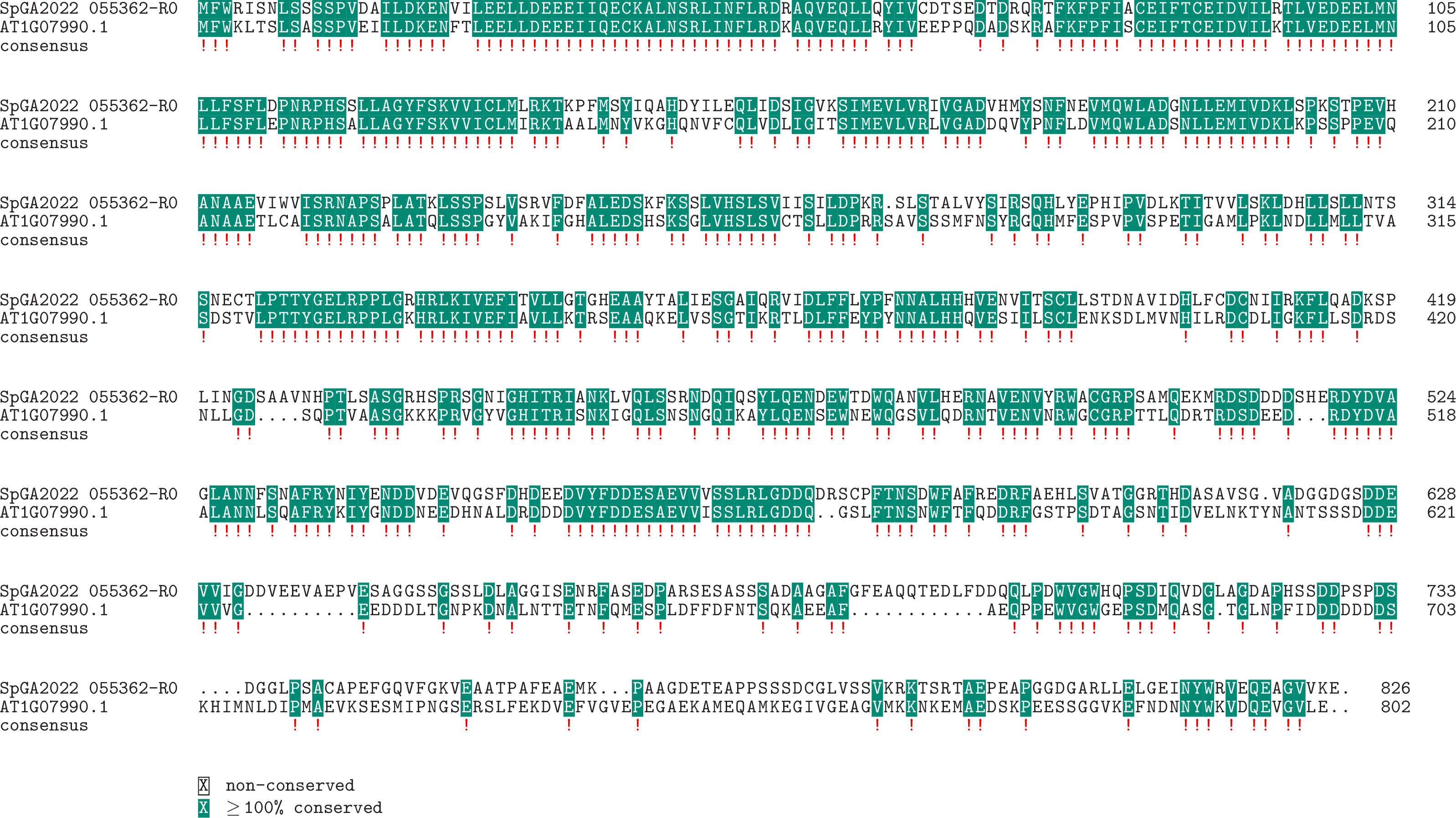


**Expression:**


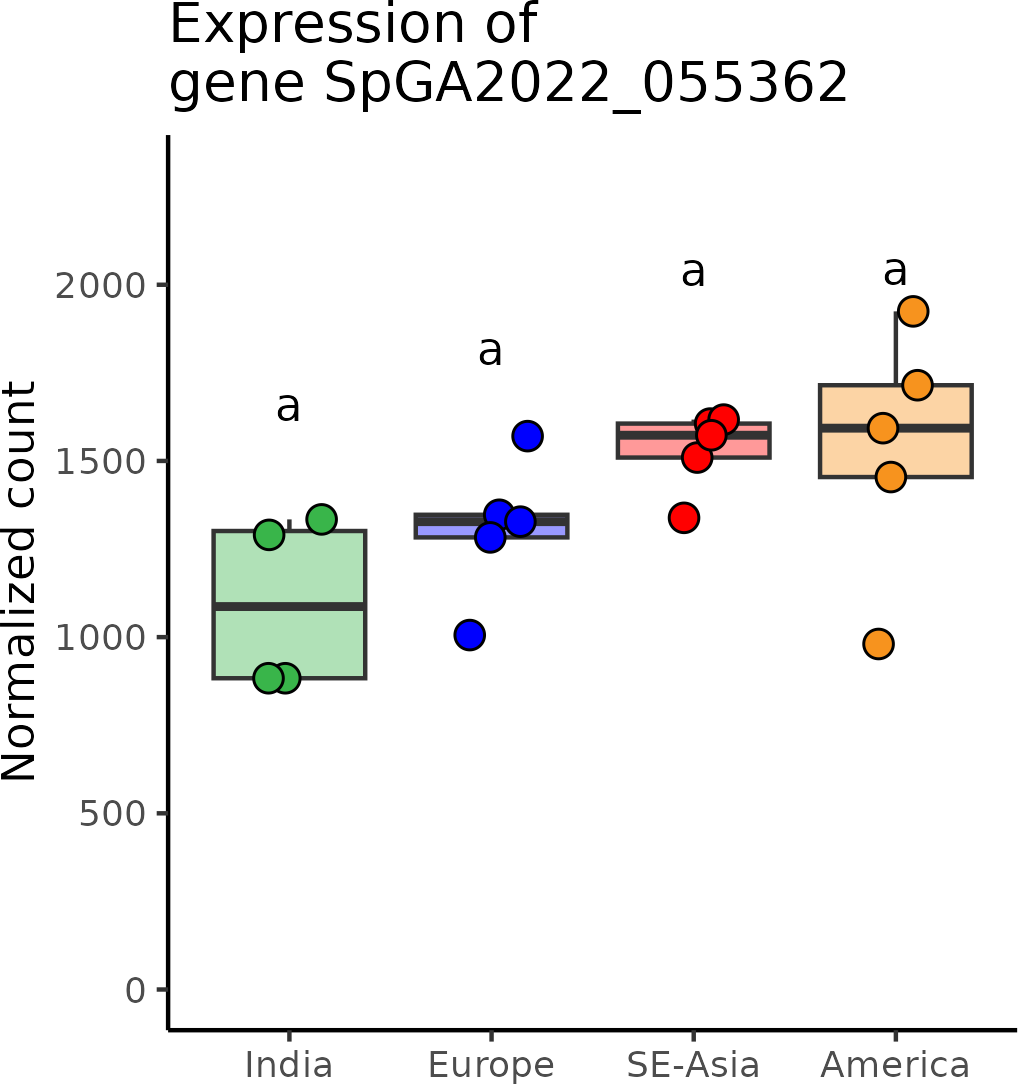


# SpGA2022_014906 (*RabGAP*)

**Putative function:** Protein of unknown function

***Arabidopsis* ortholog/homolog:** AT5G52580.2

**Alignment:**


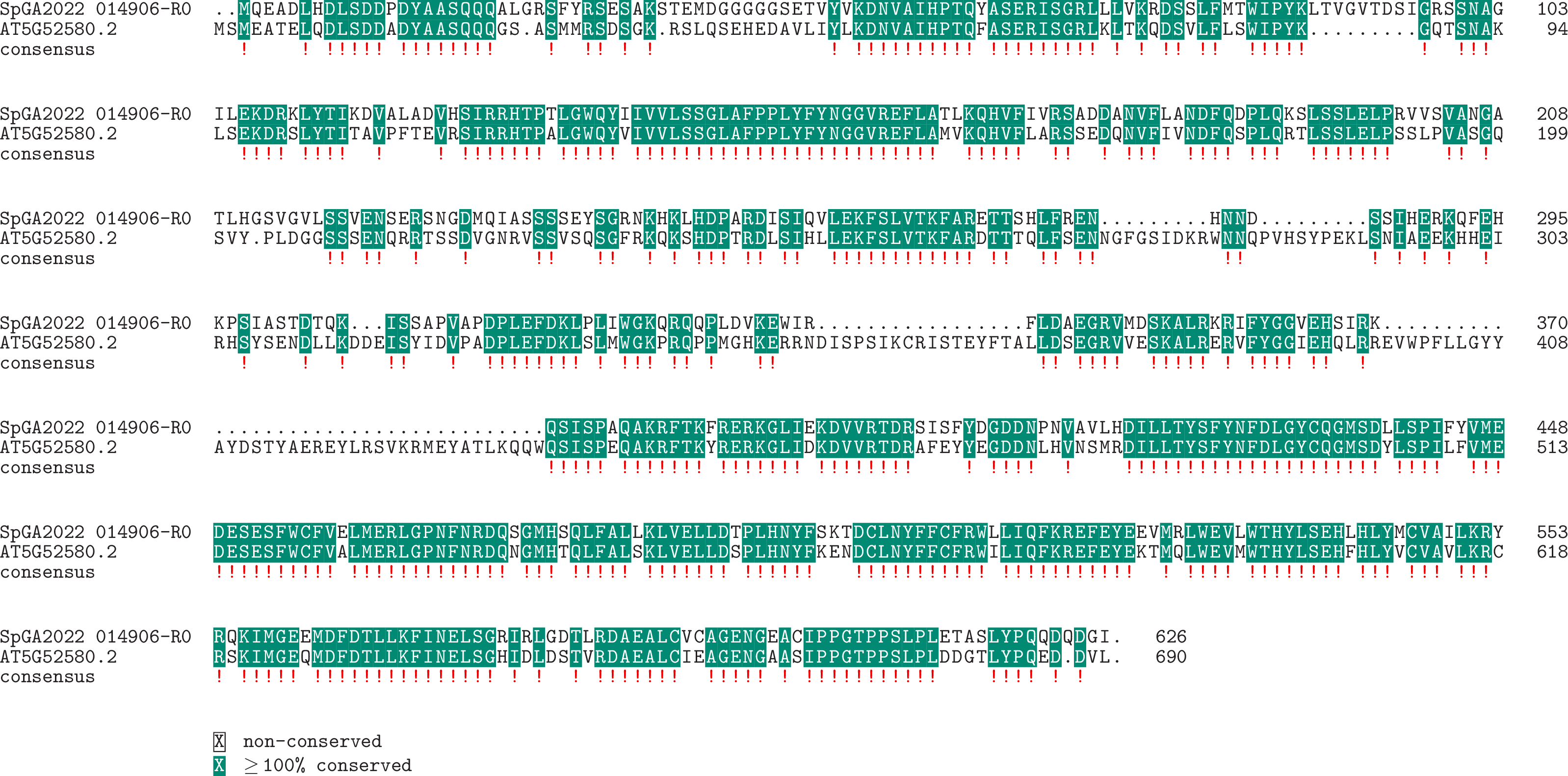


**Expression:**


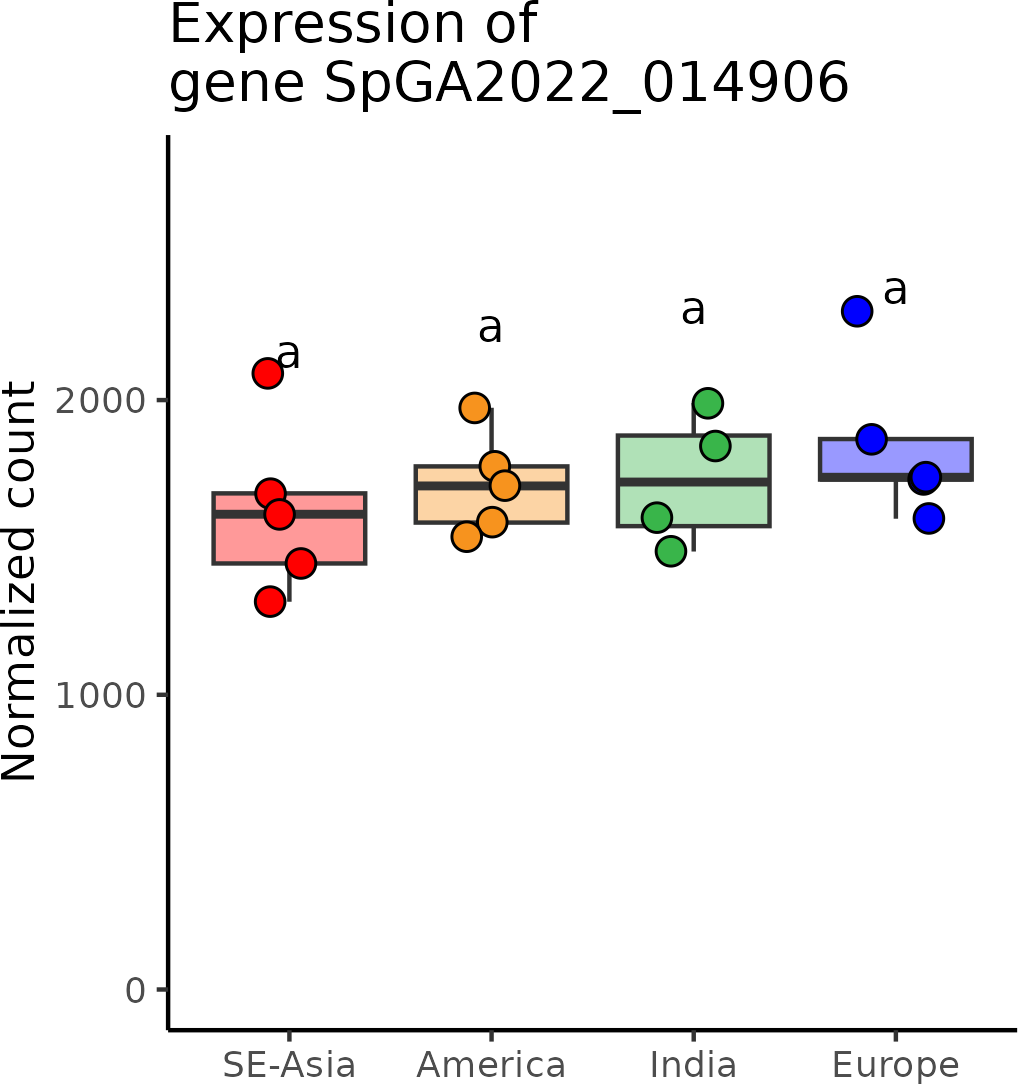


# SpGA2022_007853 (*RbgA*)

**Putative function:** Similar to DGP3: DAR GTPase 3

***Arabidopsis* ortholog/homolog:** AT4G02790.1

**Alignment:**


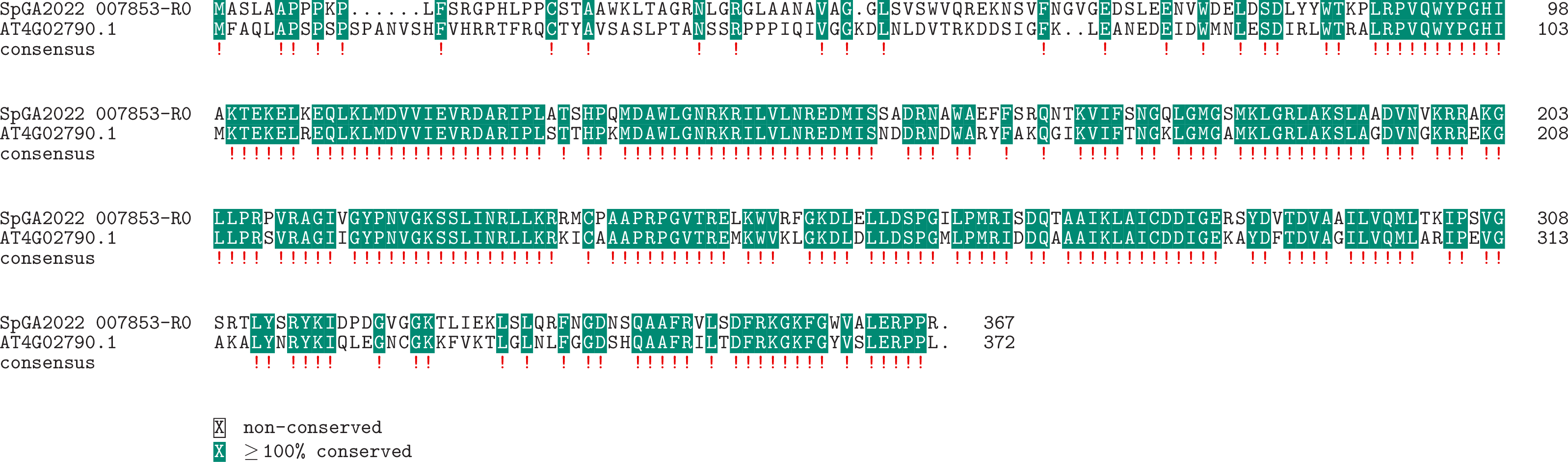


**Expression:**


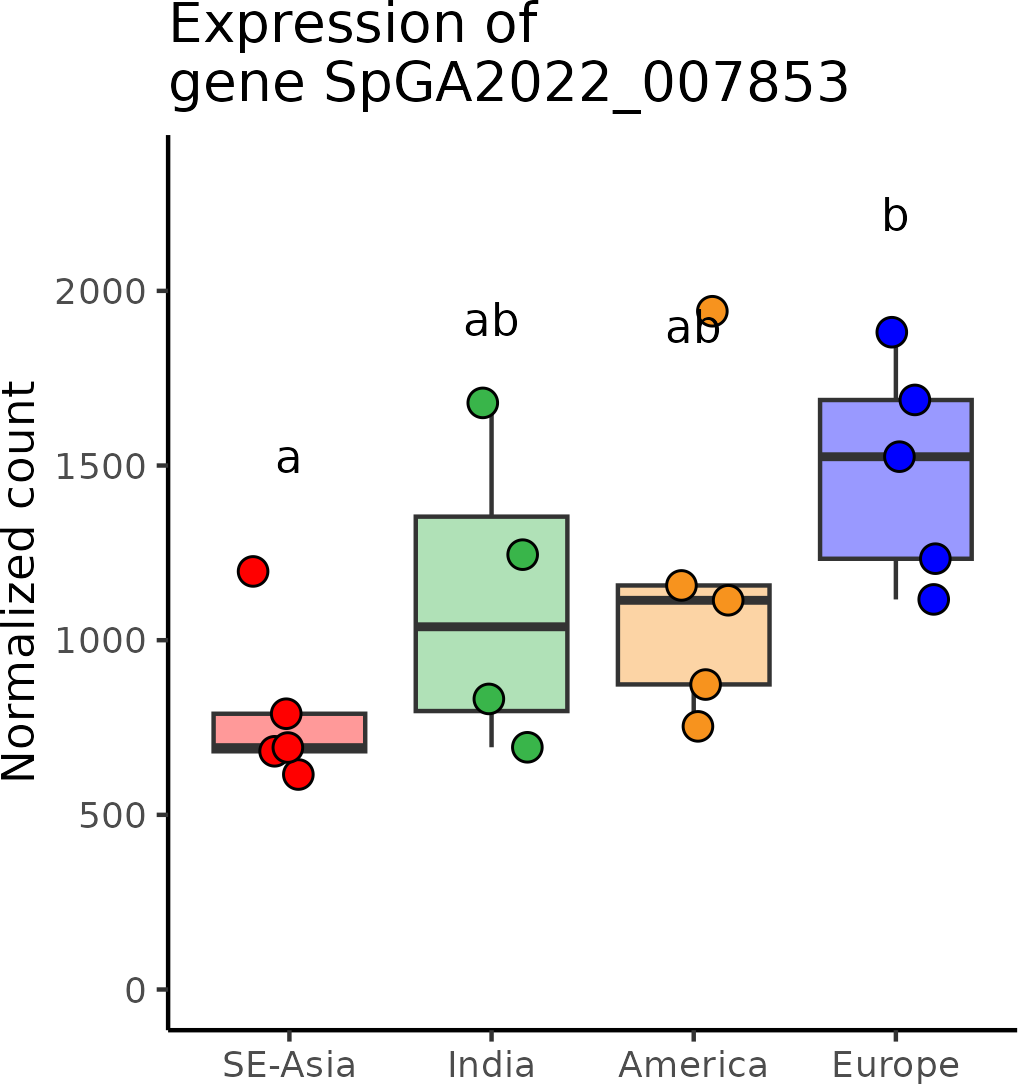


# SpGA2022_052273 (*SECA2*)

**Putative function:** Similar to SECA2: Protein translocase subunit SECA2

***Arabidopsis* ortholog/homolog:** AT1G21650.3

**Alignment:**


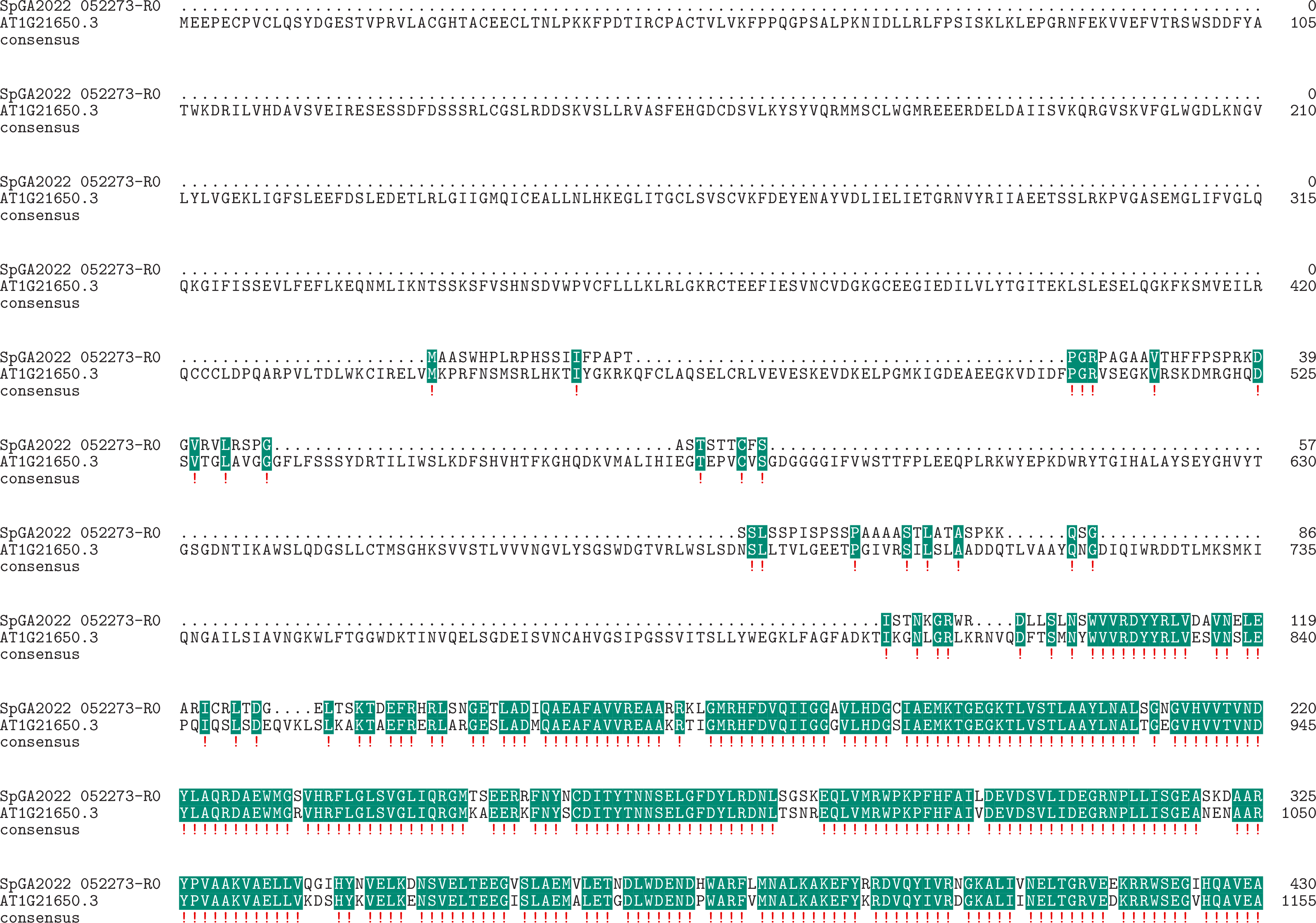


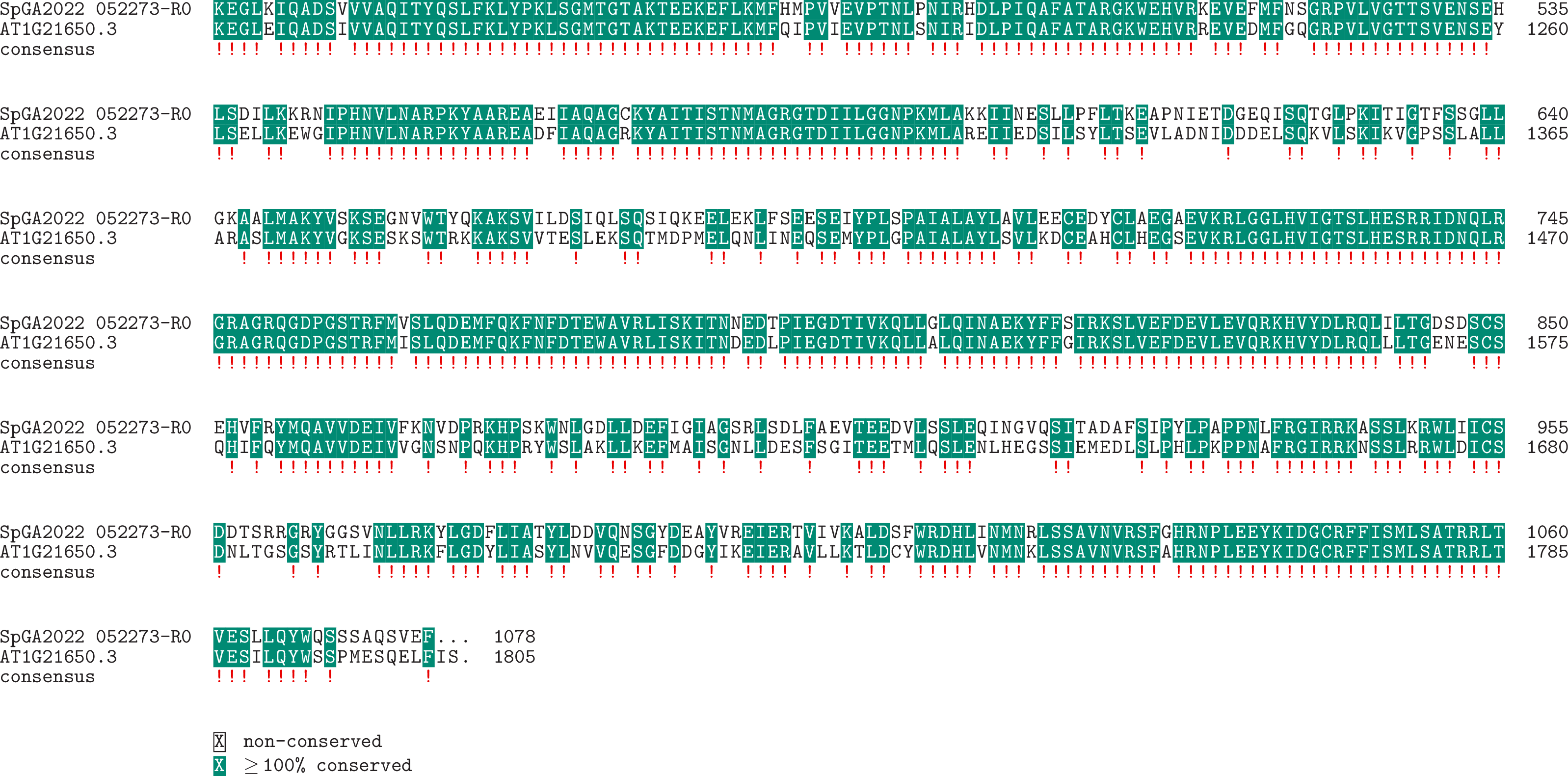


**Expression:**


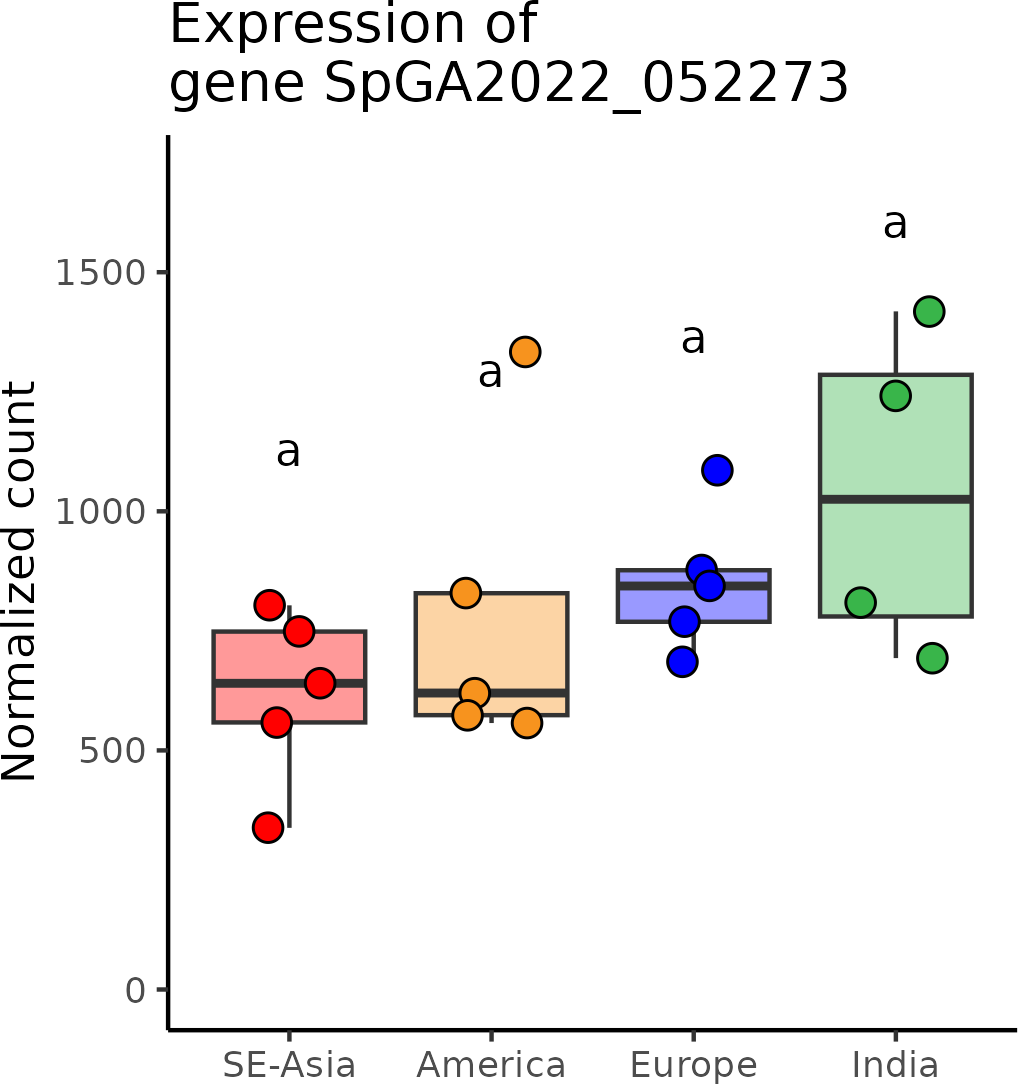


# SpGA2022_007306 (*SOC1*)

**Putative function:** Similar to AGL14: Agamous-like MADS-box protein AGL14 (*Arabidopsis thaliana*)

***Arabidopsis* ortholog/homolog:** AT2G45660.1

**Alignment:**


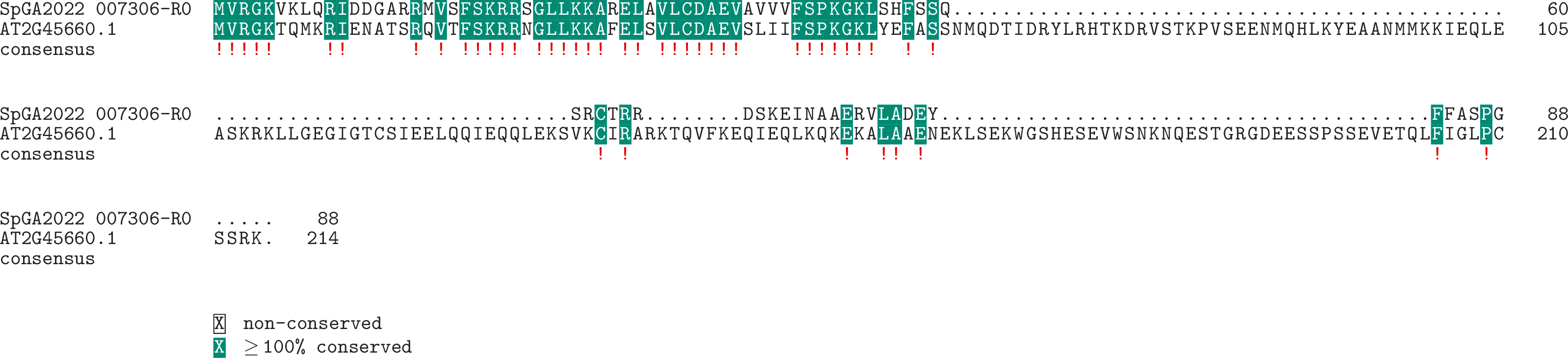


**Expression:**


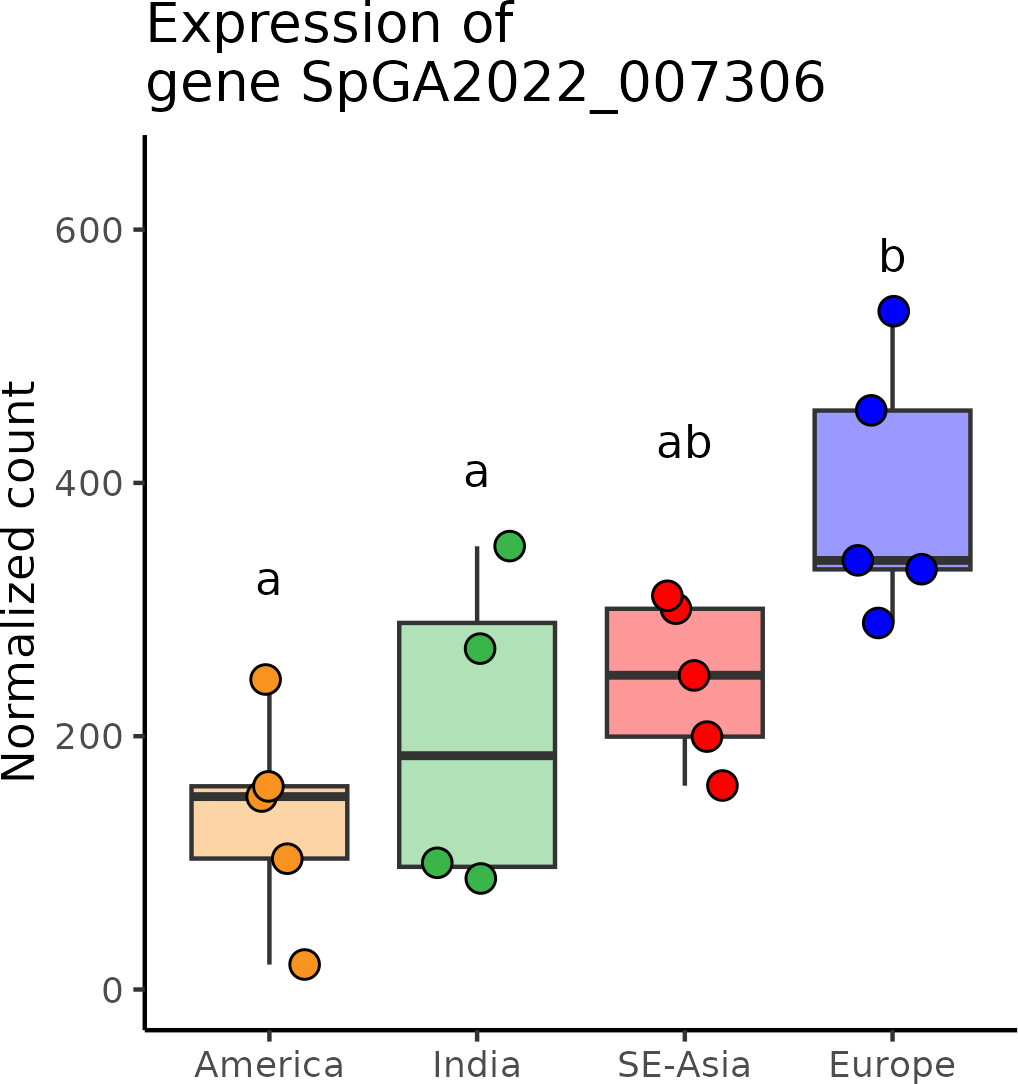


# SpGA2022_006905 (*SVP-*group)

**Putative function:** Similar to AGL24: MADS-box protein AGL24 (*Arabidopsis thaliana*)

***Arabidopsis* ortholog/homolog:** AT2G22540.1

**Alignment:**


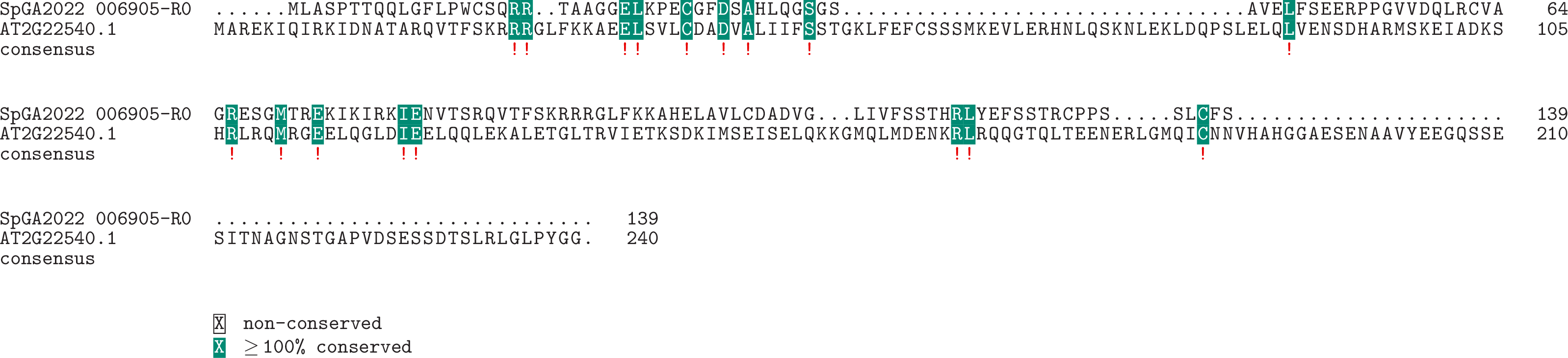


**Expression:**


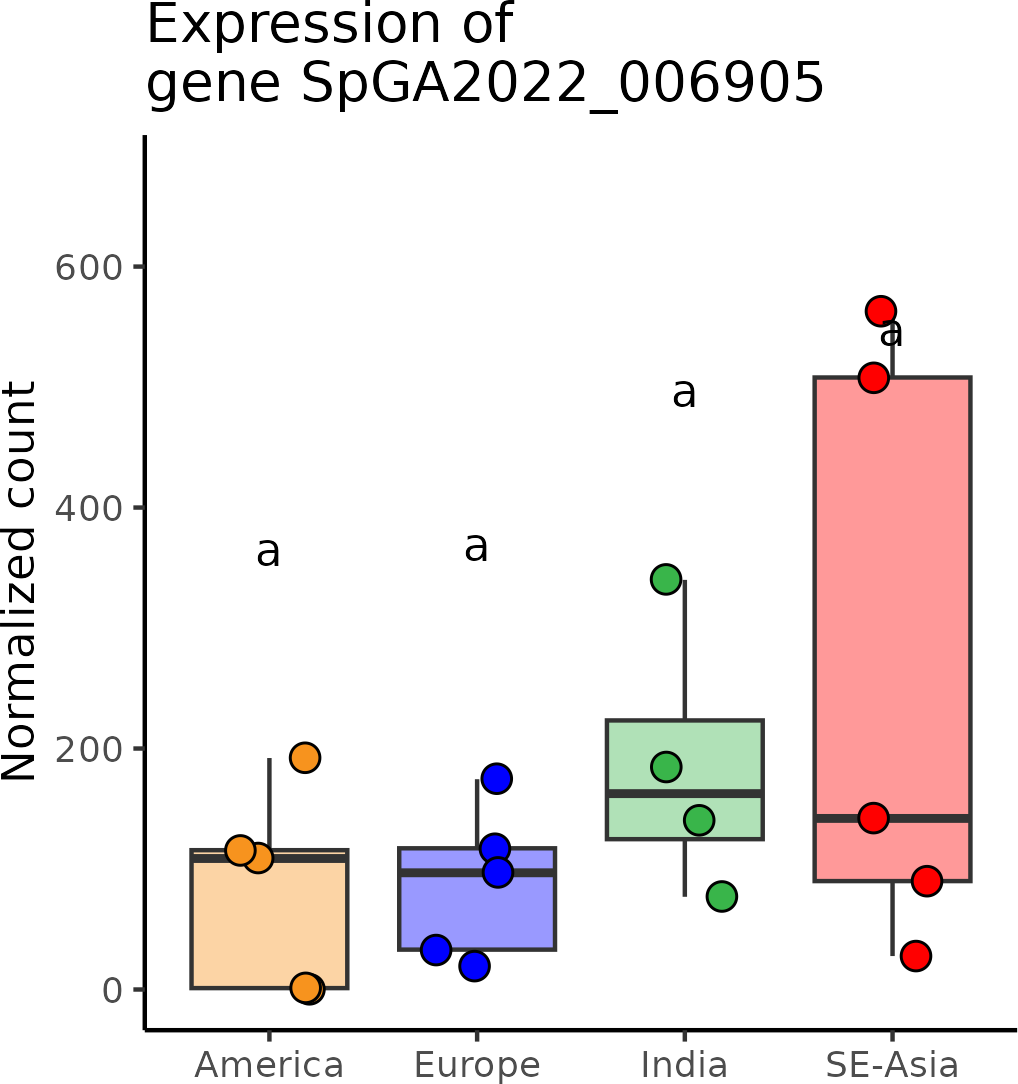


# SpGA2022_051406 (*Transducin/WD40*)

**Putative function:** Protein of unknown function

***Arabidopsis* ortholog/homolog:** AT3G09080.3

**Alignment:**


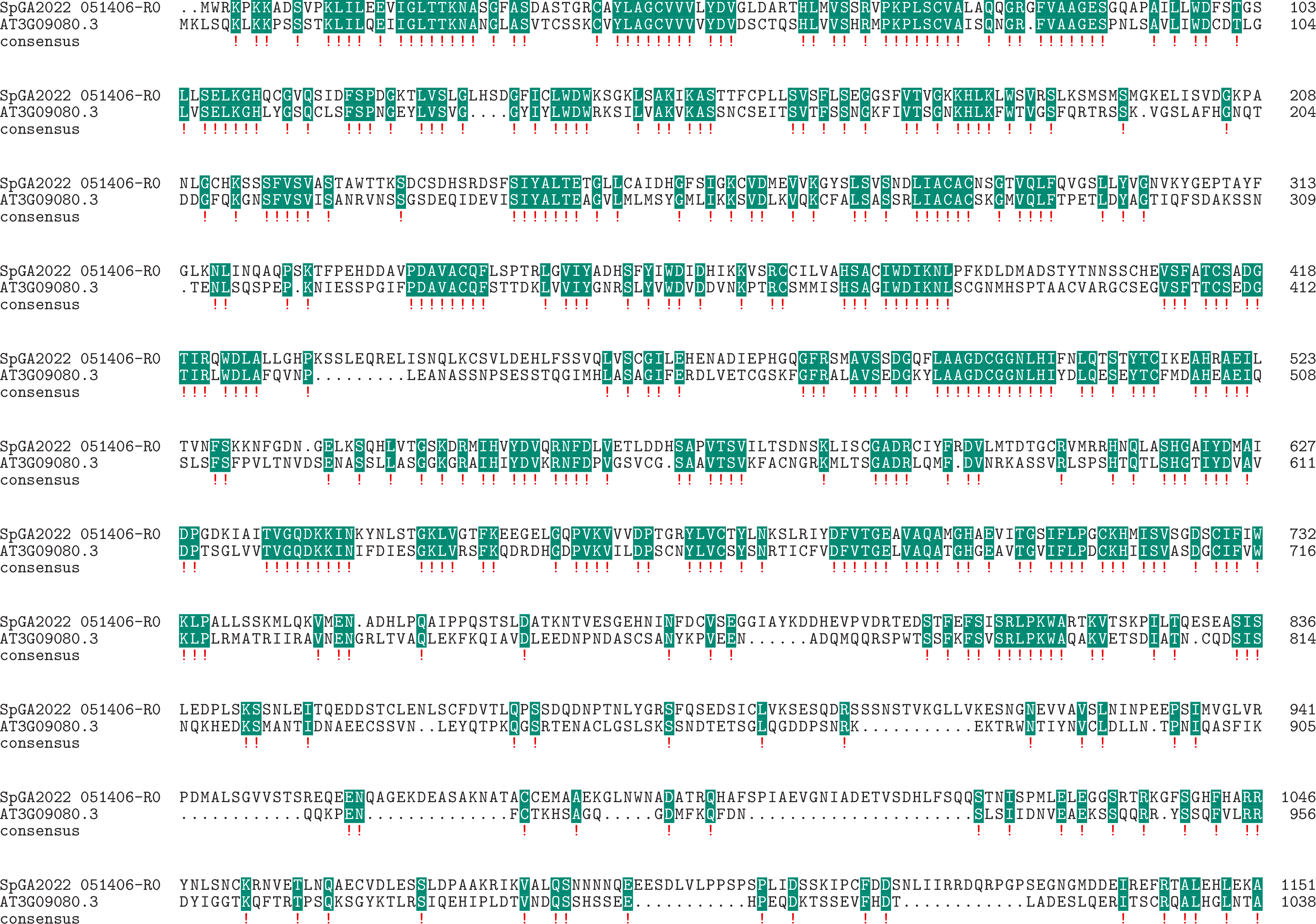


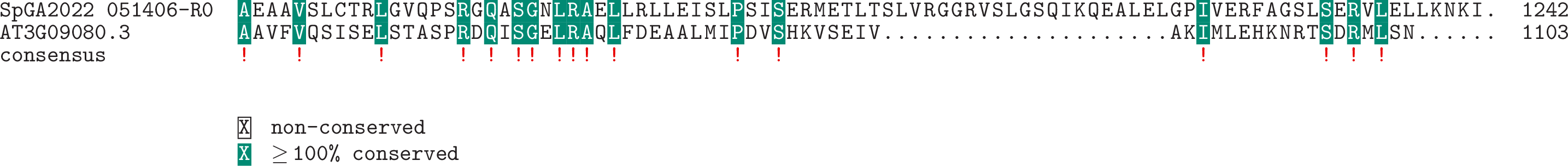


**Expression:**


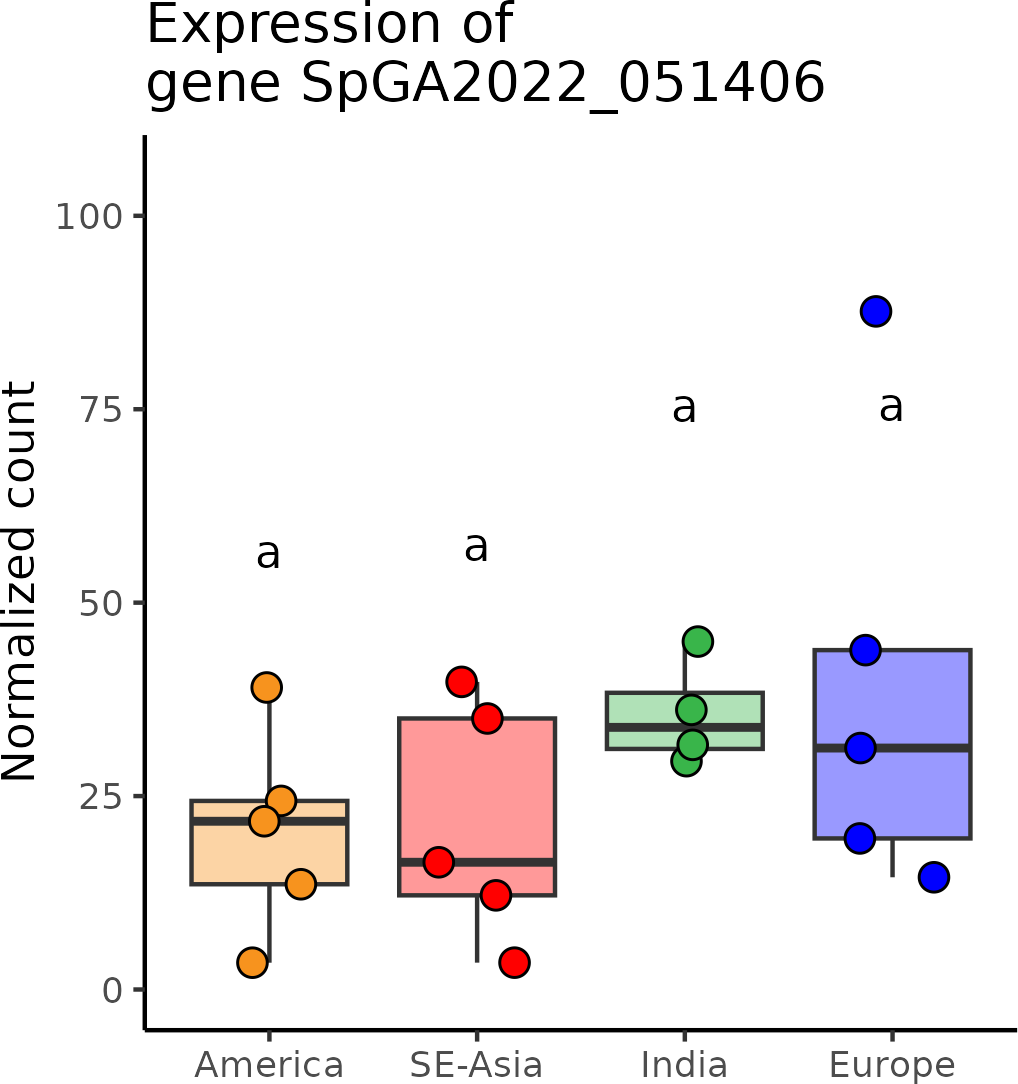


# SpGA2022_055984 (*WAVY*)

**Putative function:** Similar to WAV2: Alpha/beta hydrolase domain-containing protein WAV2 (*Arabidopsis thaliana*)

***Arabidopsis* ortholog/homolog:** AT5G20520.1

**Alignment:**


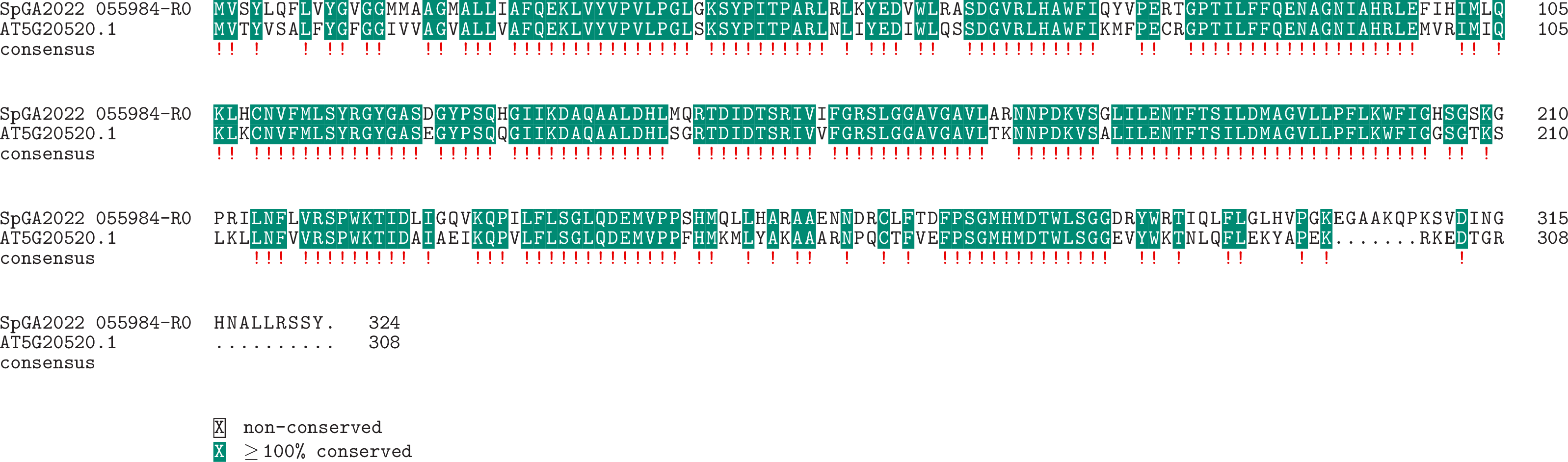


**Expression:**


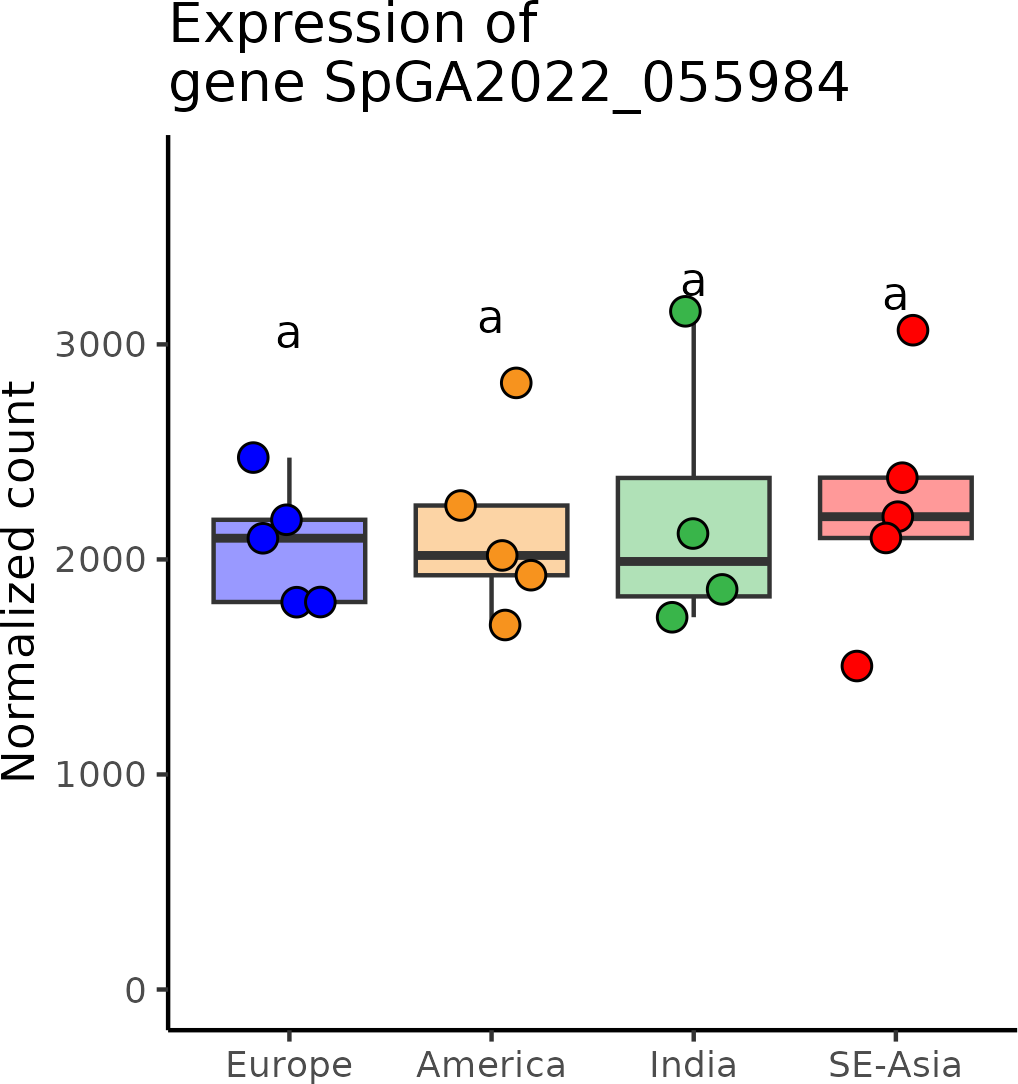


# SpGA2022_014600 (*Xrcc3*)

**Putative function:** Similar to XRCC3: DNA repair protein XRCC3 homolog (*Arabidopsis thaliana*)

***Arabidopsis* ortholog/homolog:** AT5G57450.1

**Alignment:**


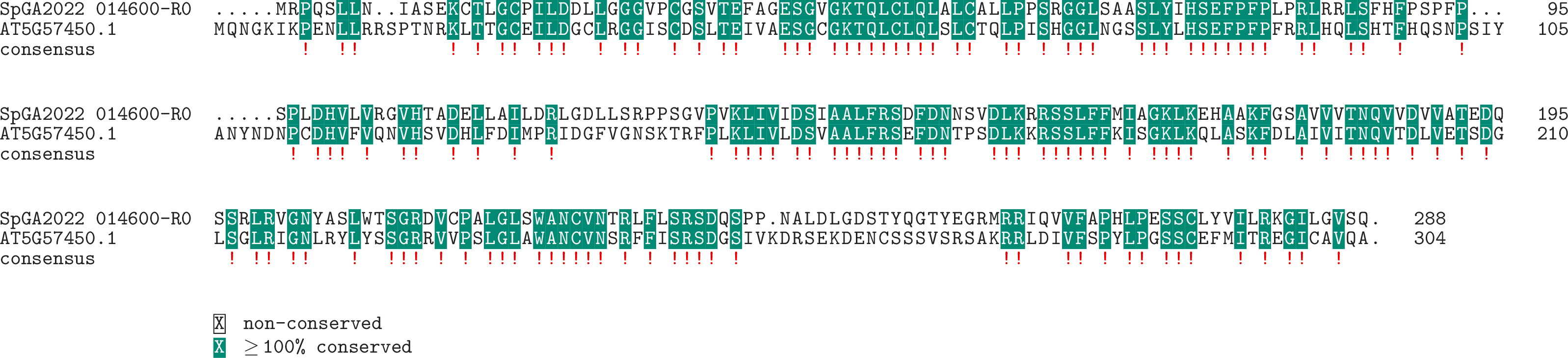


**Expression:**


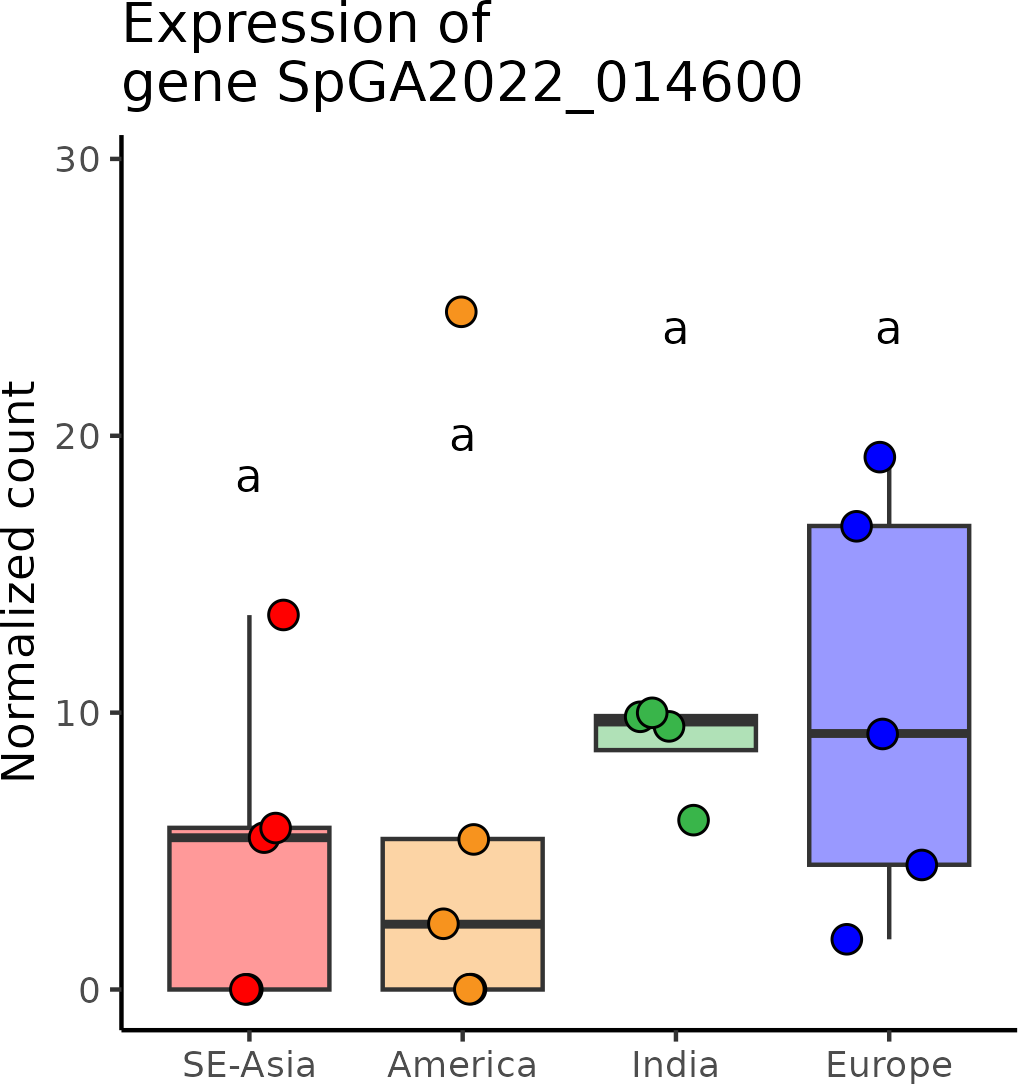


# SpGA2022_051355 (*phospholipase_D*)

**Putative function:** Similar to PLDBETA1: Phospholipase D beta 1 (*Arabidopsis thaliana*)

***Arabidopsis* ortholog/homolog:** AT2G42010.2

**Alignment:**


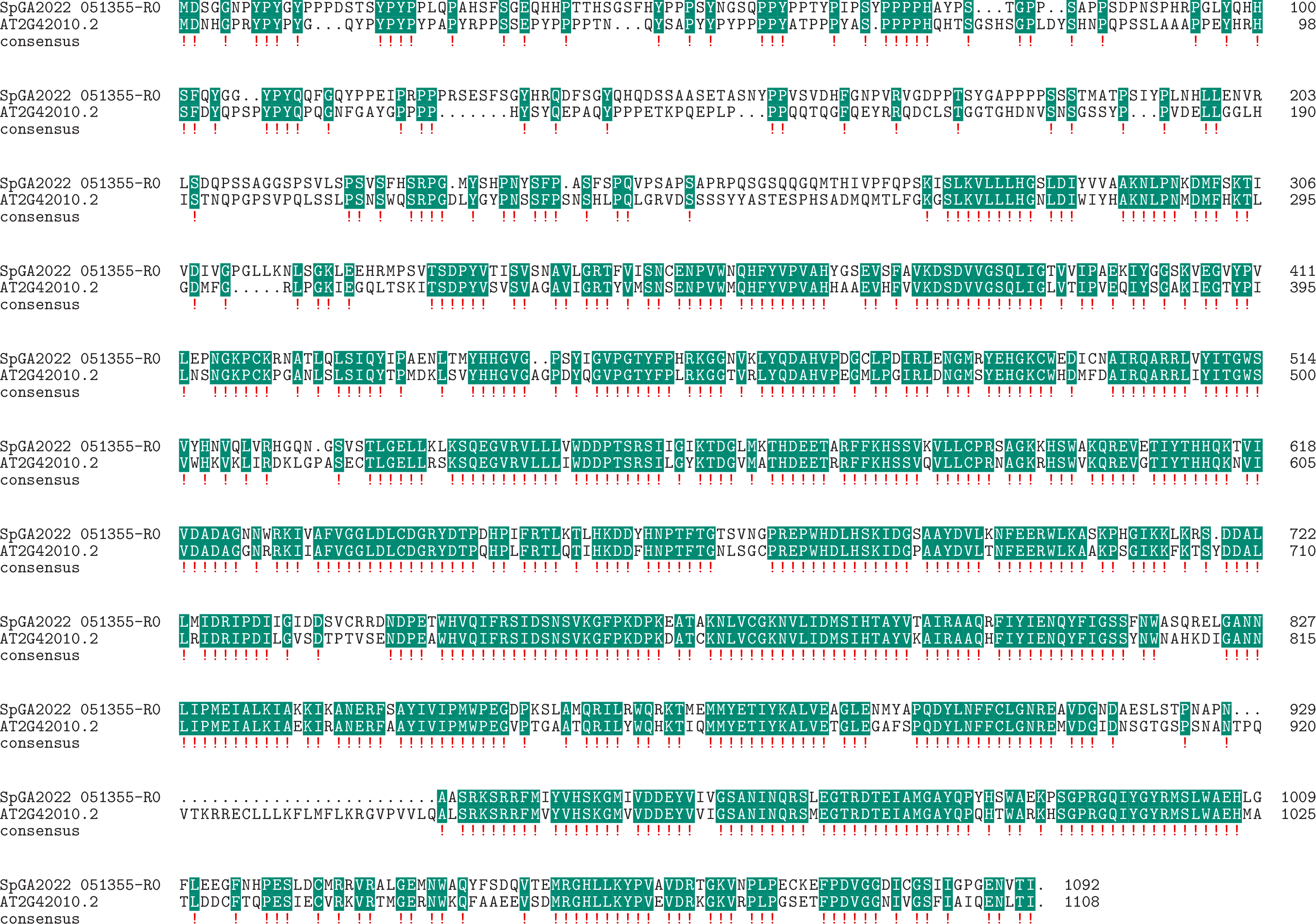


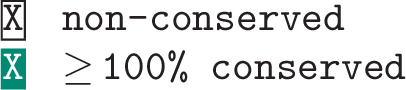


**Expression:**


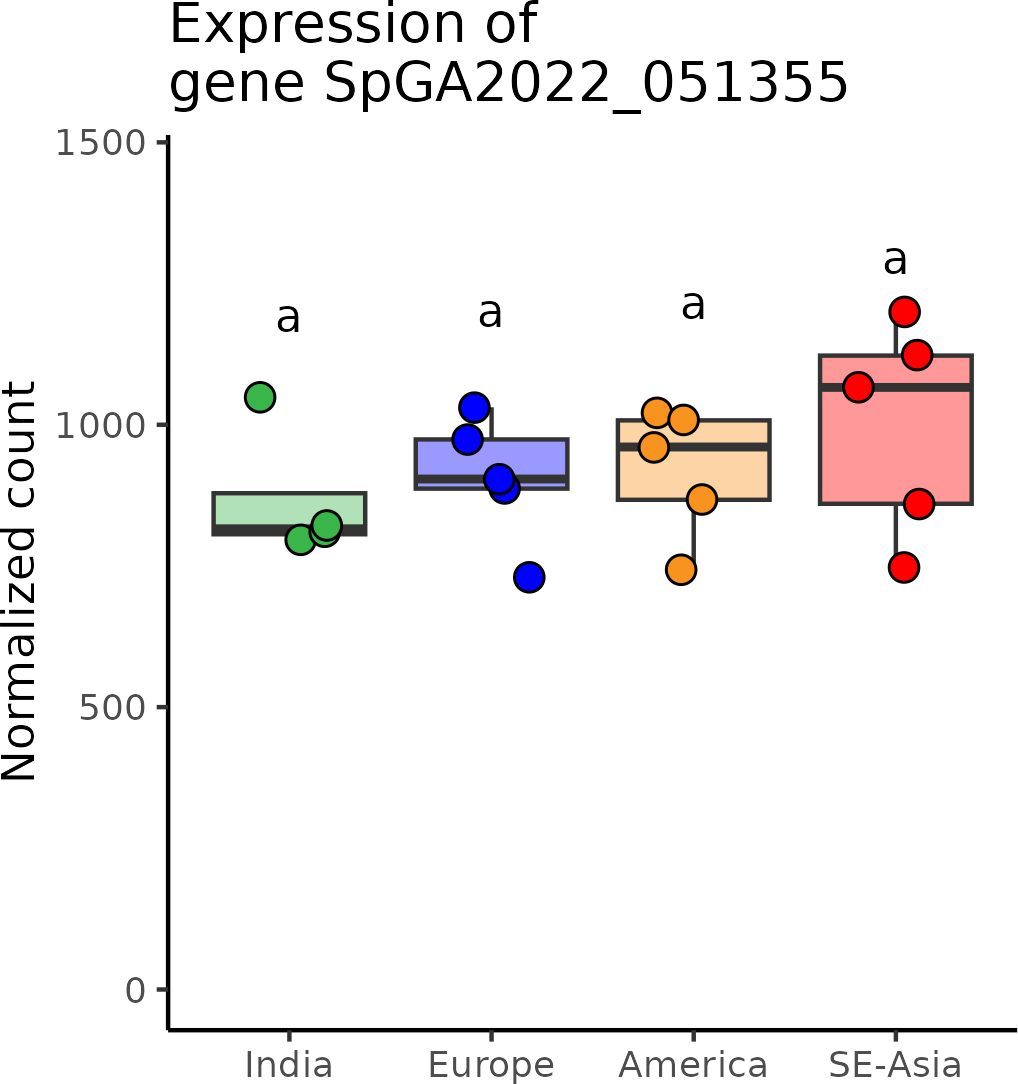

Supplement: Supplementary file 4 — Supplementary Data 1-12 [file 42003_2024_6266_MOESM4_ESM.zip › Supplementary_data/Supplementary_Data_7.docx]
