# Supplementary material for: Population genomics and epigenomics of Spirodela polyrhiza provide insights into the evolution of facultative asexuality
Source: Commun Biol. 2024 May 16;7:581. doi: 10.1038/s42003-024-06266-7 (PMC11099151; doi:10.1038/s42003-024-06266-7)
Supplement: Supplementary file 5 — Reporting Summary [file 42003_2024_6266_MOESM5_ESM.pdf]

nature portfolio

Corresponding author(s): Shuqing Xu
Last updated by author(s): Apr 15, 2024

Reporting Summary

Nature Portfolio wishes to improve the reproducibility of the work that we publish. This form provides structure for consistency and transparency in reporting. For further information on Nature Portfolio policies, see our Editorial Policies and the Editorial Policy Checklist.

Statistics

For all statistical analyses, confirm that the following items are present in the figure legend, table legend, main text, or Methods section.

n/a	Confirmed	
☐	☒	The exact sample size (n) for each experimental group/condition, given as a discrete number and unit of measurement
☐	☒	A statement on whether measurements were taken from distinct samples or whether the same sample was measured repeatedly
☐	☒	The statistical test(s) used AND whether they are one- or two-sided<br>*Only common tests should be described solely by name; describe more complex techniques in the Methods section.*
☐	☒	A description of all covariates tested
☐	☒	A description of any assumptions or corrections, such as tests of normality and adjustment for multiple comparisons
☐	☒	A full description of the statistical parameters including central tendency (e.g. means) or other basic estimates (e.g. regression coefficient) AND variation (e.g. standard deviation) or associated estimates of uncertainty (e.g. confidence intervals)
☐	☒	For null hypothesis testing, the test statistic (e.g. F, t, r) with confidence intervals, effect sizes, degrees of freedom and P value noted<br>*Give P values as exact values whenever suitable.*
☐	☒	For Bayesian analysis, information on the choice of priors and Markov chain Monte Carlo settings
☐	☒	For hierarchical and complex designs, identification of the appropriate level for tests and full reporting of outcomes
☐	☒	Estimates of effect sizes (e.g. Cohen's d, Pearson's r), indicating how they were calculated

Our web collection on statistics for biologists contains articles on many of the points above.

Software and code

Policy information about availability of computer code

Data collection	Code used for analysis are described in details in the method section.
Data analysis	We provide the scripts for data analysis in our github repository: https://github.com/Xu-lab-Evolution/Great_duckweed_popg

For manuscripts utilizing custom algorithms or software that are central to the research but not yet described in published literature, software must be made available to editors and reviewers. We strongly encourage code deposition in a community repository (e.g. GitHub). See the Nature Portfolio guidelines for submitting code & software for further information.

Data

Policy information about availability of data

All manuscripts must include a data availability statement. This statement should provide the following information, where applicable:

- Accession codes, unique identifiers, or web links for publicly available datasets
- A description of any restrictions on data availability
- For clinical datasets or third party data, please ensure that the statement adheres to our policy

The raw genomic and bisulfite sequencing reads involved in this study can be retrieved from NCBI under Accession numbers Bioproject PRJNA701543 and Bioproject PRJNA934173.

Research involving human participants, their data, or biological material

Policy information about studies with human participants or human data. See also policy information about sex, gender (identity/presentation), and sexual orientation and race, ethnicity and racism.

Reporting on sex and gender	Not applicable
Reporting on race, ethnicity, or other socially relevant groupings	Not applicable
Population characteristics	Not applicable
Recruitment	Not applicable
Ethics oversight	Not applicable

Note that full information on the approval of the study protocol must also be provided in the manuscript.

Field-specific reporting

Please select the one below that is the best fit for your research. If you are not sure, read the appropriate sections before making your selection.

☐ Life sciences ☐ Behavioural & social sciences ☒ Ecological, evolutionary & environmental sciences

For a reference copy of the document with all sections, see nature.com/documents/nr-reporting-summary-flat.pdf

Ecological, evolutionary & environmental sciences study design

All studies must disclose on these points even when the disclosure is negative.

Study description	We sequenced the genomes of 131 globally distributed S. polyrhiza genotypes with an average of ~25 X coverage. Together with previously published samples, we analyzed the genomic diversity of 228 S. polyrhiza individuals across five continents.
Research sample	As we aim to understand the global diversity and evolution of Spirodela polyrhiza, we sequenced all available Spirodela polyrhiza samples to us at the time.
Sampling strategy	After our initial study on the genomic diversity of this species (Xu et al. 2019, Nat Comm) suggested that SE-Asia population has higher genomic diversity, we took additional effort in collecting more samples from this area.
Data collection	All genomic data were obtained using Illumina sequencers. Raw data are deposited in NCBI under accession numbers Bioproject PRJNA701543 and Bioproject PRJNA934173
Timing and spatial scale	The samples were collected globally. The samples were collected at different time as this is part of global duckweed collection effort. The origin of each sample are shown in supplementary data 1.
Data exclusions	After sequencing, we excluded three samples had had very low mapping rate (less than 50%) to Spirodela polyrhiza reference genome. This is due to the mis-identification of the species.
Reproducibility	We repeated the analysis using the data from our previous dataset (Xu et al. 2019, Nat Comm). The results were consistent.
Randomization	Not applicable.
Blinding	During the data analysis, we first removed the sample origins for the population structure analysis. Then, we mapped the sample origin to the population structures identified using the Structure analysis.

Did the study involve field work? ☐ Yes ☒ No

Reporting for specific materials, systems and methods

We require information from authors about some types of materials, experimental systems and methods used in many studies. Here, indicate whether each material, system or method listed is relevant to your study. If you are not sure if a list item applies to your research, read the appropriate section before selecting a response.

Materials & experimental systems

n/a	Involved in the study
☒	☐ Antibodies
☒	☐ Eukaryotic cell lines
☒	☐ Palaeontology and archaeology
☒	☐ Animals and other organisms
☒	☐ Clinical data
☒	☐ Dual use research of concern
☐	☒ Plants

Methods

n/a	Involved in the study
☒	☐ ChIP-seq
☒	☐ Flow cytometry
☒	☐ MRI-based neuroimaging

Dual use research of concern

Policy information about dual use research of concern

Hazards

Could the accidental, deliberate or reckless misuse of agents or technologies generated in the work, or the application of information presented in the manuscript, pose a threat to:

No	Yes
☒	☐ Public health
☒	☐ National security
☒	☐ Crops and/or livestock
☒	☐ Ecosystems
☒	☐ Any other significant area

Experiments of concern

Does the work involve any of these experiments of concern:

No	Yes
☒	☐ Demonstrate how to render a vaccine ineffective
☒	☐ Confer resistance to therapeutically useful antibiotics or antiviral agents
☒	☐ Enhance the virulence of a pathogen or render a nonpathogen virulent
☒	☐ Increase transmissibility of a pathogen
☒	☐ Alter the host range of a pathogen
☒	☐ Enable evasion of diagnostic/detection modalities
☒	☐ Enable the weaponization of a biological agent or toxin
☒	☐ Any other potentially harmful combination of experiments and agents

Plants

Seed stocks	Accessions are stored in my lab at the University of Mainz, Germany
Novel plant genotypes	Not applicable
Authentication	Not applicable
